# Insulin Resistance and Inflammation

**DOI:** 10.3390/ijms27031237

**Published:** 2026-01-26

**Authors:** Evgenii Gusev, Alexey Sarapultsev, Yulia Zhuravleva

**Affiliations:** 1 Institute of Immunology and Physiology, Ural Branch of the Russian Academy of Science, 106 Pervomaiskaya Street, 620049 Ekaterinburg, Russia; a.sarapultsev@gmail.com; 2 A Russian–Chinese Education and Research Center of System Pathology, South Ural State University, 76 Lenin Prospekt, 454080 Chelyabinsk, Russia

**Keywords:** type 2 diabetes, metabolic syndrome, insulin resistance, obesity, MASLD/NAFLD, inflammation, glucotoxicity, lipotoxicity, cellular stress, glucose and lipid transporters

## Abstract

Insulin resistance (IR) is a central driver of cardiometabolic disease and an increasingly recognized modifier of inflammatory and vascular pathology. Beyond impaired glucose homeostasis, IR emerges from chronic, metabolically induced inflammation (“meta-inflammation”) and convergent cellular stress programs that propagate across tissues and organ systems, ultimately shaping endothelial dysfunction, atherogenesis, and cardiometabolic complications. Here, we synthesize multilevel links between insulin receptor signaling, intracellular stress modules (oxidative, endoplasmic reticulum, inflammatory, and fibrotic pathways), tissue-level dysfunction, and systemic inflammatory amplification. This work is a conceptual narrative review informed by targeted database searches and citation tracking, with explicit separation of mechanistic/experimental evidence from human observational and interventional data; causal inferences are framed primarily on mechanistic and interventional findings, whereas associative statements are reserved for observational evidence. We propose an integrative framework in which stress-response pathways are context-dependent and become maladaptive when chronically activated under nutrient excess and persistent inflammatory cues, generating self-reinforcing loops between IR and inflammation that accelerate vascular injury. This framework highlights points of convergence that can guide mechanistic prioritization and translational hypothesis testing.

## 1. Introduction

Insulin resistance (IR) is a chronic disorder characterized by an impaired metabolic response to insulin. This condition results in compensatory elevations of circulating insulin relative to the prevailing glucose concentration [1,2,3].

Traditionally, IR is viewed as a pathogenic platform for visceral (morbid) obesity, the metabolic syndrome, and type 2 diabetes mellitus (T2DM). Diabetes is a leading cause of mortality and disability worldwide, and T2DM accounts for 90–95% of all diabetes cases [4]. The clinical spectrum of complications includes metabolically associated steatosis liver disease (MASLD), formerly known as nonalcoholic fatty liver disease (NAFLD), with steatohepatitis as the most severe stage of MASLD; atherosclerosis; hypertension; cardiomyopathies; sarcopenia; diabetic kidney disease; diabetic eye disease (retinopathy, cataract, glaucoma, retinal detachment, vitreous hemorrhage); and diabetic encephalopathy and polyneuropathy, as well as other somatic and neuropsychiatric disorders associated with IR and T2DM [5,6,7,8,9,10]. Thus, IR is clinically relevant not only as a metabolic phenotype but also as a broad risk context spanning hepatic, vascular, renal, ocular, and neural endpoints.

Prior reviews provide complementary frames for this spectrum by linking obesity-associated inflammation to IR and its vascular and hepatic complications. Jung and Choi summarize the adipokine-centered view of obesity as an endocrine/inflammatory condition that is coupled to IR and MASLD/NAFLD [5]. Galicia-Garcia et al. provide an integrated overview of T2DM pathophysiology as a combined failure of insulin action and insulin secretion [6]. Kitessa and Abeywardena emphasize that skeletal muscle IR is more closely associated with bioactive lipid intermediates than with total intramuscular fat [7]. Trojnar et al. discuss FABP4 as a proinflammatory adipokine associated with IR and diabetes phenotypes and a candidate biomarker [8]. Kosmas et al. summarize clinical and mechanistic links between IR and cardiovascular disease, supporting an association between IR and vascular risk [9]. Gutiérrez-Cuevas et al. integrate obesity-related mechanisms connecting MAFLD/NASH with cardiovascular disease, positioning T2DM as a frequent bridge between hepatic and vascular pathology [10]. In addition, progression of IR and T2DM may lead to injury, partial loss, and dysfunction of β-cells, resulting in more pronounced insulin deficiency at late stages of T2DM [11,12].

The pathogenesis of IR rests on a mismatch between insulin receptor (InsRec) signaling and other molecular regulatory pathways in insulin-responsive organs—principally the liver, adipose tissue, and skeletal muscle—as well as on impaired insulin production by β-cells [13]. Key manifestations include pathological upregulation of gluconeogenesis (liver); defective protein and glycogen synthesis (liver and muscle); dysregulated lipid synthesis and breakdown (liver, adipose tissue) and the formation of lipid transport particles in the liver; and reduced membrane expression, in adipocytes and resting muscle, of the insulin-dependent glucose transporter type 4 (GLUT4) [14,15,16]. IR-associated visceral obesity features large adipocytes that are resistant to insulin and a relative depletion of small adipocytes, which display greater insulin sensitivity [17,18,19]. The primary driver of visceral obesity is an imbalance between intake and utilization of energy-dense metabolites (lipids and carbohydrates), the excess of which is converted to triacylglycerol (TAG) in the liver and in adipocytes [20,21]. These dysfunctions are accompanied by changes in circulating insulin or C-peptide and a range of biochemical indices of lipid and carbohydrate metabolism that can operationalize IR in clinical settings. Several indices are currently used, including HOMA-IR (insulin × glucose), HOMA2-IR (C-peptide × glucose), HOMA-B (insulin/glucose), Matsuda Index (glucose/insulin), QUICKI (1/[insulin + glucose]), TyG (TAG × glucose), MCAi (insulin − TAG), and TAG/HDL, which rely on measurements of glucose, insulin or C-peptide, TAG, and high-density lipoproteins (HDL) [22,23].

At the cellular level, InsRec signaling—particularly the phosphoinositide 3-kinase (PI3K)/protein kinase B (PKB, or Akt) pathway—interacts with multiple inflammatory signaling cascades and processes. Perturbations across these coupled pathways can disrupt InsRec function and contribute to the development of IR [24,25,26,27].

Addressing IR is challenging because inflammation is a multifaceted, variably expressed general pathological process, and because many additional molecular mechanisms contribute to IR, the following interlocking domains:

- The context-dependent roles of intracellular lipid sensors, notably the peroxisome proliferator-activated receptors (PPARs), in IR development.
- Complex interactions among receptors for glucose and free fatty acids (FFAs), as well as among FFA receptors and transport systems.
- Functional integration of insulin-responsive organs not only through metabolic cycles (the Cori cycle, the Randle cycle, and the alanine cycle) but also through their organokines (adipokines/lipokines, myokines, hepatokines, cytokines) and extracellular vesicles containing microRNAs (miRNAs).
- Crosstalk of these organs with the central nervous system, the immune system, the microbiome, and the intestinal barrier.

All of these processes are interwoven with pro-inflammatory mechanisms at the cellular, organ, and organismal levels and, in many instances, constitute integral components of those mechanisms. Accordingly, an integrative systems-level synthesis is useful for distinguishing recurrent mechanistic motifs from context-specific variants.

These considerations support the need to develop an integrated view of the systemic interrelationship between inflammation and IR, which is the primary objective of this study. As a necessary preliminary step, we further refine the concepts of cellular and tissue stress, their physiological and pathological roles, and their relationship to distinct forms of inflammation.

## 2. Methods

This article is a conceptual narrative review developed within a systems-science framework [28,29] to integrate multilevel evidence linking insulin resistance to cellular and tissue stress responses and inflammation, and to place these links in the context of vascular dysfunction and atherosclerosis. Literature was identified through targeted searches in PubMed, Web of Science, Scopus, and Google Scholar, complemented by backward and forward citation tracking of key primary studies and authoritative reviews. The last database update was performed on 1 October 2025.

Search terms were constructed around insulin resistance and related metabolic phenotypes (e.g., obesity, metabolic syndrome, type 2 diabetes, MASLD/NAFLD) and major mechanistic domains (e.g., meta-inflammation, cytokines and chemokines, oxidative stress, endoplasmic reticulum stress, glucotoxicity/lipotoxicity, fibrosis, stress-response signaling pathways, endothelial dysfunction, microbiome-related mechanisms, and atherosclerosis). No formal date limits were applied at the retrieval stage to capture both seminal mechanistic work and contemporary updates. At the synthesis and writing stages, we preferentially weighted literature from the last two decades for terminology, methodological standards, and translational framing, while retaining earlier primary studies when they represent original mechanistic demonstrations or canonical pathway descriptions.

Source selection was guided by topical relevance, conceptual contribution, and evidentiary weight. We prioritized primary mechanistic studies to support pathway-level statements, a limited set of high-quality contemporary reviews and consensus statements for orientation and synthesis (not as sole support for mechanistic claims), and human observational or interventional studies when discussing clinical associations and therapeutic implications. Given the integrative scope, we retained a comprehensive reference corpus to preserve traceability across subdomains; however, citations were not treated as equivalent in evidential strength. Accordingly, we explicitly distinguish mechanistic/experimental evidence from human associative and interventional evidence in the text and, where applicable, in tables.

Because the goal was integrative conceptual systematization rather than exhaustive systematic mapping, we did not implement a PRISMA workflow, predefined effect-size extraction, or a single standardized risk-of-bias tool across all study types.

## 3. Cellular and Tissue Stress and Their Impact on Insulin Function

### 3.1. General Concepts of Cellular and Tissue Stress

From an evolutionary perspective, cellular stress (CS) represents a protective mechanism present in all cell types, beginning with prokaryotes [30]. CS is a response to any form of macromolecular damage that exceeds a defined threshold, irrespective of the initial cause. It relies on a phylogenetically conserved set of genes and pathways that stabilize macromolecules and restore altered parameters of homeostasis, thereby preserving cellular and organismal integrity under suboptimal conditions [31,32].

In humans, CS is particularly apparent in cells specialized for inflammation (e.g., leukocytes, macrophages, platelets, mast cells, and HEV endothelium) [24]. In these cells, CS is robustly induced by high-intensity inflammatory cues, including hemostatic/complement activation products; PAMP/DAMP sensing by pattern-recognition receptors (e.g., TLRs); high concentrations of cytokines and other mediators; and antigen-driven lymphocyte activation [33,34,35,36]. Such high-amplitude activation underlies classical inflammatory phenotypes and, in extreme cases, shock states during acute systemic hyperinflammation [33,34,35,36].

Beyond inflammation-specific triggers, CS can be driven by broadly acting homeostatic disturbances, including macromolecular injury, glucotoxicity/lipotoxicity, chronic hypoxia, energetic stress (an increased AMP/ADP/ATP ratio), redox imbalance, and electrolyte shifts that threaten cellular viability. These stimuli operate across diverse cell types and tissues. Depending on context and intensity, CS may remain adaptive (supporting survival and functional compensation) or become maladaptive, converging on cell-cycle arrest, apoptosis, regulated necrosis, senescence, altered differentiation states, or recruitment into defined inflammatory programs [33].

**Figure 1:**
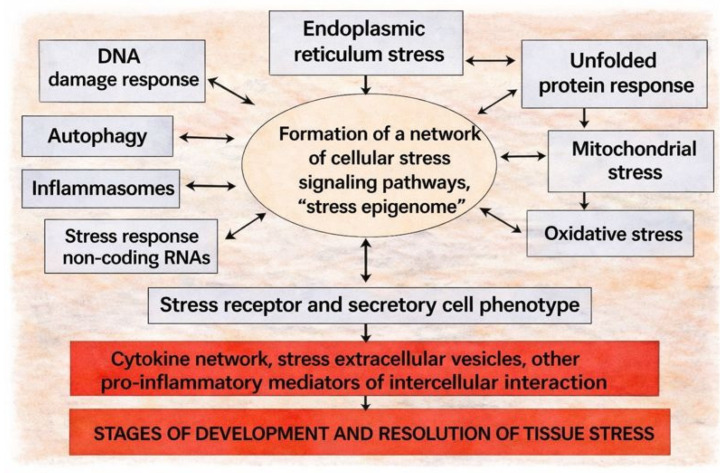
The structure of typical cellular stress processes and their relationship with tissue stress. Note: The unfolded protein response (UPR) in mitochondria and the endoplasmic reticulum (ER) coordinates transient translation attenuation, clearance of damaged proteins, and induction of chaperones (notably heat-shock proteins) [37,38]. Stress-responsive non-coding RNAs (including microRNAs) modulate transcription/translation and can mediate intercellular signaling via extracellular vesicles [39,40,41]. Inflammasomes drive IL-1β/IL-18 maturation and pyroptosis [42,43]. Macroautophagy clears damaged macromolecular complexes (including inflammasomes) and dysfunctional mitochondria (mitophagy) [44,45]. The DNA-damage response integrates DNA repair and checkpoint control across the cell cycle [46,47]. Oxidative stress reflects excessive free-radical production with macromolecular injury when redox balance is lost [48,49,50]. Mitochondrial stress and ER stress represent integrated responses to organelle dysfunction that intersect with oxidative stress, UPR, apoptosis, and autophagy [51,52,53,54,55,56].

Figure 1 shows a complex of universal CS components that are interconnected by a network of signaling pathways; intercellular interactions form a more complex phenomenon, namely, various types of tissue stress.

During CS, the expression of thousands of genes can change [57,58]. Stress signaling is therefore organized as a network, where inducible genes and shared mediators participate in multiple pathways and are coordinated through stress-responsive epigenetic regulation [59,60,61,62,63,64,65].

Key hubs of CS signaling include NF-κB; MAPKs (ERK, JNK, p38); and cytokine-linked JAK/STAT pathways [66,67,68,69,70,71,72,73]. In disease, these axes are commonly associated with inflammation, tumor-cell survival, and tissue aging [34,74,75,76,77,78,79,80,81], whereas under constitutive or moderate activation, they contribute to normal survival programs, differentiation, metabolic regulation, and homeostasis [82,83,84,85,86,87,88,89,90,91,92,93]. Consequently, shared nodes (e.g., PI3K/Akt, JAK/STAT, MAPK, NF-κB, AMPK, PKC isoforms) can operate in physiology or inflammation depending on context.

### 3.2. Physiological Roles of Cellular and Tissue Stress

Even under physiological conditions, cells rarely exist in perfectly optimal microenvironments, and low-intensity damaging inputs (including stochastic mutational events) are unavoidable. Accordingly, CS is continuously coupled to metabolic homeostasis, cell-cycle control, differentiation, and other core physiological functions. At the tissue level, cytokines contribute to coordination of these processes; many are constitutively expressed at low levels and increase modestly with physiological stimuli. Physiological roles have been documented for multiple “pro-inflammatory” cytokines (e.g., interferons, TNF-α, IL-1β, IL-6, IL-8, IL-17) as well as for anti- and context-dependent mediators (e.g., IL-10, IL-4) [94,95,96,97,98,99,100,101,102,103,104]. Prostaglandins likewise exert broad physiological actions beyond inflammation [105].

A relatively stable, low-level engagement of CS pathways in healthy tissues can be viewed as a basal “stress tone.” A moderate, time-limited increase in this tone supports adaptation and resistance to subsequent insults, whereas a sustained or spatially extensive increase may transition into inflammation. Higher basal tone is typical of barrier and mucosal interfaces and lymphoid organs, where constant antigen exposure and physiological lymphocyte turnover sustain activation cues [24]. By contrast, barrier-protected tissues such as the CNS generally operate at a lower inflammatory tone.

Nevertheless, CNS neurons remain vulnerable to homeostatic disturbances such as electrolyte imbalance; excessive glutamatergic signaling can induce excitotoxicity and neuronal loss [106]. Neurotransmitters act not only via ionotropic channels but also through metabotropic GPCRs that engage core survival and metabolic nodes (PI3K/Akt and MAPK/ERK, with downstream small GTPases and PLC/PKC signaling) and typically only moderate NF-κB activation under physiological conditions [107]. These nodes are shared across many GPCRs and receptor tyrosine kinases, including the insulin receptor (InsRec), and thereby provide a mechanistic bridge between “stress” signaling and physiological neuronal maintenance. In addition, low-to-moderate cytokine signaling contributes to CNS homeostasis via JAK/STAT pathways [108]. Persistent psychosocial stress may create localized zones of tissue stress and can contribute to low-grade neuroinflammation [109]. Insulin and multiple growth factors acting via RTKs support neuronal survival programs [110,111,112].

Intense muscular activity provides another example of an extreme physiological process involving tissue stress. CS mechanisms sustain skeletal-muscle function under transiently altered homeostasis, including lactate accumulation, ATP deficit, electrolyte shifts, local hyperthermia, and related changes [113,114,115,116,117,118]. Contracting muscle releases large amounts of myokines, including classically pro-inflammatory cytokines, into the circulation. Notably, circulating IL-6 can rise markedly (reported up to ~100-fold) during intense exercise without clinical evidence of muscle damage [119]. This response contributes to systemic adaptations, including activation of innate immunity [120]. Under physiological conditions, these stress responses resolve rapidly; muscle-derived anti-inflammatory mediators contribute to resolution and scale positively with training status [121]. Skeletal muscle is metabolically insulin-dependent, and sustained pro-inflammatory CS is generally unfavorable for InsRec signaling.

However, during exercise, glucose uptake is maintained through insulin-independent GLUT4 translocation, with AMPK serving as a principal upstream integrator [122,123,124]. In resting muscle and other insulin-sensitive tissues, insulin-dependent PI3K/Akt2 signaling—via AS160/TBC1D4 and Rab GTPases—predominates in GLUT4 trafficking and glucose uptake [125,126].

Normal pregnancy—often accompanied by moderate shifts in cytokine tone—provides another example of a time-limited extreme physiological state associated with tissue stress [127,128,129].

In sum, CS signaling supports a wide range of physiological processes within basal tissue tone and during time-limited extreme physiological states. Therefore, CS pathways cannot be classified as uniformly “beneficial” or “dysfunctional,” and indiscriminate anti-inflammatory/antioxidant intervention may have context-dependent consequences [130,131,132,133,134,135,136,137,138].

### 3.3. General Regularities of Cellular and Tissue Stress Related to Insulin and Other Receptor Tyrosine Kinase Ligands

Receptor tyrosine kinases (RTKs) activate intracellular signaling pathways that regulate metabolism—predominantly anabolism—as well as growth, differentiation, migration, and survival of both proliferating and post-mitotic cells. Besides insulin, typical RTK ligands include various growth factors. Common downstream components of RTK signaling comprise PI3K/Akt/mTOR, MAPK–ERK, PLCγ/DAG/PKC, Ras GTPases, and context-dependent modulation of p53 activity [139,140,141,142,143,144,145,146,147,148]. As anabolic activators, RTKs can restrain autophagy (via Akt/mTOR) and catabolism through inhibition of FOXO transcription factors and other mechanisms [149,150,151]. Importantly, RTK signaling frequently intersects with stress and inflammatory nodes (e.g., JNK/p38, NF-κB, ROS generation), which can be engaged at low intensity in physiology but escalate under pathological load. RTKs also support oncogenesis through growth promotion, anabolic effects, and attenuation of p53-mediated apoptosis via the PI3K/Akt/p53 axis. In addition, RTKs—including InsRec—activate phospholipase C-γ (PLCγ), leading to the formation of inositol 1,4,5-trisphosphate (IP3) and diacylglycerol (DAG), which in turn activate calcium channels and PKC, with broad physiological and pathological consequences [146,147,148]. These canonical modules are shared across many RTKs and GPCRs and therefore provide a common “signaling vocabulary” for hormones, growth factors, neurotransmitters, regulatory metabolites, chemokines, and other inflammatory mediators. The specific outputs of these pathways are determined by cell type; subcellular localization of receptors and signaling modules; differences in downstream components within shared cascades; pathway crosstalk; the abundance of isoforms and post-translational modifications; and the redundancy of regulatory sites (e.g., multiple phosphorylation sites within a single protein). Together, these features explain how apparently similar pathway “blocks” can generate receptor- and context-specific effects, including for InsRec [152,153,154,155,156,157,158,159,160,161,162,163]. Consequently, engagement of nominally the same pathway can yield divergent—and occasionally opposing—outcomes depending on the cellular and pathological context.

At present, the precise alterations in insulin signaling and GLUT4 trafficking that underlie insulin resistance (IR) remain incompletely defined. The specificity of the PI3K/Akt axis in insulin-stimulated GLUT4 translocation appears to involve multiple PI3K classes; distinct Akt isoforms (Akt1–3); class IA PI3K regulatory subunits (p85α, p85β, p55γ); phosphorylation of Akt at Thr308 by PDPK1; context-dependent activation of downstream effectors such as AS160; and engagement of Akt-independent routes [164,165,166,167,168,169,170,171]. Accordingly, “IR” likely reflects heterogeneous lesions across the insulin–GLUT4 axis rather than a single invariant defect.

Escalation of pro-inflammatory mechanisms shifts the CS paradigm toward cell-cycle blockade; more severe oxidative burden; increased probabilities of apoptosis and necrosis; accelerated cellular senescence; activation of autophagy and other catabolic programs; and differentiation toward a more pro-inflammatory phenotype [172,173,174,175,176,177,178,179,180,181]. When sustained, this pro-inflammatory CS milieu can compromise canonical RTK outputs and, in the case of InsRec, facilitate the emergence of IR [182,183,184]. The metabolic effects of insulin cannot be fully compensated by insulin-like growth factor-1 (IGF-1) or by alternative mechanisms [185,186].

### 3.4. Roles of Insulin and Other RTK Ligands in Metabolic Stress; The Role of AMPK in Insulin Signaling Under Metabolic Stress

Along with GPCRs, RTKs actively participate in metabolic cellular stress (MCS), a metabolic branch of cellular/tissue stress that is tightly coupled to proteostatic programs, including ER stress [187,188]. MCS is induced by elevations or reductions in key metabolite levels and by declines in cellular energy charge, especially ATP depletion [189]. Functionally, MCS represents an adaptive attempt to preserve core cell functions—including proliferation, differentiation, migration, secretion, neuronal signaling, and muscle contraction—and, under pathological conditions, to sustain pro-inflammatory activity [187,190]. The principal MCS nodes are Akt/mTOR (notably mTORC1) and AMPK, which frequently act in opposition.

Akt is activated downstream of multiple receptors and by intracellular cues consistent with relatively high energy availability; mTOR activity is further tuned by amino-acid sufficiency [191]. The main functions of Akt/mTOR during MCS include promotion of mitosis; stimulation of protein biosynthesis and other anabolic processes; accumulation of metabolic reserves such as glycogen and triacylglycerol (TAG); and inhibition of autophagy and other catabolic pathways, principally through Akt-mediated restraint of AMPK and FOXO programs [191,192].

AMPK, in turn, is the key sensor of ATP deficiency, specifically a shift in the AMP/ADP/ATP ratio toward AMP. AMP is the principal activator of AMPK. Hypoxia-inducible factor-1 (HIF-1) can act upstream of AMPK in settings of hypoxia and inflammatory stress, where NF-κB/MAPK nodes are concurrently engaged [193,194]. AMPK promotes ATP restoration by stimulating lipid breakdown; anaerobic and aerobic metabolism of glycogen and glucose (in part through FOXO activation); autophagy; cellular uptake of glucose and free fatty acids (FFAs); and by limiting anabolism, including via mTOR inhibition [192,193,194,195,196].

The interplay between these MCS directions is complex and extends beyond simple competition. Anabolism is energetically costly and depends on metabolites that are also generated through catabolism, creating coupled feedback rather than a binary switch. AMPK therefore functions not only as a “catabolic driver,” but as a balancer that supports homeostasis and survival under extreme conditions. AMPK plays a crucial role in both insulin-dependent and insulin-independent (exercise-induced) GLUT4 translocation. Moreover, partial AMPK engagement may occur downstream of insulin signaling via PI3K/Akt/HIF-1 coupling, depending on context [193,194,195,196,197,198].

When compensatory MCS capacity is exceeded, IR can develop alongside lipotoxicity-driven mitochondrial dysfunction, with reduced aerobic fatty-acid oxidation and further amplification of lipotoxicity. These changes are commonly accompanied by oxidative stress and activation of NF-κB, MAPKs (JNK, p38), and PKC isoforms, which are mechanistically consistent with inhibitory pressure on insulin signaling [199]. The AMPK-mediated response mitigates mitochondrial stress, promotes mitochondrial biogenesis, facilitates mitophagic clearance of irreversibly damaged mitochondria, enhances glucose uptake, and supports anaerobic glycolysis as an alternative ATP source [200,201,202,203,204]. In aggregate, AMPK activation is generally associated with improved insulin responsiveness, and pharmacologic AMPK activation remains a plausible therapeutic strategy for IR [205,206,207].

Thus, MCS enforces a dynamic balance between mobilization and storage of nutrients and between the generation and consumption of energy in macroergic molecules, chiefly ATP. When substrate load exceeds the compensatory potential of MCS, metabolic distress ensues and MCS signaling becomes dysfunctional [208,209]. In this context, InsRec function is particularly vulnerable, given the largely unique and difficult-to-replace metabolic effects of insulin.

Finally, CS signaling should be viewed as an integrated network rather than as autonomous linear pathways. For example, one study documented persistent alterations in more than 800 phosphosites in hepatocyte signaling pathways in T2DM, including the PI3K/Akt cascade [210]. Phosphorylation is a central regulatory mechanism, but it operates alongside multiple other layers of control (e.g., protein abundance, localization, and post-translational crosstalk).

## 4. Metabolic and Other Effects of the Insulin Receptor and Their Relationship to Cellular and Tissue Stress

### 4.1. Insulin Receptor Signaling Pathways and Their Regulation in Health and Disease

The insulin receptor (InsRec) is a glycosylated, disulfide-linked transmembrane homodimer composed of two repeating ectodomains that bind several (up to four) insulin molecules, a single transmembrane helix, and two intracellular cytoplasmic domains that contain a tyrosine kinase domain (TKD) [211,212]. The InsRec gene undergoes alternative splicing, yielding two isoforms, InsRec-A and InsRec-B, whose functional differences are commonly attributed to distinct affinities for insulin-like growth factors, particularly IGF-2 [213]. The liver is the principal organ responsible for insulin clearance via InsRec-mediated endocytosis, and defects in this process can cause hyperinsulinemia [214].

**Figure 2:**
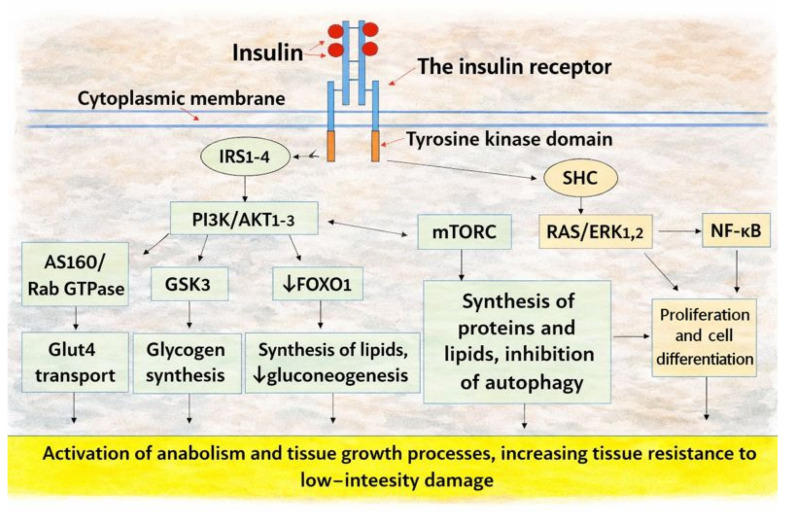
Two principal branches of insulin-receptor signaling (details in the main text).

The binding of insulin induces conformational rearrangement of InsRec followed by TKD autophosphorylation on tyrosine residues and activation of downstream signaling [211,212]. Two principal signaling branches are commonly distinguished (Figure 2): an IRS-dependent route that predominantly mediates metabolic actions, and a SHC-dependent route that engages Ras/RAF/MEK/ERK signaling and is more closely associated with mitogenic and growth-related effects. This division is operational rather than absolute, because the branches are extensively interconnected and share downstream nodes, including PI3K/Akt and PKC-dependent modules [215,216].

A third proposed route involves InsRec nuclear translocation and promoter binding in genes regulating lipid metabolism and protein synthesis [217]. InsRec also regulates additional metabolic mechanisms not shown in Figure 2; for example, insulin inhibits lipolysis by activating PDE3B (reducing cAMP/PKA activity) and by activating PP1/PP2A, which dephosphorylate key lipolytic regulators [14].

**Figure 3:**
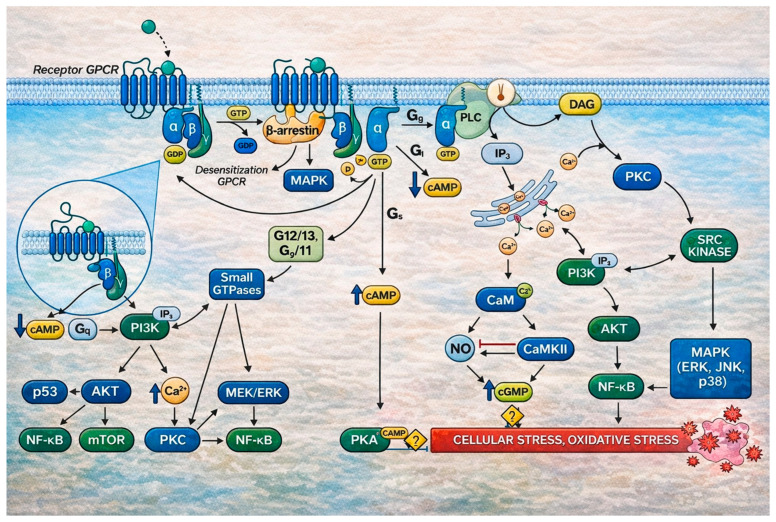
The role of GPCRs in the development of cellular stress. Note. Technical abbreviations are defined at first use. A G-protein-coupled receptor (GPCR) is associated with a heterotrimeric (αβγ) G protein (G). Ligand binding promotes Gα activation and downstream effector engagement; signaling is terminated by Gα GTPase activity and reassociation with Gβγ. GPCR outputs relevant to cellular stress converge on a limited set of canonical modules: (1) PLC-dependent Ca^2+^/DAG signaling (predominantly via Gq/11 and, in part, Gβγ) generates IP3 and DAG, mobilizes ER Ca^2+^, activates PKC, and engages PKC/Src-dependent stress cascades including MAPKs (ERK, JNK, p38) and NF-κB; Ca^2+^-dependent routes additionally involve CaMK. (2) PI3K-centered signaling (“trunk and forks”) can be driven by Gα/PKC, small GTPases, and Gβγ; downstream PI3K/Akt influences cell-cycle control, survival/apoptosis, and DDR-related regulation via FOXO/p53, and interfaces with neuronal survival modules (including Cdk5). (3) Gs–AC–cAMP–PKA versus Gi/o inhibition of AC tunes pleiotropic PKA-dependent programs; reported inflammatory effects are context-dependent across cell types and disease states. (4) Ca^2+^/calmodulin/CaMK signaling interfaces with NO/cGMP regulation, contributing to stress-modulatory effects in excitable tissues. (5) Small GTPases (Ras, Rho, Rab) engaged downstream of G12/13 and Gq support ERK- and PI3K-linked signaling and trafficking control. (6) β-arrestin-dependent signaling can activate MAPKs (notably ERK/JNK) while mediating desensitization and internalization.

As noted above, RTK- and GPCR-mediated signaling shows substantial overlap, underpinning functional coupling between these receptor families (Figure 3) [107]. Many RTKs undergo reciprocal transactivation with GPCRs and converge on shared downstream routes, including PI3K/Akt, ERK, and PKC [218,219]. In the heart, InsRec can form membrane complexes with β2-adrenergic receptors, supporting context-dependent modulation of contractility [220]. Conversely, InsRec crosstalk with angiotensin II receptors (ATR1) has been mechanistically linked to IR-promoting signaling and meta-inflammatory amplification [221]. GPCR control is also central to pancreatic islet hormone secretion, and selected GPCR inputs can support β-cell replication and survival programs [222]. Through the Gs/AC/cAMP/PKA axis, GPCRs can counter insulin actions on glycogenolysis, lipolysis, and gluconeogenesis, whereas Gi inhibits AC and cAMP formation (Figure 3).

Protein tyrosine phosphatases (PTPs) negatively regulate InsRec signaling by antagonizing tyrosine phosphorylation [14,223,224,225,226]. PTP1B is particularly notable: it dephosphorylates InsRec and IRS proteins, is expressed in liver, skeletal muscle, and adipose tissue, and remains a candidate therapeutic target in obesity, metabolic syndrome, and T2DM [227,228,229].

Serine and Ser/Thr kinases provide an additional inhibitory layer for InsRec signaling [230,231]. IRS proteins, Akt kinases, and other nodes implement intrinsic negative feedback that limits signal amplitude and duration [232,233,234]; IRS adaptors can also facilitate InsRec endocytosis through cytoskeletal interactions [230]. Under pro-inflammatory cellular stress, stress-activated kinases—especially JNK—can be recruited to this regulatory layer and contribute to inhibitory phosphorylation of insulin-signaling proteins [232,233,235,236,237,238]. Cytokine-driven JAK/STAT signaling (including IL-6- and leptin-dependent routes) induces SOCS proteins, which bind InsRec phosphotyrosines and inhibit receptor signaling [239,240,241].

Multifunctional PKC isoforms inhibit multiple components of insulin signaling, including InsRec, IRS, and Akt, and can modulate downstream metabolic enzymes such as glycogen synthase (Table 1). RTKs, similar to GPCRs, activate PKC mainly via PLC/IP3/DAG/Ca^2+^ routes and selected PI3K–PKC variants. Some insulin-activated PKCs (e.g., aPKCζ) can act as negative-feedback terminators of insulin signaling [242,243]. Lipid excess promotes intracellular accumulation of DAG and ceramides; ceramides can stimulate aPKCs and inhibit Akt via PP2A, providing a mechanistically plausible route to IR [244]. Although PKC family members share substrate overlap, their actions are typically restricted to local lipid-defined signaling microdomains and depend on co-activation with other kinases [245]. Accordingly, PKC upregulation during pro-inflammatory stress may contribute to IR, while selected PKC-dependent effects may remain supportive for physiological insulin secretion and context-specific metabolic regulation [246,247,248,249,250,251,252,253,254]. PKC also participates broadly in RTK-driven migration/proliferation and in stress-network formation involving MAPKs, STAT3, and NF-κB [255,256,257,258].

In addition, IR can arise from direct counteraction of insulin’s effects at the level of key metabolic enzymes. In hepatocytes, acetyl-CoA produced from excess FAs activates pyruvate carboxylase, a rate-controlling enzyme of gluconeogenesis, and engages other routes that stimulate gluconeogenesis, constituting one mechanism within the Randle cycle [268,269,270,271]. During complete fasting, healthy human livers oxidize roughly 250 g of FAs released from adipose TAG stores per day, generating large amounts of ketone bodies that can substitute for glucose in obligately glycolytic tissues; circulating ketones rise approximately ten-fold [272,273,274,275]. In IR, ketogenesis in hepatocytes may be impaired, diverting FAs toward TAG synthesis, a key mechanism in the development of MASLD/NAFLD [276,277]. Pro-inflammatory cytokines and other CS inducers can modulate these and other metabolic processes and thereby influence insulin’s metabolic actions [278,279,280,281].

Thus, insulin–InsRec signaling is controlled by layered activating and inhibitory feedback. Dysregulated metabolic stress—particularly lipotoxicity—shifts this control toward pro-inflammatory cellular and tissue stress and thereby promotes IR. The next subsection considers specific links between IR and canonical cellular-stress phenomena, including pro-resolving and anti-inflammatory mediators that shape CS trajectories.

### 4.2. Links Between Insulin Resistance and Key Manifestations of Cellular Stress

At the cellular level, IR and stress signaling are linked by reciprocal reinforcement: stress programs attenuate insulin-receptor signaling, whereas impaired insulin action shifts cells toward persistent stress activation and weaker resolution, thereby sustaining meta-inflammation. Table 2 maps these bidirectional links across core cellular-stress (CS) modules—oxidative, mitochondrial, and endoplasmic reticulum (ER) stress; inducible heat-shock protein (HSP) responses; autophagy; inflammasome activation; the DNA-damage response (DDR); and stress-responsive microRNAs (miRNAs), including their intercellular transfer via extracellular vesicles.

First, oxidative stress, mitochondrial dysfunction, and ER stress form a tightly coupled triad that, once amplified, is consistently associated with impaired insulin signaling and with the progression of T2DM and its complications. In most insulin-sensitive tissues, escalation of these modules is therefore more plausibly positioned as a driver and amplifier of IR than as a neutral correlate.

Second, inducible HSP responses represent a protective, systems-level buffering arm of CS: they stabilize the proteome and coordinate cross-module stress control. When HSP capacity is insufficient or exhausted, multiple insulin-dependent cell types become more vulnerable to stress-driven impairment of insulin signaling. Several HSPs (notably HSP70) can be released extracellularly during stress, and circulating increases have been proposed as stress-associated biomarkers in T2DM, with the caveat that their interpretation is context-dependent.

Third, inflammasome activation (particularly NLRP3) typically reflects a shift toward a pro-inflammatory CS trajectory and is mechanistically aligned with IR-promoting cytokine outputs. Autophagy, by contrast, is best framed as a compensatory quality-control program: although it can rise in parallel with inflammatory stress, its net role is often stabilizing because it removes damaged organelles/protein complexes and limits downstream inflammatory amplification.

Finally, stress-inducible miRNAs operate as fine-tuners of the stress proteome and as intercellular signals when exported in extracellular vesicles. Their effects are frequently non-uniform—miRNA actions can be protective or deleterious depending on the specific miRNA species, cell type, and disease stage—yet selected circulating miRNAs remain promising candidates for IR-related biomarker panels.

Table 3 briefly summarizes the principal features of IR interactions with key CS transcription factors, namely NF-κB, p53, AP-1, HIF-1α, HSF-1, NRF2, ATF4, and STAT. Overall, hyperfunction—more precisely, dysfunction—of NF-κB, AP-1, and several STAT family members promotes IR, suggesting that inhibitors of these factors may represent therapeutic targets in T2DM. In contrast, HSF-1, the main inducer of HSPs, likely optimizes CS and reduces IR. The influences of transcription factors central to DDR (p53), metabolic and ER stress (ATF4), and the antioxidant response (NRF2) on IR are context-dependent and sometimes contradictory. This underscores that the problem in IR is not oxidative stress or other CS manifestations per se, but their unbalanced, dysfunctional progression.

It is also of interest to consider pro-resolving and anti-inflammatory factors that affect IR (Table 4). Pro-resolving mediators include anti-inflammatory eicosanoids such as lipoxins, as well as omega-3 FA derivatives—resolvins, protectins, and maresins. All act through GPCRs (FPR2/ALX, GPR18, BLT1, GPR32, GPR37, chemerin1). Their overall effects on IR are protective, and they may be used in pharmaceutical preparations or omega-3-rich foods as adjunctive therapies for the metabolic syndrome and T2DM. Unequivocally anti-inflammatory cytokines—IL-10, IL-35, and IL-38—also show beneficial actions in IR, whereas the effects of the IL-1 receptor antagonist (IL-1RA) are mixed. Conditionally anti-inflammatory cytokines such as IL-11, IL-27, and TGF-β likewise exert context-dependent effects on IR. IL-11 and TGF-β dampen acute inflammation yet promote fibrosis and chronicity.

Taken together, amplification of pro-inflammatory cellular and tissue stress mechanisms interferes with InsRec signaling and promotes IR. These mechanisms should be considered alongside metabolic factors; tissue-specific organokines (myokines, lipokines, hepatokines); canonical counter-regulatory hormones; and heightened autonomic tone, all of which can influence the development of IR.

## 5. Roles of Metabolic Factors in Insulin-Responsive Tissues in the Development of Insulin Resistance and Inflammation

### 5.1. Peroxisome Proliferator-Activated Receptors (PPARs)

Peroxisome proliferator-activated receptors (PPARs) are a family of nuclear receptors that function as ligand-regulated transcription factors controlling the expression of numerous genes. Three isoforms—PPARα, PPARγ, and PPARδ (also termed PPARβ)—are essential for preventing metabolic disorders and their consequences [521]. In addition to regulating transmembrane transport and the metabolism of lipids and glucose, as well as the synthesis of bile acids from cholesterol, PPARs attenuate pathological responses to a range of harmful stimuli, including inflammatory injury [522]. PPARs also regulate cellular proliferation and differentiation, including within the immune system; maintain vascular homeostasis; and are implicated in the pathogenesis of atherosclerosis, cancer, neurodegeneration, and many other diseases [523,524,525,526]. These properties underscore the special role of PPARs in maintaining insulin sensitivity and counteracting insulin resistance (IR). The principal metabolic and anti-inflammatory effects of PPARs are summarized in Table 5.

Thus, the main metabolic and anti-inflammatory effects of PPARs can be described as follows:

- PPARs activate the main intracellular pathways of catabolism and alternative utilization of incoming lipids and glucose, while regulating the formation of lipoproteins and intracellular transport forms of fatty acids (FAs).
- PPAR expression is widespread but is most pronounced in insulin-responsive tissues (skeletal muscle, liver, and adipose tissue).
- PPAR-mediated effects prevent excessive accumulation of glucose, FAs, and other lipid species in blood and within cells, thereby limiting lipotoxicity and glucotoxicity.
- PPARs restrain oxidative stress and other manifestations of pro-inflammatory cellular and tissue stress; at the tissue level, they enhance the anti-inflammatory functions of M2 macrophages.
- PPAR activity lowers atherogenic lipoproteins and raises high-density lipoproteins in circulation, counteracting endothelial dysfunction (endotheliosis), hypertension, and atherosclerosis. (Here and throughout the manuscript, we use “endotheliosis” to denote a low-grade, metabolically driven endothelial stress phenotype within systemic meta-inflammation (i.e., a form of endothelial dysfunction characterized primarily by chronic activation and NO/barrier imbalance rather than overt inflammatory injury). By contrast, “endotheliitis” is reserved for classical endothelial inflammation in focal lesions or systemic hyperinflammation.)
- However, imbalanced expression and activation of different PPAR isoforms can contribute to dysfunctional systems during meta-inflammation and oncogenesis [559].

Overall, PPAR effects stabilize metabolic homeostasis and prevent—or optimize responses to—metabolic stress. PPAR agonists, therefore, have therapeutic potential in type 2 diabetes mellitus (T2DM).

### 5.2. Glucose Transporters

Because glucose cannot diffuse through the lipid bilayer unaided, specific proteins mediate its transport. Three distinct families are recognized: facilitative glucose transporters (GLUT proteins), sodium-dependent glucose symporters mediating secondary active transport (SGLT proteins), and a newer class of glucose uniporters (SWEET proteins) [560,561].

In humans, only one SWEET family member, SWEET1, is expressed. It localizes mainly to the Golgi apparatus and is thought to supply glucose for lactose synthesis in the mammary gland [562].

SGLTs are active, energy-dependent transporters that can move glucose against its concentration gradient at high rates. Their principal roles are intestinal glucose uptake and renal glucose reabsorption (Table 6).

GLUTs mediate glucose transport across membranes down a concentration gradient through conformational cycling. Different GLUT isoforms are expressed in specific tissues, are regulated by hormones and metabolism, and display different kinetic properties. This enables precise control of glucose uptake to meet energetic demands. Based on structure, GLUT1–14 are grouped into three classes: class I (GLUT1–4, GLUT14), class II (GLUT5, GLUT7, GLUT9, GLUT11), and class III (GLUT6, GLUT8, GLUT10, GLUT12, GLUT13). Major GLUT functions and disease associations are summarized in Table 7.

GLUT1–4 are the principal glucose transporters for blood-to-tissue glucose flux, whereas most other GLUT isoforms play auxiliary roles and/or preferentially transport other monosaccharides (e.g., fructose, galactose). These non-glucose substrates may still be relevant to IR pathogenesis because hepatic fructose and galactose can feed gluconeogenic and lipogenic pathways, and because fructose-derived intermediates can intersect with core glycolytic and pentose-phosphate fluxes. GLUT1 and GLUT3 ensure glucose supply to largely insulin-independent tissues, and their expression is regulated by metabolites and by shared stress–metabolic nodes including PI3K/Akt, AMPK, PPARγ, and HIF-1 [584,585,586,587,588,589,590,591,592,593,594]. Notably, GLUT1 expression increases in M1-polarized macrophages, whereas GLUT3 expression rises in M2 macrophages, with prominent intracellular (including endosomal) localization reported for GLUT3 [595].

GLUT2 has low affinity for glucose and mediates bidirectional transport when a sufficient concentration gradient exists. This property is critical for epithelial glucose export (intestine and kidney) and for dynamic hepatic glucose flux—uptake versus release—depending on plasma–hepatocyte gradients. Accordingly, insulin and counter-insular signals in the liver modulate systemic glycemia primarily by regulating hepatic glucose production and utilization rather than by “forcing” glucose entry through high-affinity transport. Hepatocyte GLUT2 expression is regulated by glycemic status and transcriptional control, including HNF1α and FOXA2 [574]. In pancreatic β-cells, GLUT2 and GLUT1 are the major transporters supporting glucose entry and coupling to insulin secretion (Table 7).

As noted above, GLUT4 is the key, largely irreplaceable transporter governing insulin-stimulated glucose uptake in insulin-dependent tissues; its plasma-membrane abundance is directly insulin-regulated except during muscle contraction, where insulin-independent recruitment predominates.

### 5.3. Transport Forms of Fatty Acids (FAs)

Because FAs are hydrophobic, protein binding is required for solubility in aqueous media. In extracellular fluids—especially plasma—free FAs (FFAs) circulate predominantly bound to albumin, which provides multiple high-affinity binding sites [596]. By contrast, transmembrane FA uptake and intracellular trafficking require dedicated transporters and chaperones.

#### 5.3.1. FABPs

Long-chain FAs (LCFAs) are strongly hydrophobic and can become lipotoxic when intracellular handling capacity is exceeded. LCFAs therefore typically remain complexed with fatty-acid-binding proteins (FABPs), a family of small cytosolic lipid chaperones that bind FAs and other hydrophobic ligands. FABPs function less as passive “carriers” and more as trafficking and signaling organizers: they route cargo toward endoplasmic reticulum and mitochondria, facilitate lipid exchange at membrane interfaces, and deliver ligands to enzymes and signaling receptors, including PPARs. Given their tissue-specific expression patterns and functional pleiotropy, FABPs are positioned at the intersection of energy balance, lipid metabolism, and inflammatory signaling, and FABP dysregulation is repeatedly linked to IR and T2DM.

Nine FABP isoforms are recognized in humans (Table 8). FABP1–5, which are prominent in the intestine and insulin-responsive tissues, are the isoforms most consistently implicated in metabolic disease phenotypes, including IR/T2DM and their vascular and hepatic complications. FABP6 primarily handles bile acids and is often reported as metabolically protective (while exhibiting distinct associations in oncology). FABP7–8 are more closely tied to neural physiology, whereas FABP9 is predominantly expressed in the reproductive system.

#### 5.3.2. Carnitine

Carnitine is essential for mitochondrial FA utilization and energy production, particularly in the heart and skeletal muscle [630]. Its core metabolic role is to enable mitochondrial import of long-chain acyl groups via the carnitine shuttle, thereby supporting β-oxidation and ATP generation. By facilitating FA use, carnitine may support fat mobilization and weight control; however, weight-loss effects are not uniform and should not be stated as intrinsic outcomes of carnitine biology. Carnitine is synthesized mainly in the liver and kidneys (with additional synthesis reported in other tissues) from lysine and methionine when dietary intake is insufficient. In clinical and experimental contexts, supplementation has been explored as a modifier of sarcopenia, obesity-associated metabolic dysregulation, IR, and low-grade inflammation, but effects appear heterogeneous and context-dependent [631]. Carnitine has been reported to attenuate mitochondria-dependent apoptosis, and FA–carnitine flux has been linked to mitochondrial remodeling/biogenesis signaling; these observations remain mechanistically plausible but should be framed as associative/experimental rather than universal [632]. Primary carnitine deficiency is associated with fatty liver and low hepatic and circulating carnitine levels [633].

Excess LCFA influx can increase intracellular long-chain acylcarnitine burden in mitochondria and other compartments, a state often interpreted as a marker of incomplete FA oxidation and mitochondrial overload rather than a single causal toxin; such accumulation is linked to pro-inflammatory stress signaling and may contribute to IR in obesity/T2DM settings [634,635,636]. Incomplete FA oxidation is associated with accumulation of medium- and long-chain acylcarnitines (approximately C4–C22) alongside relative depletion of free carnitine. These acylcarnitines can be released and detected in blood and urine, where they are widely used as biomarkers of altered lipid oxidation in obesity and T2DM [637].

#### 5.3.3. FATPs

Long-chain FA transport across the plasma membrane is mediated by fatty-acid transport proteins (FATPs; Table 9). FATPs are broadly expressed in LCFA-utilizing tissues and facilitate LCFA uptake coupled to intracellular activation (acyl-CoA formation), thereby linking membrane transport with metabolic routing and regulatory-lipid generation. FAT/CD36 functions both as an LCFA transporter and as a signaling receptor. Across tissues, FATP1–5 and CD36 are the transporters most consistently discussed in relation to IR/T2DM phenotypes, whereas FATP6 is primarily emphasized in cardiometabolic contexts; importantly, reported effects are strongly tissue- and stage-dependent and often reflect broader lipotoxic load and inflammatory signaling rather than a single “transporter-only” mechanism.

### 5.4. Lipokines, Hepatokines, and Myokines

**Figure 4:**
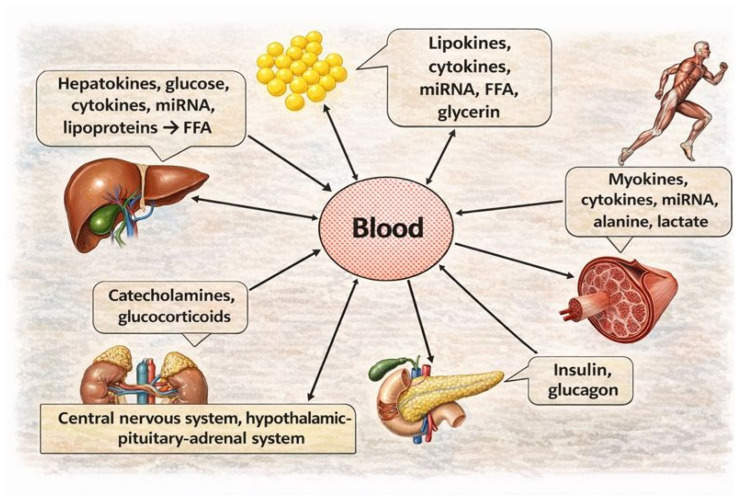
Mechanisms linking insulin-responsive metabolic tissues. Note. FFAs, free fatty acids; miRNA, microRNA within extracellular vesicles.

Insulin-responsive organs form an integrated regulatory system that maintains whole-body metabolic homeostasis (Figure 4). This coordination is mediated by (i) inter-organ substrate cycles, (ii) endocrine/paracrine signaling by adipose-, liver-, and muscle-derived factors (lipokines/adipokines, hepatokines, myokines), (iii) canonical pancreatic hormones, and (iv) extracellular-vesicle transfer of miRNAs and other regulatory cargo. Together, these mechanisms establish horizontal communication among adipose tissue, liver, skeletal muscle, and pancreatic β-cells. Vertical integration is provided by CNS control, pituitary–adrenal axes, and autonomic input, while the immune system, microbiome, and intestinal barrier contribute additional regulatory layers. Failure of this multi-level network promotes persistent meta-inflammation and dysregulated nutrient handling, thereby facilitating IR, the metabolic syndrome, and progression to T2DM [685].

Adipocyte-, hepatocyte-, and muscle-derived histohormones act through autocrine, paracrine, and endocrine routes. Some mediators are relatively organ-enriched, whereas others (e.g., FGF21) are liver-dominant but not liver-exclusive. Adipocytes, hepatocytes, and myocytes can also produce classically pro-inflammatory cytokines, particularly under meta-inflammatory conditions, thereby linking metabolic regulation with immune activation [686,687,688,689,690].

Lipokines/adipokines can be grouped by their dominant metabolic and inflammatory signatures. Leptin, visfatin, and chemerin are frequently described as metabolically compensatory (often correlating with improved insulin action in specific settings) yet capable of pro-inflammatory signaling, whereas resistin, PAI-1, RBP4, WNT5A, and ANGPTL2 are more consistently linked to both IR-promoting actions and inflammatory amplification. In contrast, adiponectin, SFRP5, apelin, vaspin, and omentin-1 are generally positioned as insulin-sensitizing mediators with anti-inflammatory or pro-resolving tendencies. In obesity and T2DM, the circulating adipokine milieu typically shifts toward a higher pro-inflammatory burden and a relative loss of insulin-sensitizing signals (Table 10).

Similarly, hepatokines show divergent effects (Table 11). Fetuin-A, ANGPTL2, LBP, selenoprotein P, and LECT2 promote IR and inflammation, whereas hepatokines improving insulin sensitivity change variably.

Myokines are induced by muscle work and generally enhance insulin sensitivity and optimize metabolic stress, partly via AMPK (Table 12). These include irisin, SPARC, BAIBA, and BDNF. Muscle also releases IL-6 and IL-13, which increase hepatocyte insulin sensitivity. In obesity and T2DM, levels of anti-IR myokines (e.g., irisin, BDNF, GDF11, IL-13, IL-15) decline. Exercise, therefore, improves insulin sensitivity and metabolic stress responses.

### 5.5. Links Between IR and Intestinal Function and the Gut Microbiota

The gut microbiota is increasingly regarded as an important modulator of insulin resistance, acting through metabolite profiles that shape glucose–lipid homeostasis and through barrier dysfunction that can amplify meta-inflammation [772]. Dysbiosis has been associated with morbid obesity, metabolic syndrome, and T2DM, plausibly through combined effects on energy harvest, immunometabolic signaling, and low-grade inflammation [773]. In experimental models, metagenomic and biochemical studies indicate that obesity and IR shift microbiome composition and metabolic capacity; notably, microbiota from obese donors can confer a greater fat-gain phenotype when transferred to germ-free mice, supporting a transmissible component of the metabolic state [774].

Excess monosaccharides reaching the distal gut may contribute to obesity and promote immune activation, facilitating pro-inflammatory cytokine responses, metabolic syndrome, and IR [775]. In parallel, specific taxonomic patterns have been linked to altered fecal carbohydrate profiles and metabolic endpoints; for example, *Lachnospiraceae* members (including *Dorea* and *Blautia*) have been associated with higher fecal monosaccharides and positive correlations with IR [776]. In human cohorts, metagenomic analyses of T2DM typically report moderate dysbiosis, a reduction in several butyrate-producing taxa, and an increase in opportunistic pathogens [777,778,779]. Reported decreases include butyrate-associated genera/species such as *Eubacterium rectale*, *Faecalibacterium prausnitzii*, *Roseburia intestinalis*, and *Roseburia inulinivorans*, whereas taxa such as *Ruminococcus*, *Fusobacterium*, and *Blautia* have been reported to correlate positively with T2DM, and *Bifidobacterium*, *Bacteroides*, *Faecalibacterium*, *Akkermansia,* and *Roseburia* negatively (noting that directionality may vary across cohorts and analytical pipelines) [777,778,779].

A major functional axis linking microbiota to metabolic regulation is the production of short-chain fatty acids (SCFAs)—acetate, propionate, butyrate, and lactate—via anaerobic fermentation of dietary fiber. SCFAs can influence metabolic homeostasis by stimulating intestinal histohormones (e.g., GLP-1 and peptide YY) and by acting directly on enterocytes, adipocytes, immunocytes, and pancreatic β-cells through GPCRs, including GPR41 (FFAR3), GPR43 (FFAR2), and GPR109A (HCA2) [780,781,782]. These receptors are also termed free-fatty-acid receptors (FFARs) [783,784] and have attracted interest as potential therapeutic targets for metabolic disease and inflammation control [781]. Four FFAR isoforms are commonly distinguished (FFAR1–4). For clarity, SCFA sensing is primarily attributed to FFAR2 and FFAR3, whereas long-chain fatty acids preferentially activate FFAR1 (GPR40) and FFAR4 (GPR120) [782]. FFAR1 can augment glucose-stimulated insulin secretion in β-cells, while FFAR4—expressed across multiple tissues—modulates histohormone production and may exert anti-inflammatory effects in macrophage populations, including Kupffer cells. Consistent with this, reduced FFAR4 function has been linked to increased IR and obesity risk in several insulin-sensitive tissues, and omega-3 polyunsaturated fatty acids are recognized FFAR4 agonists [785,786,787,788].

Dietary patterns provide a clinically relevant lever for these pathways. Individuals consuming plant-based, high-fiber diets often show enrichment of fiber-fermenting, SCFA-producing bacteria, and these shifts have been reported to correlate with reduced systemic meta-inflammation, including lower circulating pro-inflammatory cytokine signals [789].

Beyond metabolites, dysbiosis may compromise intestinal barrier integrity—particularly the mucus layer and epithelial tight junctions—thereby increasing permeability and facilitating systemic exposure to PAMPs and other bacterial products, a mechanism frequently invoked to explain low-grade inflammation in T2DM [779]. Experimental data support bidirectional coupling between insulin signaling and barrier function: in mice, the InsRec antagonist S961 can increase gut permeability independent of obesity, global IR, and hyperglycemia [790]. Conversely, metabolic dysregulation (including impaired AMPK activity and altered GLUT2-related pathways) has been linked to reduced expression of tight-junction proteins and barrier weakening [791,792]. In meta-inflammatory states, circulating levels of LPS—the prototypic PAMP—may rise several-fold due to barrier dysfunction, while remaining substantially lower (by approximately one to two orders of magnitude) than concentrations observed in bacterial sepsis [793].

## 6. Interrelationship Between Insulin Resistance and Inflammation

### 6.1. Diversity of Tissue Pro-Inflammatory Stress Forms and Their Relationships with Metabolic Pathways

A consolidated view of inflammation as a typical protective reaction aimed at localizing and eliminating a damaging factor, followed by regeneration or repair (scarring) of injured tissues, had finally taken shape by the early twentieth century through the works of R. Virchow, J. Cohnheim, I. Mechnikov, P. Ehrlich, and other distinguished investigators [794]. The attribute of canonical (classical) inflammation is the microvascular reaction, which ensures exudation and the migration of leukocytes into the site of tissue damage. Taken together, these processes determine the five classical external signs of inflammation: rubor, tumor, calor, dolor, and functio laesa.

Quasi-inflammatory processes—such as phagocyte-mediated encapsulation of pathogens and manifestations of the systemic inflammatory response (changes in the volume and concentration of hemolymph cells and stress proteins)—are characteristic of invertebrates; however, the full set of canonical inflammatory features, including the exudative–vascular response, appears to be typical only of vertebrates [795,796,797,798,799,800,801,802]. Lower vertebrates are capable of productive (proliferative-cellular) and exudative inflammation, whereas higher vertebrates additionally demonstrate exudative–destructive caseous inflammation (reptiles and birds) and purulent inflammation (mammals) [795,803]. Mammals are also characterized by the phenomenon of systemic hyperinflammation, understood not as isolated manifestations of a systemic inflammatory response but as a typical pathological process that includes microthrombosis, intravascular activation and adhesion of leukocytes to the endothelium, systemic morphofunctional transformation of postcapillary endothelial cells, and critical manifestations of microcirculatory disorders [795,804,805,806,807,808,809,810,811,812,813]. It should be emphasized that the evolution of genetically determined inflammatory programs is closely linked to the progressive complexity not only of the immune system but also of the central nervous system (CNS), the cardiovascular system, and other organ systems [795,814,815].

Many inflammatory mechanisms are themselves capable of injuring host tissues—for example, free radicals, various hydrolases, cationic proteins, and disturbances of oxygen transport resulting from “inflammatory microcirculation.” Consequently, an anti-inflammatory resolving response forms during inflammation, which limits both the intensity and the duration of the inflammatory reaction [816,817]. When the balance between pro-inflammatory and anti-inflammatory mechanisms is disturbed, inflammation may harm the host organism, which determines the rationale for anti-inflammatory therapy in such settings [818,819].

It has now become evident that pro-inflammatory mechanisms are also engaged by stimuli that are sub-threshold for canonical inflammation, beginning with physiological and extreme-physiological processes and, under pathological conditions, within tumor growth zones and in various variants of low-grade inflammation (para-inflammation) that underlie many chronic diseases [820,821,822,823,824]. Causes of systemic low-grade inflammation may include tissue aging (inflamm-aging) [825,826]; metabolic dysfunctions (meta-inflammation) [827,828]; and low-intensity injurious infectious factors characteristic, for example, of slow viral infections and certain variants of Long COVID [829]. Chronic CNS stress and depression may also be associated with increased pro-inflammatory activity of neurotransmitters and activation of microglia in specific brain regions, which can lead to low-intensity neuroinflammation and, with involvement of the CNS–gut–cardiovascular axis, to systemic low-grade inflammation [108,109,830].

The pathological trajectory “morbid obesity → metabolic syndrome → type 2 diabetes mellitus (T2DM)” is most directly associated with meta-inflammation driven by the damaging effects of glucotoxicity and lipotoxicity [831,832]. Nevertheless, other low-grade inflammatory contexts—including inflamm-aging and persistent low-intensity infectious or neuropsychic stressors—may modulate the magnitude and dynamics of meta-inflammation, even if they are not primary drivers in most cases [833,834,835,836,837,838,839]. Conversely, by feedback mechanisms, IR can reinforce low-grade inflammatory tone and thereby worsen the overall inflammatory trajectory [840].

**Figure 5:**
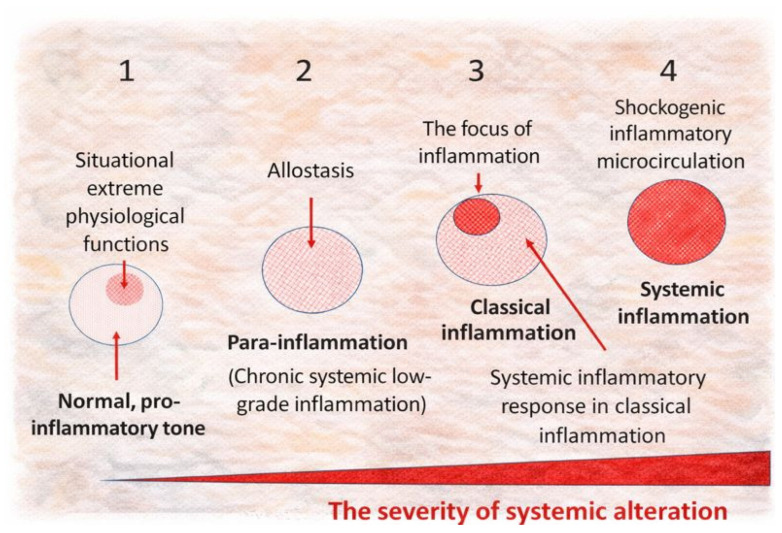
Variants of tissue pro-inflammatory stress. Note. 1—Physiological variants of tissue stress; 2—Non-classical low-grade inflammation (para-inflammation), which at the systemic level may manifest as stably altered homeostasis (allostasis); 3—Classical inflammation (the organism’s response to a significant local injury) is characterized by the presence of its attribute—a focus of inflammation—and, in some cases, a systemic inflammatory response aimed at resourcing the inflammatory focus; 4—Life-critical systemic inflammation, whose key phenomenon is a systemic microvascular response comparable in intensity to the local response within a classical inflammatory focus.

Accordingly, depending on the intensity, context, and spread of pro-inflammatory mechanisms, one can schematically depict a continuum of tissue stress—from basal pro-inflammatory tissue tone and time-limited extreme-physiological states to life-critical systemic hyperinflammation (Figure 5). This schematic is intended as a heuristic rather than a strict linear sequence, because transitions can be discontinuous, tissue-specific, and shaped by feedback loops that blur boundaries between “tone,” para-inflammation, classical inflammation, and systemic microvascular involvement. These non-linearities are addressed below.

Overall, a large number of disorders—previously not considered inflammatory—are now linked to local and systemic phenomena of low-grade inflammation. These include osteoarthritis (arthrosis), various nephrotic entities, many cardiovascular diseases, and several psychiatric and neurodegenerative disorders [841,842,843,844,845,846,847,848,849,850,851,852,853,854,855,856].

Mechanisms of cellular and tissue pro-inflammatory stress can support the tumor microenvironment, which, beyond malignant cells, includes tumor-associated macrophages and blood vessels [857]. However, we consider it more appropriate not to classify the stress state within tumor tissue itself as inflammation, since cancer represents a parasitic anti-system with respect to the organism rather than a programmatic host response to injury [24]. At the same time, tumor initiation and progression may be bidirectionally coupled to inflammation in peritumoral tissues and to systemic low-grade inflammation [858,859,860,861]. In view of this, it appears logical that patients with T2DM have higher risks of developing cancer—particularly of the pancreas, thyroid, urinary bladder, kidney, breast, colon, and liver—than the general population [862,863,864]. The mechanisms of this association are multifactorial; nonetheless, meta-inflammation, hyperinsulinemia, and increased bioavailability of IGF-1 likely contribute materially to tumor initiation and progression in patients with IR. Conversely, evidence from a systematic review and meta-analysis indicates that patients with an oncologic diagnosis frequently exhibit pronounced IR [865]. The presence of IR and signs of systemic low-grade inflammation are often associated with adverse prognosis in cancer [866].

According to several authors, stromal macrophages play a key role in the development of para-inflammation (low-grade inflammation), as they increase their pro-inflammatory activity and enter networked interactions with other resident cells and with leukocytes that migrate—albeit moderately—into the para-inflammatory zone [867,868,869,870,871]. A gradual replacement of macrophages of embryonic origin (from the yolk sac or fetal-liver progenitors) by macrophages of monocytic origin ensues. Simultaneously, macrophage differentiation shifts from the M2 pole (pro-resolving macrophages) toward the pro-inflammatory M1 pole (metabolically activated macrophages in meta-inflammation). For the pathogenesis of systemic low-grade inflammation, pro-inflammatory transformation of the endothelium (endotheliosis) acquires particular importance, facilitating atherosclerosis, hypertension, thrombophilia, and microangiopathies [872,873,874,875,876,877]. It is reasonable to distinguish sluggish endotheliosis from endotheliitis in a classical inflammatory focus and from the more critical microcirculatory disturbances characteristic of systemic hyperinflammation [24].

**Figure 6:**
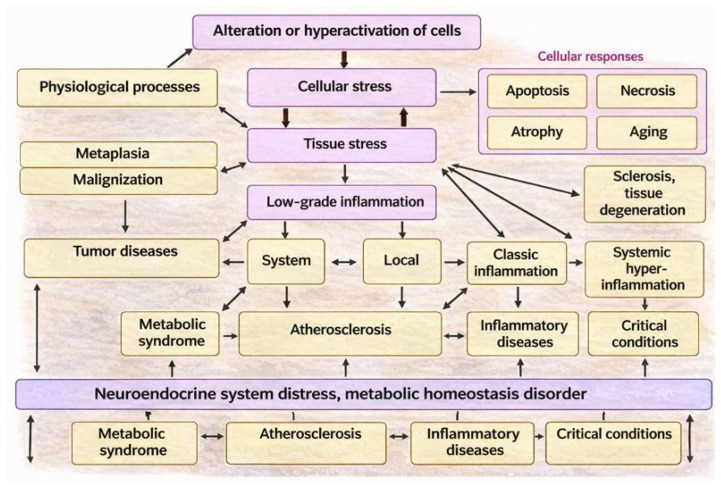
Interrelation of the main general pathological processes on the basis of common mechanisms of cellular and tissue pro-inflammatory stress.

From the standpoint of general pathology, theoretical models of general pathological processes represent universal pathogenetic platforms for diverse diseases [878]. In turn, universal mechanisms of cellular and tissue stress constitute a common platform not only for various general pathological processes but also for many physiological processes (Figure 6). This enables the identification of shared mechanisms of pathogenesis—and, correspondingly, pathogenetic therapies—across conditions of differing nature, ultimately advancing toward an integrative theory of the emergence and interrelation of pathological processes. For example, atherosclerosis develops as a result of local intensification of endotheliosis in specific segments of large arteries, but at the stage of an unstable atherosclerotic plaque, acquires many features of classical productive inflammation [879]. Diabetic kidney disease, which begins as an organ-specific variant of low-grade inflammation, may transform into classical exudative-fibrinous or productive inflammation [880]. Progression of steatohepatosis leads to steatohepatitis with characteristic morphological signs of productive inflammation, namely areas of pronounced leukocyte infiltration [881,882,883]. Sepsis is characterized by failure of the barrier function of the canonical inflammatory focus; however, fulminant shock-inducing forms of systemic hyperinflammation may also arise without prior formation of a classical inflammatory focus [884].

Another facet of an integrative approach is the cross-organ coupling of pathology through systemic regulatory and inflammatory states. Associations have been shown between liver fibrosis, the presence and severity of MASLD/NAFLD, and diabetic kidney disease in patients with T2DM [885,886]. Conversely, chronic kidney failure can promote MASLD/NAFLD progression [887]. Notably, the development of T2DM and liver fibrosis is facilitated by metabolic endotoxemia—a low-grade inflammatory state linked to dysbiosis and increased intestinal permeability with consequent elevation of circulating endotoxin [888]. Links are also evident between T2DM and atherosclerosis, myocardial fibrosis in diabetic cardiomyopathy, neurodegenerative diseases, chronic obstructive pulmonary disease, and pneumosclerosis [182,183,184,185,186,187,188,189,190,191,192,193,194,195,196,197,198,199,200,201,202,203,204,205,206,207,208,209,210,211,212,213,214,215,216,217,218,219,220,221,222,223,224,225,226,227,228,229,230,231,232,233,234,235,236,237,238,239,240,241,242,243,244,245,246,247,248,249,250,251,252,253,254,255,256,257,258,259,260,261,262,263,264,265,266,267,268,269,270,271,272,273,274,275,276,277,278,279,280,281,282,283,284,285,286,287,288,289,290,291,292,293,294,295,296,297,298,299,300,301,302,303,304,305,306,307,308,309,310,311,312,313,314,315,316,317,318,319,320,321,322,323,324,325,326,327,328,329,330,331,332,333,334,335,336,337,338,339,340,341,342,343,344,345,346,347,348,349,350,351,352,353,354,355,356,357,358,359,360,361,362,363,364,365,366,367,368,369,370,371,372,373,374,375,376,377,378,379,380,381,382,383,384,385,386,387,388,389,390,391,392,393,394,395,396,397,398,399,400,401,402,403,404,405,406,407,408,409,410,411,412,413,414,415,416,417,418,419,420,421,422,423,424,425,426,427,428,429,430,431,432,433,434,435,436,437,438,439,440,441,442,443,444,445,446,447,448,449,450,451,452,453,454,455,456,457,458,459,460,461,462,463,464,465,466,467,468,469,470,471,472,473,474,475,476,477,478,479,480,481,482,483,484,485,486,487,488,489,490,491,492,493,494,495,496,497,498,499,500,501,502,503,504,505,506,507,508,509,510,511,512,513,514,515,516,517,518,519,520,521,522,523,524,525,526,527,528,529,530,531,532,533,534,535,536,537,538,539,540,541,542,543,544,545,546,547,548,549,550,551,552,553,554,555,556,557,558,559,560,561,562,563,564,565,566,567,568,569,570,571,572,573,574,575,576,577,578,579,580,581,582,583,584,585,586,587,588,589,590,591,592,593,594,595,596,597,598,599,600,601,602,603,604,605,606,607,608,609,610,611,612,613,614,615,616,617,618,619,620,621,622,623,624,625,626,627,628,629,630,631,632,633,634,635,636,637,638,639,640,641,642,643,644,645,646,647,648,649,650,651,652,653,654,655,656,657,658,659,660,661,662,663,664,665,666,667,668,669,670,671,672,673,674,675,676,677,678,679,680,681,682,683,684,685,686,687,688,689,690,691,692,693,694,695,696,697,698,699,700,701,702,703,704,705,706,707,708,709,710,711,712,713,714,715,716,717,718,719,720,721,722,723,724,725,726,727,728,729,730,731,732,733,734,735,736,737,738,739,740,741,742,743,744,745,746,747,748,749,750,751,752,753,754,755,756,757,758,759,760,761,762,763,764,765,766,767,768,769,770,771,772,773,774,775,776,777,778,779,780,781,782,783,784,785,786,787,788,789,790,791,792,793,794,795,796,797,798,799,800,801,802,803,804,805,806,807,808,809,810,811,812,813,814,815,816,817,818,819,820,821,822,823,824,825,826,827,828,829,830,831,832,833,834,835,836,837,838,839,840,841,842,843,844,845,846,847,848,849,850,851,852,853,854,855,856,857,858,859,860,861,862,863,864,865,866,867,868,869,870,871,872,873,874,875,876,877,878,879,880,881,882,883,884,885,886,887,888,889,890,891]. Across these entities, fibrogenesis is closely related to IR and to local and systemic phenomena of low-grade inflammation [892,893,894,895,896,897]. Over time, chronic fibrosis drives adverse tissue remodeling and functional impairment. Persistent inflammation and fibrosis, therefore, represent shared, high-burden disease axes across organ systems and account for an increasing share of global morbidity and mortality [898,899].

A characteristic feature of T2DM is dysfunction of the CNS–gut axis, which contributes not only to worsening hyperglycemia but also to CNS remodeling (including astrogliosis) and cognitive impairment [900,901,902,903]. In the pathogenesis of T2DM, mechanisms of distinct low-grade inflammatory variants—primarily inflamm-aging and meta-inflammation—are often convergent and mutually reinforcing, resulting in a composite chronic pathological state rather than separable processes [904,905,906,907,908,909,910,911].

At the same time, it would be incorrect to underestimate metabolic and neuroendocrine mechanisms in IR pathogenesis that are not reducible to inflammation. First, the impact of pro-inflammatory factors on IR is most clearly demonstrated within meta-inflammation, but it is not necessarily dominant in other low-grade inflammatory contexts. Second, as noted by Rosen and Kajimura, an increasing body of physiological, genetic, and pharmacological evidence suggests that the paradigm of adipose-tissue inflammation may not fully capture what occurs in human obesity and T2DM [912]. Moreover, inflammation is a genetically determined protective program which—even when dysregulated—retains adaptive components. Accordingly, “anti-inflammatory” directionality is not automatically beneficial in all contexts. For example, a drift of stromal macrophages from the pro-inflammatory M1 pole toward the conditionally anti-inflammatory/pro-resolving M2 pole is not universally favorable. A typical complication of chronic inflammation—fibrosis of internal organs—is preferentially linked to M2-associated programs, as these cells produce TGF-β and activate classical (TGF-β/Smad2/3) and non-classical (PI3K, Rho, TRAF, Ras) profibrotic pathways in fibroblasts and myofibroblasts; via Wnt/β-catenin they can promote differentiation of mesenchymal stem cells into myofibroblasts, and direct transdifferentiation of M2 macrophages into myofibroblasts has also been proposed [913,914,915,916]. Fibrosis—excessive and inappropriate extracellular-matrix deposition—is a typical complication of T2DM, affecting kidneys, heart, liver, lungs, and eyes [917,918,919,920,921,922].

Metabolic dysfunction in IR may not only be pathological but also partly adaptive. There is substantial evidence that hepatic triacylglycerol (TAG) accumulation can protect against fatty-acid-induced lipotoxicity by diverting excess saturated fatty acids into TAG, thereby reducing their toxic intracellular effects in hepatocytes [923,924]. The IR phenotype itself may likewise include adaptive components; for instance, IR can limit TAG synthesis in large, fat-laden adipocytes, which—as noted above—exhibit high degrees of insulin unresponsiveness.

Finally, IR mechanisms are integrated not only with pro-inflammatory tissue stress but also with additional, partially independent drivers of IR-associated pathology. Numerous human and animal studies show that severe IR and hyperinsulinemia can arise in the absence of elevated arterial blood pressure [925]. Other mechanisms—such as physical compression of the kidneys, activation of the renin–angiotensin–aldosterone system, hyperleptinemia, stimulation of hypothalamic melanocortin pathways, and activation of the sympathetic nervous system—appear to play comparably important roles in initiating hypertension in obese individuals with metabolic syndrome. Nevertheless, the metabolic consequences of IR, including hyperglycemia and dyslipidemia, can interact synergistically with these mechanisms to worsen vascular risk [925].

Thus, the pathogenesis of IR and T2DM reflects interactions among multiple, partly opposing processes. Their appraisal should therefore consider general pathological frameworks, more specific mechanistic patterns, and an individualized assessment of how these mechanisms are realized in a given patient.

### 6.2. Characteristics of Meta-Inflammation, Its Phenomena, and Their Links to IR

Meta-inflammation has several defining characteristics. First, it is predominantly driven by metabolic “alteration” factors (glucotoxicity and lipotoxicity), although this does not exclude contributory inputs from low-level circulating PAMPs—arising, for example, from increased intestinal permeability—as well as DAMPs released during regulated or necrotic cell death, and, in selected settings, hypoxia or other forms of low-intensity tissue injury.

Second, meta-inflammation has both local and systemic manifestations. The process plausibly begins locally within insulin-sensitive metabolic tissues (primarily liver and adipose depots) and can subsequently extend to additional organs. We propose that a systemic variant of meta-inflammation should be recognized when the vascular endothelium is broadly engaged—together with vascular-resident macrophage populations (including Kupffer cells in hepatic sinusoids)—and when reproducible laboratory evidence of a systemic inflammatory response is present.

Third, meta-inflammation shares key features with para-inflammation and differs from classical inflammation by the absence of a delimited inflammatory focus with barrier function and by the preferential involvement of resident stromal cells, with emphasis on macrophage-driven remodeling rather than on massive leukocyte influx. Moreover, whereas canonical inflammation is a protective program aimed at localization and elimination of a damaging factor, there is no clear basis to assume that meta-inflammation “eliminates” the metabolic injury imposed by glucotoxicity and lipotoxicity. Rather, meta-inflammation is better viewed as an aberrant, metabolically triggered inflammatory program that nevertheless retains certain compensatory and homeostatic components.

Fourth, meta-inflammation is commonly described as low-grade inflammation. However, as discussed above, locally intensified meta-inflammation may acquire features resembling a classical inflammatory focus. In addition, there exists another para-inflammatory variant—systemic hyperinflammation—which can also become chronic (e.g., in systemic autoimmune diseases and end-stage renal failure) [926]. This state is marked by more pronounced systemic-inflammatory-response features than in canonical and low-grade inflammation, including cytokine elevations by orders of magnitude, coagulopathy with systemic microthrombosis, systemic tissue injury, and activation of the hypothalamic–pituitary–adrenal distress axis, and therefore requires integrated criteria for differential diagnosis [926]. From this perspective, we agree that characterizing meta-inflammation solely by borderline or elevated CRP together with routine laboratory tests is insufficient [927]. In our prior work, we operationalized this distinction by measuring plasma CRP, IL-6, IL-8, TNF-α, D-dimer, troponin I, and cortisol in patients with prediabetes (n = 26) and T2DM (n = 63). Using these criteria, systemic meta-inflammation was identified in 57.7% and 74.6% of patients, respectively; within these, 7.7% and 19% met criteria for hyperinflammation, whereas the remainder conformed to low-grade inflammation [928]. These observations suggest that systemic meta-inflammation is more heterogeneous than is often assumed and may be more appropriately framed as a complication that emerges along the IR/T2DM trajectory, rather than as an invariant attribute of IR. Nonetheless, low-grade inflammation remains the dominant phenotype of meta-inflammation in most settings.

Fifth, meta-inflammation is a chronic process characterized by relatively stable tissue-level changes and by persistent shifts in homeostasis (allostasis) at both local and systemic levels. In this context, it is arguably imprecise to refer to “acute” low-grade inflammation; when pro-inflammatory stress mechanisms are engaged transiently and resolve rapidly, it is more accurate to describe such states as time-limited extreme-physiological stress responses with adaptive intent, rather than as acute manifestations of para-inflammation.

#### 6.2.1. Stromal Macrophages

Stromal macrophages occupy a central position in meta-inflammation, establishing networked interactions with resident parenchymal and stromal cells through secreted mediators of a pro-inflammatory (or stress-adaptive) phenotype. Macrophages can be tuned into multiple specialized states by microenvironmental cues. For conceptual orientation, two principal polarization poles are commonly invoked. Classically activated M1 macrophages are characterized by high NF-κB/iNOS tone, robust production of TNF-α, IL-1β, IL-6, IL-12, and chemokines (including CCL2/MCP-1), and a metabolic program that relies largely on glycolysis to meet energetic demands. Conversely, alternatively activated M2 macrophages generally display lower overt pro-inflammatory stress signatures, can restrain inflammation via IL-10 and IL-1RA (while still producing selected mediators such as IL-6 in a context-dependent manner), and preferentially generate ATP through fatty-acid β-oxidation and oxidative phosphorylation. In acute inflammation, M2-skewed programs contribute to resolution and tissue repair; in chronic settings, they can promote remodeling and sclerosis, most notably fibrosis. In vivo, however, macrophage phenotypes are best viewed as a continuum: numerous intermediate and hybrid states arise across inflammatory contexts, yielding overlapping immunophenotypes rather than discrete M1/M2 categories [24,879,929,930,931].

In insulin resistance, several groups distinguish adipose-tissue macrophage (ATM) subsets such as metabolically activated macrophages (MMe) and/or lipid-associated macrophages (LAM), induced by high local concentrations of FFAs, insulin, glucose, and oxidized LDL (oxLDL) [932,933,934]. In obesity, MMe/LAM can engulf and store lipids; in contrast to atherosclerotic foam cells, these cells preferentially accumulate TAG rather than cholesterol, with TAG handling itself contributing to cellular stress signaling. These macrophages exhibit partial motility and can form crown-like structures around necrotic hypertrophied adipocytes. Importantly, their role in IR is not unidirectional: their pro-inflammatory outputs can impair adipocyte insulin sensitivity, yet their clearance of dead cells and debris can limit tissue-level inflammatory amplification during adipose remodeling and hepatic steatosis.

A common trajectory during progression of meta-inflammation—particularly in adverse T2DM dynamics—is increased macrophage abundance within metabolic tissues and a drift toward M1-like programs, including via recruitment of circulating monocytes and local differentiation, thereby reinforcing IR [935,936,937,938,939,940,941]. The functional repertoire of ATMs is broad and includes proliferation during adipose remodeling, regulation of fibrotic responses, antigen presentation to T cells, production of metabolic enzymes and mediators that shape stromal communication, modulation of adipocyte insulin sensitivity, and control of adipocyte progenitor activation. IR and obesity bias ATMs toward a pro-inflammatory direction primarily through metabolic cues, including elevated FFAs acting via CD36 and TLR2/4, and high glucose effects engaging ROCK/MAPK-linked stress signaling [942,943]. This shift is further promoted—especially in visceral depots—by accumulation of CD8^+^ Tc1 and CD4^+^ Th1 cells (IFN-γ), by Th17 enrichment, and by a relative decline in regulatory T cells (Tregs) [944,945,946,947,948,949,950]. Lifestyle interventions (exercise, diet, and broader behavioral factors) have been reported to stabilize meta-inflammation and, in some contexts, to partially reverse its systemic manifestations [951,952,953,954,955,956].

Kupffer cells are key macrophage populations shaping hepatic meta-inflammation and are mechanistically linked to IR [957,958,959,960]. However, hepatic inflammation driven by Kupffer-cell activation does not uniformly translate into systemic IR across obesity models [961]. In IR, Kupffer cells sit within a dense interaction network involving hepatocytes, stellate cells, endothelial cells, and recruited immune cells, thereby influencing the coupled trajectories of steatosis, inflammatory amplification, and fibrogenesis [962,963,964,965,966].

#### 6.2.2. Cellular Necrosis in Meta-Inflammation

Maintenance of tissue homeostasis critically depends on efficient efferocytosis—the clearance of apoptotic corpses by stromal macrophages, a function classically enriched in M2-skewed programs. When efferocytosis is impaired, and adipocyte apoptosis increases in morbid obesity, metabolic syndrome, and T2DM, apoptotic bodies undergo secondary necrosis, releasing DAMPs that amplify local inflammation and reinforce a self-sustaining loop between adipose remodeling, immune activation, and insulin resistance [967,968,969,970].

Other forms of programmed cell death also sustain meta-inflammation. Pyroptosis plays a special role and is linked to NLRP3 inflammasome hyperactivity in insulin-dependent tissues, since NLRP3 activation may be driven by glucotoxicity and lipotoxicity in adipocytes, hepatocytes, skeletal muscle, and the stromal macrophages of these tissues [971,972,973]. In adipose tissue under meta-inflammatory conditions, ferroptotic death can occur in adipocytes, M2 macrophages, and Tregs [974]. Ferroptosis in pancreatic β-cells reduces insulin secretion, whereas ferroptosis in liver, adipose tissue, and muscle induces IR [975]. Deficiency of cystathionine-γ-lyase in skeletal muscle leads to ferroptosis and subsequently to IR, hyperglycemia, and obesity [976]. Conversely, activating ferroptotic signaling with a nonlethal dose of ferroptosis agonists markedly decreases lipid accumulation in adipocytes in high-fat–fed mice and protects them from obesity [977]. The role of another programmed necrosis—necroptosis—in murine MAFLD/NAFLD is ambiguous and controversial [978,979]. Inactivation of necroptotic signaling in an obesity model enhances IR and adipocyte apoptosis [980].

Collectively, tissue breakdown products (including DAMPs), macrophage-derived stress mediators, gut-derived endotoxemia, and ongoing metabolic injury can facilitate a shift from predominantly local to more systemic meta-inflammatory involvement, recruiting additional organs and vascular compartments. In this progression, cardiovascular and endothelial responses become decisive determinants of downstream morbidity.

#### 6.2.3. Endotheliosis

Endotheliosis is a key manifestation of systemic meta-inflammation associated with atherosclerosis and other cardiovascular pathologies. As defined above, “endotheliosis” denotes a low-grade endothelial stress phenotype typical of systemic meta-inflammation; here we detail its principal molecular and functional manifestations and their links to IR.

The development of endothelial dysfunction within systemic meta-inflammation is stage-dependent: it is typically more pronounced in established T2DM and less evident in prediabetes [981]. Pathological endothelial activation is multifactorial and, in addition to glucotoxic and lipotoxic injury, can reflect the combined influence of DAMPs and PAMPs (metabolic endotoxemia), pro-inflammatory cytokines and histohormones, ROS and other mediators generated during intravascular leukocyte activation and thrombophilia, stress-associated miRNAs, modified atherogenic lipoproteins, and, in the context of frequent cardiopulmonary comorbidity, hypoxia-driven cellular stress [226,827,828,829,830,831,832,833,834,835,836,837,838,839,840,841,842,843,844,845,846,847,848,849,850,851,852,853,854,855,856,857,858,859,860,861,862,863,864,865,866,867,868,869,870,871,872,873,874,875,876,877,878,879,880,881,882,883,884,885,886,887,888,889,890,891,892,893,894,895,896,897,898,899,900,901,902,903,904,905,906,907,908,909,910,911,912,913,914,915,916,917,918,919,920,921,922,923,924,925,926,927,928,929,930,931,932,933,934,935,936,937,938,939,940,941,942,943,944,945,946,947,948,949,950,951,952,953,954,955,956,957,958,959,960,961,962,963,964,965,966,967,968,969,970,971,972,973,974,975,976,977,978,979,980,981,982,983]. Evidence consistent with intravascular phagocyte activation in meta-inflammation and T2DM includes leukocyte adhesion to the endothelium and formation of neutrophil extracellular traps (NETs), which can promote thrombophilia and directly injure endothelial cells [984,985,986,987,988].

Under hyperglycemia, endothelial cells can experience excessive glucose influx; glucose is partly diverted into the polyol pathway and reduced to sorbitol, which contributes to osmotic imbalance and facilitates aberrant intracellular glycation reactions [989].

The principal manifestations of endotheliosis include degradation of the endothelial glycocalyx (thereby facilitating endothelial permeability to atherogenic lipoproteins), reduced NO bioavailability, stress-induced remodeling of receptor and secretory phenotypes, release of stress-associated miRNAs and procoagulant factors, and activation-dependent interactions with intravascular leukocytes and with the complement, hemostasis, and kallikrein–kinin systems. Glycocalyx disruption in MetS and T2DM contributes not only to atherosclerosis, diabetic nephropathy, and other diabetic angiopathies, but also to increased blood–brain barrier permeability, particularly under additional inflammatory stressors (including viral infection contexts) [990,991,992,993,994,995,996,997].

Reduced NO bioavailability reflects both impaired production and NO inactivation under heightened oxidative stress [998,999,1000]. A principal mechanism for diminished NO production in MetS/T2DM is inhibition of the PI3K/Akt/eNOS axis by pro-inflammatory stress signaling (including JNK, NF-κB, PKC isoforms, and ROS) [1001,1002,1003,1004,1005,1006]. Together with renin–angiotensin–aldosterone activation, endothelin-1 (ET-1) upregulation, and smooth-muscle hypertrophy in resistance vessels, these changes contribute to the hypertensive phenotype in MetS and T2DM [1001,1002,1003,1004,1005,1006]. In this framework, endothelial insulin signaling becomes selectively imbalanced: the PI3K/Akt “metabolic/vasodilatory” arm is suppressed, whereas SHC/MAPK signaling—including SHC/MAPK-driven ET-1 induction—is relatively preserved or enhanced [1007]. The SHC/ERK1/2 route can further antagonize the IRS-1/PI3K/Akt/eNOS pathway, reinforcing endothelial dysfunction [1008].

Several pro-inflammatory transcription factors, including NF-κB, AP-1, and HIF-1, participate in ET-1 induction [1009,1010]. ET-1 then signals through ET-A receptors to activate MAPK-p38/NF-κB pathways, thereby exerting cytokine-like pro-inflammatory effects that can amplify endothelial activation [1010,1011].

In endothelial cells, compensatory hyperinsulinemia can preferentially engage SHC/MAPK signaling, promoting expression of inflammatory mediators and adhesion molecules (ICAM-1, VCAM-1, E-selectin) while further suppressing PI3K/Akt activity [1012]. The resulting imbalance contributes to a phenotype characterized by reduced NO signaling and increased ET-1 production [1013,1014]. Consistent with this pathogenic axis, elevated plasma ET-1 in non-diabetic individuals has been reported as an independent predictor of incident prediabetes and T2DM [1015]. ET-1 receptors are expressed across insulin-sensitive tissues (adipose tissue, liver, skeletal muscle), supporting the plausibility that ET-1 signaling contributes not only to vascular dysfunction but also to IR-relevant metabolic dysregulation at the tissue level [1016].

#### 6.2.4. Thrombophilia

Within the systemic process ensemble of meta-inflammation in T2DM, thrombophilia represents a consistent and clinically relevant component. It manifests as hyperfibrinogenemia (reflecting hepatic acute-phase activation), thrombinemia, intravascular platelet activation and aggregation, intravascular “paracoagulation,” and impaired fibrinolysis [910,1017,1018,1019]. Collectively, these shifts increase the risk of arterial thrombosis, myocardial infarction, and stroke. Importantly, a prothrombotic tendency may be detectable already at early IR stages, including prediabetes, which supports risk-stratified consideration of antiplatelet or anticoagulant strategies in patients with T2DM who have established cardiovascular disease or a high thrombotic-risk profile [1020,1021]. Beyond glucose control, several glucose-lowering agents have been reported to exert direct anticoagulant or antithrombotic effects in T2DM, further underscoring the mechanistic coupling between metabolic and hemostatic dysregulation [1020]. From a practical standpoint, shortened prothrombin time, shortened activated partial thromboplastin time, and elevated fibrinogen may serve as accessible hemostatic markers of a prothrombotic phenotype in diabetes, particularly in patients at increased risk for thrombotic complications [1022].

#### 6.2.5. Chronic Microcirculatory Disorders in T2DM

The microcirculation comprises vessels <150 μm in diameter (arterioles, capillaries, venules) and is essential for tissue perfusion, gas exchange, and clearance of metabolic by-products. Chronic systemic microcirculatory disorders (MCDs) encompass heterogeneous conditions that compromise small-vessel patency and function, thereby promoting tissue injury and multi-organ dysfunction [1023]. In T2DM, chronic MCDs develop against a background of systemic remodeling of the microvascular bed, including altered capillary-network density in organs that are particularly vulnerable in diabetes [1024]. Microvascular complications—retinopathy, nephropathy, cardiomyopathy, and neuropathy—are prototypical outcomes of small-vessel injury and can progress to organ dysfunction; glucotoxic pathways (including polyol flux in endothelial cells) and oxidative stress are among the major mechanistic contributors [1025,1026]. Importantly, T2DM-associated MCDs may also affect “nontraditional” target tissues (e.g., brain, lungs, bone, skin, arterial wall, and musculoskeletal system), supporting the view that microvascular injury contributes to a broad, systemic phenotype rather than a confined set of classical complications [1027].

Clinically significant systemic MCDs typically accompany severe T2DM complications and are closely aligned with meta-inflammatory mechanisms. A key unresolved issue, however, is which inflammatory “intensity states” are most relevant, given that a substantial subset of patients may exceed low-grade systemic inflammation and exhibit a chronic hyperinflammatory phenotype (19% in our cohort) [928]. Two conditions in which chronic hyperinflammation is present in most patients are end-stage renal disease (ESRD) and systemic lupus erythematosus (SLE) [926]. In both settings, systemic endotheliosis and clinically significant MCDs are consistently reported, indicating that sustained hyperinflammation can converge on microvascular pathology across distinct etiologies [880,1028,1029,1030,1031,1032,1033,1034,1035,1036,1037].

Notably, the association between IR and non-diabetic chronic kidney disease is detectable before ESRD, and IR in these patients remains an independent predictor of cardiovascular disease [1038,1039,1040]. In ESRD, IR frequently develops in the context of energy-store depletion (including sarcopenia), undernutrition, and malabsorption. In such cases, IR may be compensated by hyperinsulinemia rather than overt hyperglycemia, yet it remains linked to systemic dysfunction, hypercytokinemia, and increased circulating levels of CRP, FFAs, leptin, resistin, and adiponectin [1040,1041,1042,1043,1044,1045]. The relationship between SLE and IR is likewise well supported. While type B insulin-resistance syndrome mediated by anti–insulin-receptor antibodies can occur [1046], in most patients IR in SLE is not primarily autoimmune in mechanism; instead, it behaves as an independent risk factor for cardiovascular and other internal-organ disease, shaped by classical IR drivers (including glucocorticoid exposure) and by cumulative tissue injury and inflammatory burden [1047,1048,1049,1050,1051,1052,1053,1054,1055]. Together, these observations support a central role for local and systemic meta-inflammation—interacting with lipotoxicity and glucotoxicity—in IR pathogenesis, while also indicating that other forms of chronic systemic inflammation can contribute to moderate IR and may accelerate IR progression in established T2DM.

### 6.3. The Role of Scavenger Receptors in the Pathogenesis of IR and the Development of Meta-Inflammation

Scavenger receptors (SRs) comprise >30 members that are classified on structural grounds into 11 classes (A–L). They are expressed predominantly by stromal macrophages, and their expression often increases with ligand burden, consistent with an inducible “clearance” program. SRs are genetically and architecturally heterogeneous, yet they share a functional logic: broad recognition and handling of altered-self and damage-associated ligands. Across SR classes, reported ligands include modified lipoproteins, glycated proteins, aggregated platelets, apoptotic/senescent/damaged cells, and other endogenous products that can be operationally viewed as metabolic and cellular “debris,” as well as limited microbial material. A central property of SR systems is their contribution to cellular and tissue stress regulation, in part through receptor cooperativity—including assembly with integrins, Toll-like receptors (TLRs), and other signaling modules. Importantly, SR engagement is not uniformly pro-inflammatory: depending on receptor identity, ligand context, and cellular state, SR signaling may support homeostatic resolution programs or, conversely, amplify meta-inflammatory circuits. Thus, SR-mediated pathways operate at the interface between physiology and pathology and constitute plausible mechanistic nodes in somatic diseases linked to meta-inflammation and insulin resistance, as summarized in Table 13 [679,1056,1057,1058,1059,1060].

Table 13 briefly summarizes data on the principal effects of selected SRs in the pathogenesis of meta-inflammation and IR. The pathogenetic role of SR-B2 (CD36) in atherosclerosis, meta-inflammation, and IR merits particular emphasis. CD36 is simultaneously a receptor for FFAs and is actively involved in the pathogenesis of endotheliosis and atherosclerosis, as well as in macrophage activation by endogenous factors, including as a co-receptor for TLR4, TLR6, and TLR2 [684,1114]. SR-J1 (RAGE)—the principal receptor for advanced glycation end products (AGEs)—also warrants special mention. In terms of pro-inflammatory activity, especially in diabetes, SR-J1 (RAGE) is functionally closest to classical PRRs such as TLRs [1100,1115,1116]. In addition, CD36 on vascular macrophages, endothelial cells, and platelets participates—together with SR-J1 (RAGE) and the endothelial SR-E1 (LOX-1)—in the development of systemic inflammation [679].

Overall, SRs can promote meta-inflammation by activating endothelial cells—particularly through SR-E1 (LOX-1)—and macrophages, including through recognition and uptake of excess atherogenic lipoproteins. At the same time, SRs can constrain inflammatory responses by regulating cellular-stress activity and by enabling efferocytosis, through the recognition by macrophages of phosphatidylserine and other apoptotic markers on the surface of dying cells.

### 6.4. Causes of IR Unrelated to Alimentary Obesity and Their Links to Inflammation

Beyond autoimmune type B insulin-resistance syndrome, rare non-obesity-related causes of IR and T2DM include several severe-IR syndromes with a genetic basis. These encompass: (i) InsRec defects underlying type A insulin resistance and the Donohue and Rabson–Mendenhall syndromes; (ii) disorders of downstream signaling such as PIK3R1-associated SHORT syndrome, as well as abnormalities in AKT2 or AS160/TBC1D4; and (iii) clinically defined entities in which the causal gene remains unidentified [1117,1118,1119,1120,1121]. In addition, congenital lipodystrophy syndromes—caused by generalized or partial absence of adipose tissue due to defects in leptin biology and other adipose-development proteins—are rare multisystem disorders characterized by insulin-resistant diabetes, severe hypertriglyceridemia, and ectopic lipid deposition (notably in the liver), with organ-specific complications [1117,1119,1122].

Direct evidence specifically dissecting inflammation–IR coupling in hereditary severe-IR syndromes remains limited. Nevertheless, autoimmune systemic diseases provide a clearer precedent. In SLE (discussed above), IR is well documented in parallel with chronic inflammatory activation. Rheumatoid arthritis offers another illustrative example: IR severity—beyond the effects of prednisone and coexisting obesity—tracks with systemic inflammatory burden and associates with increased cardiovascular mortality risk [1123,1124,1125,1126,1127,1128,1129].

In recent years, chronic stress and depression have gained recognition as independent risk factors for reduced insulin sensitivity and incident diabetes. Chronic psychoemotional stress, sustained hypothalamic–pituitary–adrenal activation, and—under some conditions—distress-related dysregulation promote latent IR and correlate with cardiovascular complications [838,1130,1131,1132,1133]. Major depressive disorder, a frequent sequela of chronic stress, is likewise linked to CNS–gut axis dysfunction and inflammatory activation [107,108,109]. A substantial literature supports bidirectional associations between depression and IR/metabolic syndrome, as well as between depression and systemic low-grade inflammation, collectively implying convergent neuroendocrine and immunometabolic mechanisms [184,1134,1135,1136,1137,1138,1139,1140,1141,1142,1143,1144,1145,1146,1147,1148,1149].

Many viral infections can induce transient adaptive IR and, in some settings, more prolonged impairments in insulin sensitivity [1150,1151,1152]. Several infections are associated with both latent IR and low-grade inflammation [1153]. Low-grade systemic inflammation is increasingly discussed as a component of Long COVID [829,1154,1155,1156,1157], plausibly interacting with viral persistence or reactivation—particularly of herpesviruses [1155,1156,1157,1158,1159]. Reactivation of HHV-4/5/6 has been linked to systemic inflammatory phenotypes, and in specific contexts, herpesviruses have also been implicated in severe systemic pathology [829,1158]. Prospective and retrospective studies indicate higher rates of IR and newly diagnosed T2DM in Long COVID compared with uncomplicated post-COVID recovery; Long COVID may also aggravate pre-existing metabolic abnormalities, with post-infection increases in insulin levels and HOMA-IR relative to baseline [1160,1161,1162,1163,1164,1165,1166,1167,1168,1169,1170,1171,1172,1173]. Associations between herpesvirus serostatus (e.g., HSV-2 and CMV) and latent IR have been reported, but remain limited and require independent verification [834,1174,1175,1176,1177]. More consistent evidence exists for HIV: HIV is associated with systemic low-grade inflammation [1178,1179,1180] and with latent IR, increased metabolic-syndrome risk, and adverse T2DM trajectories attributable to both viral effects and antiretroviral therapy [1181,1182,1183,1184,1185,1186,1187,1188,1189,1190]. Viral hepatitis pathogens similarly promote hepatic IR and can aggravate T2DM [1191,1192,1193,1194,1195,1196,1197,1198,1199,1200].

Among chronic bacterial infections, tuberculosis merits attention because obesity is not typical, yet active disease exhibits endotheliosis and systemic inflammation [1201,1202,1203,1204] and is associated with latent IR; when T2DM coexists, tuberculosis often has more severe clinical manifestations [1205,1206,1207]. Chronic *Helicobacter pylori* infection has been proposed as a contributor to latent IR and unfavorable T2DM dynamics [1208]. *Porphyromonas gingivalis*, central to periodontitis, has been linked to low-grade inflammation and cardiovascular pathology and may likewise worsen IR through persistent inflammatory signaling [1209,1210,1211].

Finally, critical illness (severe trauma, sepsis) is characterized by hypercatabolism and acute systemic hyperinflammation, with marked hyperglycemia and situational IR [1212,1213,1214,1215,1216,1217,1218,1219,1220]. However, whether aggressive insulin-mediated glycemic control improves outcomes remains debated; some studies report no mortality benefit and potential harm, underscoring that the pathogenetic role of stress hyperglycemia/IR in critical states is context-dependent.

In summary, while local and systemic meta-inflammatory mechanisms remain central drivers of IR, other chronic inflammatory states—including infectious and stress-related phenotypes—can measurably impair insulin sensitivity across tissues and may act as additional risk modifiers for incident and progressive T2DM.

### 6.5. Genetics of Predisposition to IR and T2DM and Its Links to Inflammation

Recent genome-wide association studies (GWAS) have markedly expanded the catalogue of loci associated with insulin resistance (IR) and type 2 diabetes mellitus (T2DM), now numbering >500 [1221,1222,1223,1224]. Most signals map to genes involved in pancreatic β-cell development and function, insulin signaling, and glucose–lipid homeostasis, supporting the view that T2DM reflects not only peripheral IR but also intrinsic liabilities in insulin production and secretion. Among individual loci, *TCF7L2* shows one of the strongest and most reproducible effects on T2DM susceptibility. *TCF7L2* encodes a Wnt-pathway transcriptional regulator implicated in β-cell development and function; common variants such as rs12255372 and rs7903146 are robustly associated with increased T2DM risk [1225].

Family history of T2DM has also been linked to increased vulnerability to oxidative stress and inflammatory phenotypes [1226]. In parallel, many polymorphisms associated with IR/T2DM intersect pathways that regulate macrophage polarization and stress–inflammation signaling, including TLR- and cytokine-dependent networks converging on MAPK–JNK, NF-κB, and NFE2L2/Nrf2 and related cellular-stress regulators [1227]. Population-level variability is substantial for SNPs in cytokine gene clusters relevant to T2DM pathogenesis (IL-1α, IL-1β, IL-1Ra, IL-6, IL-10, IL-18, TNF-α) [1228]. At the same time, this overlap should not be overinterpreted as implying a “type 1 diabetes–like” autoimmune genetic architecture for T2DM. Notably, Rafiq et al. reported no evidence that 46 variants known to influence autoimmune or inflammatory disease risk—covering both variants that modulate circulating inflammatory proteins (e.g., IL-18, IL1RN, IL6R, MIF, CRP) and variants in autoimmune-predisposition loci, including class II HLA—alter T2DM risk [1229]. This contrast highlights a key etiological distinction: T2DM genetics preferentially emphasizes metabolic and β-cell pathways, whereas type 1 diabetes is dominated by immune-autoimmune determinants.

Polygenic risk for obesity and T2DM can be amplified or attenuated by environmental exposures through epigenetic programming, including DNA methylation/acetylation and histone modifications that regulate gene expression [1230,1231]. Multiple studies link diet, physical activity, and aging to T2DM trajectories through epigenetic modulation of β-cell insulin production and metabolic pathways in liver, skeletal muscle, and adipose tissue [1232,1233,1234]. In T2DM, altered DNA methylation has been reported in genes central to insulin action (e.g., PPARG, KCNQ1, TCF7L2, IRS1) across insulin-responsive tissues, as well as in regulatory axes with inflammatory and vascular relevance (e.g., TGF-β, NF-κB, AngII, ET-1) [1234]. Obesity and T2DM are also associated with epigenetic remodeling of stromal macrophages, affecting polarization states, proliferation, and cytokine output [1235]. In endothelial cells, promoter hypomethylation and increased histone acetylation at NF-κB-related loci—together with enhanced H3K9/K14 acetylation—have been linked to increased expression of NF-κB targets (e.g., IL-8), HMOX1, and other inflammatory markers [1236]. Hyperglycemia-associated chromatin changes have also been connected to increased MMP-9 expression, plausibly predisposing to endothelial injury [1237]. Several microRNAs (e.g., miR-140-5p, miR-221-3p, miR-200b, miR-130b-3p) have been implicated in endothelial dysfunction by targeting networks controlling apoptosis, inflammation, hyperpermeability, senescence, and pathological angiogenesis [1238]; additionally, higher circulating miR-126 has been reported to correlate with endothelial dysfunction in peripheral arterial disease in T2DM [1239].

Taken together, genetic and epigenetic evidence support two complementary conclusions. First, metabolic dysregulation in insulin-responsive tissues and β-cell liability are central to the genetic architecture of T2DM. Second, inflammatory and cellular-stress pathways recurrently intersect these metabolic programs, providing mechanistic plausibility for the tight coupling between IR/T2DM, meta-inflammation, and vascular dysfunction—while remaining distinct from the autoimmune architecture that defines type 1 diabetes.

## 7. Conclusions

Based on the available evidence, the primary drivers of IR are metabolic disturbances in insulin-responsive tissues (liver, skeletal muscle, and adipose tissue) and impaired insulin production by β-cells at the stage of T2DM. These changes typically arise against a background of morbid obesity and tissue aging. The principal metabolic consequences of IR include reduced expression of the glucose transporter GLUT4 in adipocytes and skeletal muscle and an inappropriate increase in hepatic glucose output, primarily via gluconeogenesis. Under physiological conditions, InsRec metabolic function is regulated by negative feedback through tyrosine phosphatases that counteract receptor autophosphorylation and downstream signaling, as well as by serine and serine/threonine kinases that attenuate InsRec/IRS signaling. During IR, these regulatory brakes become chronically overengaged and are reinforced by stress-kinase inputs, producing the characteristic metabolic phenotype described above.

These changes are accompanied by elevated circulating glucose, FFAs, and atherogenic lipoproteins, together with an altered balance of adipokines, myokines, hepatokines, and regulatory miRNAs. Dysregulated metabolism and the injurious effects of glucotoxicity and lipotoxicity then progressively involve the CNS–gut axis, with shifts in the microbiome and intestinal barrier dysfunction, leading to meta-inflammation within insulin-responsive tissues and, subsequently, at the systemic level. In contrast to protective variants of canonical inflammation, this form fails to localize and eliminate injurious stimuli. Instead, despite partial compensatory mechanisms, meta-inflammation sustains a self-reinforcing loop between stress signaling and insulin-pathway inhibition, promotes IR and endothelial dysfunction (endotheliosis), and—under hyperinflammatory conditions—may progress toward overt endotheliitis, thereby contributing to cardiovascular disease and other T2DM complications.

Inflammatory mechanisms that shape unfavorable IR dynamics are complex and internally contradictory. Moreover, pro-inflammatory cellular-stress pathways can be homeostatic when activated transiently or moderately and when they remain below the threshold for overt inflammation. This complexity helps explain why targeted anti-inflammatory strategies have shown uneven translational success in T2DM and why off-target or systems-level adverse effects can emerge when single nodes are suppressed in isolation.

Nevertheless, preventive and therapeutic benefits at early stages of IR are achievable through lifestyle interventions, including rational dietary patterns and physical activity; mitigation of chronic psycho-emotional stress; timely control of chronic inflammatory diseases of diverse etiologies; normalization of gastrointestinal function; and the use of low-intensity, multimodal anti-inflammatory approaches with favorable safety profiles (including selected phytotherapeutic and traditional medicine strategies) as adjuncts rather than substitutes for evidence-based metabolic care.

## Figures and Tables

**Table 1:** PKC families and representatives, activation, and roles in inhibiting InsRec function [259,260,261,262,263,264,265,266,267].

PKC Family Groups and Representatives	PKC Activation	The Role of PKC in Inhibiting InsRec Function
**Conventional PKC**: cPKCα, cPKCβ1, cPKCβ2, cPKCγ	Activated by Ca^2+^, DAG, phospholipids	Likely lower inhibitory impact than other PKCs
**Novel PKC**: nPKCδ, nPKCε, nPKCη, nPKCθ	Activated by DAG	High inhibitory impact (especially PKCε)
**Atypical PKC**: aPKCι, aPKCζ	Activated by IRS/PI3K class I, IP3, ceramide	High inhibitory impact

Note. PKC, protein kinase C; DAG, diacylglycerol; IRS, insulin receptor substrate; PI3K1, class I PI3-kinases; IP3, inositol trisphosphate.

**Table 2:** Effects of Canonical Cellular-Stress Modules in Major Insulin-Sensitive Tissues on the Development of Insulin Resistance.

Canonical Cellular-Stress Phenomena (CS)	General Characteristics	Links with Insulin Resistance	Evidence Base (Dominant; Illustrative Refs)
**Oxidative stress (OS)** [282,283,284,285,286,287,288,289,290]	Redox imbalance with excess ROS; main sources: mitochondria and ER/microsomal oxidation; redox signaling intersects other CS modules; physiological ROS/NO supports normal InsRec signaling.	In metabolic overload/T2DM, excess ROS is linked to AGE formation and PKC activation and is associated with impaired InsRec signaling; OS suppresses GLUT4 transcription (adipocytes) and impairs GLUT4 translocation (muscle), while antioxidants may partially restore trafficking in experimental settings; OS impairs β-cell insulin-gene regulation (e.g., *Pdx-1, MafA*) and promotes β-cell loss; conversely, hyperglycemia/FFA/oxLDL and inflammatory lipokines amplify OS; reduced eNOS activity/NO bioavailability is linked to endothelial dysfunction and is associated with hepatic and muscle IR.	**M/A**/H (e.g., M/A: [282,284,285,289]; H: [283,286,287,288,290])
**Mitochondrial stress (MS)** [33,287,291,292,293,294,295,296,297,298,299]	Disturbed mitochondrial dynamics with ROS overproduction; UPRmt activation and altered mito-nuclear signaling; mitophagy supports clearance/recovery; incomplete resolution may progress to ATP deficit, cell death and inflammatory amplification; sirtuin programs support respiration/antioxidant defense.	MS accompanies IR across hepatocytes, adipocytes, myocytes, endothelium and β-cells in metabolic syndrome/T2DM, commonly driven by lipotoxic factors (LCFAs and derivatives); consequences include ROS amplification, maladaptive UPRmt/mitophagy and inflammatory signaling that can impair insulin responsiveness and GLUT4 trafficking; interventions improving mitochondrial function or sirtuin-associated programs have been linked to improved insulin sensitivity, but effects remain tissue- and stage-dependent.	**M/A**/H (e.g., M/A: [291,292,293,294,295,298,299]; H: [287,296,297])
**Endoplasmic-reticulum stress (ER stress)** [33,300,301,302,303,304,305,306,307]	UPR_ER_ triggered by misfolded protein accumulation; restores ER homeostasis via translation attenuation, chaperones and ERAD; intersects with apoptosis, autophagy and MS; links to inflammatory signaling (NF-κB, NLRP3).	Obesity is associated with chronic ER stress in liver/adipose tissue; maladaptive UPRER can mechanistically link metabolic overload to impaired insulin signaling; reported links include PKCε-mediated InsRec inhibition, JNK-dependent inhibitory phosphorylation of IRS1, NF-κB activation and Ca^2+^-dependent stress cascades; ER stress can skew macrophage polarization toward M1, sustaining meta-inflammation; adaptive UPRER may be comparatively protective, and ER stress–associated autophagy can modulate IR outcomes in a context-dependent manner.	**M/A**/H (e.g., M/A: [300,301,302,303,305,307]; H: [304,306])
**Heat shock proteins (HSPs)** [33,288,308,309,310,311,312,313,314,315,316,317,318,319,320]	The HSP response is an evolutionarily conserved molecular reaction to disturbances of protein homeostasis (proteostasis). Major HSP chaperone functions are: (1) folding/packaging of nascent proteins and broader chaperone roles across tissues; (2) regulation of stress signaling pathways (pro- and anti-apoptotic); (3) participation in UPR_ER_ and UPR_mt_; (4) trafficking of steroid-hormone receptors (HSP90 family) and immune interactions; (5) activation/inhibition of autophagy factors; roles in cell cycle, differentiation, secretion, endocytosis, inflammation, and survival. HSP induction is governed by HSF family transcription factors and by stress kinases, including MAPK (JNK, p38, ERK) and CaMKII.	In T2DM, HSP levels are tissue-specific – elevated in some, reduced in others. Circulating extracellular HSP70 is markedly increased, whereas intracellular HSP70 may be insufficient to exert protective anti-inflammatory effects. Intracellular HSP72 and HSP73 are reduced in insulin-sensitive tissues (skeletal muscle, heart, liver) in T2DM. Overall, intracellular HSP upregulation is often inadequate to stabilize or reverse CS associated with IR, pro-inflammatory cytokine production, mitochondrial dysfunction, and ER stress. A vicious circle ensues: metabolic distress and maladaptive meta-inflammation drive IR, which impairs HSP function and further fuels inflammation. Elevated extracellular HSP70 in blood may serve as a potential biomarker of T2DM.	**M/A**/H (e.g., M/A: [308,315,317]; H: [309,310,311,312,313,314,316,318,319,320])
**Autophagy** [33,321,322,323,324,325,326,327,328,329,330]	Conserved lysosomal recycling pathway removing damaged proteins/aggregates, inflammasome components and dysfunctional organelles (incl. mitophagy); typically compensatory/homeostatic; regulated by PI3K/Akt/mTORC1 and AMPK.	Because insulin signaling suppresses autophagy via Akt/mTORC1, progressive IR and pro-inflammatory stress can relieve this restraint and increase autophagy as a compensatory response; in obesity, autophagy rises in adipocytes (notably visceral depots) alongside ER/MS/OS and may support survival under lipotoxicity/hypoxia and stabilize adipogenic programs (e.g., PPARγ); many experimental studies report that autophagy impairment worsens insulin signaling, but negative findings exist in selected settings (e.g., skeletal muscle lipid-induced IR), indicating context-dependent effects.	**M/A**; Context-dependent (incl. negative findings) (e.g., M/A: [321,322,323,324,325,326,327,328,329,330])
**Inflammasomes** [33,331,332,333,334,335,336,337,338,339,340,341,342]	Cytosolic NLR/ALR sensor complexes activating caspase-1, processing IL-1β/IL-18 and driving pyroptosis; assembly requires priming (NF-κB) and is facilitated by OS, ionic fluxes and metabolic danger signals (notably NLRP3).	NLRP3 activation is frequently linked to IR via IL-1β–driven inflammatory amplification and mechanisms impairing hepatic insulin action; the NF-κB/NLRP3/caspase-1 axis has been implicated in adipose and hepatic IR and in IR-linked phenotypes (e.g., MASLD/NAFLD, sarcopenia); however, downstream cytokine balance is not uniformly deleterious (e.g., IL-18 may limit lipid accumulation in some contexts), suggesting that magnitude and direction of effects can be context-dependent.	**M/A**/H; Context-dependent (e.g., M/A: [331,332,333,335,336,337,338,339,340,341]; H: [334,342])
**DNA-damage response (DDR)** [33,343,344,345,346,347,348,349]	DNA repair and checkpoint network sensing genomic lesions; interfaces with PI3K/AKT/mTORC, PI3K/AKT/p53 and Ras/MEK/ERK; links stress surveillance with cell-cycle control and survival.	Obesity and T2DM are associated with increased DNA damage, senescence and DDR markers in insulin-sensitive tissues; visceral adipose tissue shows activation of canonical DDR nodes (ATM/ATR, γ-H2AX, Chk1/Chk2, etc.); insufficient DDR capacity may accelerate tissue aging and metabolic syndrome, while in animal models genetic DDR restriction can reduce β-cell mass and insulin output; conversely, maladaptive DDR hyperactivation in certain tissues may amplify stress programs and worsen IR, indicating tissue- and stage-dependent effects.	**M/A**/H; Context-dependent (e.g., M/A: [343,345,347,348,349]; H: [344,346])
**MicroRNAs (miRNA)** [33,350,351,352,353,354,355,356,357,358,359,360,361,362,363,364,365,366,367,368,369,370,371,372,373]	Stress-responsive non-coding RNAs (18–25 nt) regulating gene expression; many circulate as extracellular vesicle-associated miRNAs mediating intercellular and inter-organ communication; effects are pleiotropic and network-based.	Multiple miRNAs are linked to IR as mechanistic modulators and as circulating biomarkers; examples include miR-34a (adipocyte insulin signaling; MASLD/NAFLD association), circulating miR-144/miR-29a/miR-142 as predictors of IR/T2DM risk, and β-cell miRNAs affecting insulin secretion/proliferation (e.g., miR-375/miR-155); exosomal miRNAs mediate adipose–muscle and macrophage–adipocyte crosstalk (e.g., miR-27a; macrophage-derived miR-210-3p affecting GLUT4 and insulin sensitivity); human biomarker evidence is predominantly associative, while mechanistic causality is supported mainly by experimental models; overall effects are highly context-dependent.	**H**/M/A; Context-dependent (e.g., M/A: [351,354,361,362,363,364,366,367,368]; H: [350,352,353,355,356,357,358,359,360,365,369,370,371,372,373])

Note. (1) AGE, Advanced Glycation End Product; ALR, Absent in Melanoma 2 (AIM2)-Like Receptor; ATM, Ataxia-Telangiectasia Mutated Kinase; ATP, Adenosine-5′-Triphosphate; ATR, ATM and Rad3-Related Kinase; CS, Cell Stress; DDR, DNA Damage Response; ER, Endoplasmic Reticulum; ERAD, Endoplasmic Reticulum-Associated Degradation; FFAs, Free Fatty Acids; FR, Free Radical; GLUT4, Glucose Transporter Type 4; HSP, Heat-Shock Protein; IL, Interleukin; InsRec, Insulin Receptor; IR, Insulin Resistance; JNK, c-Jun Metabolically Associated Steatotic Liver Disease; miRNA, MicroRNA; mTOR, Mammalian Target of Rapamycin; NAFLD, Non-Alcoholic Fatty Liver Disease; NF-κB, Nuclear Factor kappaB; NLR, Nucleotide-Binding Domain and Leucine-Rich Repeat Receptor; OS, Oxidative Stress; MS, mitochondrial stress; oxLDL, Oxidized Low-Density Lipoprotein; PI3K, Phosphoinositide 3-Kinases; PKC, Protein Kinase C; PPAR, Peroxisome Proliferator-Activated Receptor; PRR, Pattern Recognition Receptor; ROS, Reactive Oxygen Species; T2DM, Type 2 Diabetes Mellitus; TAG, Triacylglyceride; UPR, Unfolded Protein Response. (2) Evidence-based codes denote predominant evidence supporting each link: M (mechanistic/in vitro), A (animal in vivo), H (human observational/interventional). “Context-dependent” indicates heterogeneity across tissues, disease stages, or models; table statements summarize heterogeneous evidence and do not imply uniform causality across endpoints.

**Table 3:** Effects of Cellular-Stress Transcription Factors (TFs) on the Development of Insulin Resistance and T2DM.

Factor	General Characteristics	Links with Insulin Resistance (Context-Dependent Where Applicable)
**NF-κB** [374,375,376,377,378,379,380,381,382,383,384]	Moderate activation can occur downstream of insulin/RTKs (e.g., Ras/ERK; PKC/ERK routes); high activity marks intense pro-inflammatory CS with OS, M1 polarization, inflammasome priming, senescence-like secretory programs and permissive survival/regulated necrosis phenotypes (pyroptosis/necroptosis).	In insulin-sensitive tissues under meta-inflammation, NF-κB inhibition commonly improves IR in experimental and clinical-oriented literature; induced by LCFAs via MS-related signaling and by AGEs via RAGE; IL-1β and TNF-α worsen IR via JNK/NF-κB and TNF/TRAF2/IKBKB/NF-κB cascades; NF-κB amplifies IL-1β/TNF-α signaling, receptor expression, MAPK tone and oxidative burden, reinforcing IR-related inflammatory loops.
**p53** [349,385,386,387,388,389,390,391,392]	Central DDR node integrating survival/repair vs. apoptosis under irreversible damage; modulated by insulin/RTKs and GPCRs; can be protective or maladaptive in chronic meta-inflammation.	In T2DM models, p53 tends to increase across insulin-sensitive tissues, but effects on insulin signaling are divergent; p53/TRIB3 can inhibit insulin signaling yet restrain NF-κB/MAPK/ATF4-driven inflammatory tone; p53 contributes to adipose macrophage accumulation partly via p53-driven adipocyte apoptosis; p53 polymorphisms associate with either higher or lower T2DM/IR predisposition (bidirectional genetic associations).
**AP-1** [393,394,395,396,397,398,399,400,401]	Jun/Fos heterodimeric TF (can interact with ATF proteins); governs proliferation/differentiation in physiology; rises sharply during inflammation and executes multiple inflammatory programs.	Supports IRS-1 trafficking and insulin/IGF-1 signaling architecture; TLR4/AP-1 and TNF-α/JNK/AP-1 drive hepatocyte meta-inflammation, hepatic steatosis, IR worsening and M1 skewing; in adipocytes, TNF-α/MAPK/AP-1 promotes MCP-1/CCL2 (monocyte recruitment); PKCθ/AP-1 axis contributes to skeletal muscle physiology and is implicated in muscle IR; inflammatory cytokines (TNF-α, IL-6, IL-1β) and JNK/NF-κB signaling amplify AP-1, reinforcing adipocyte IR.
**HIF-1α** [402,403,404,405,406,407,408,409,410,411,412,413,414]	Activity determined by α-subunit stability; hypoxia is primary inducer, but non-hypoxic stress pathways also stabilize HIF-1α; insulin can stabilize HIF-1α via PI3K/AKT even in normoxia; HIF-1 regulates angiogenesis (adaptive or pathological) and upregulates GLUT1/GLUT3 independent of insulin.	Hypoxia/HIF-1α commonly associates with worsened IR in liver, muscle and adipose tissue; correlates with hyperglycemia, OS and meta-inflammation; obesity/HFD can create relative adipocyte hypoxia via impaired respiration → HIF-1α induction and adipose inflammation; hypoxia-related miRNAs (e.g., miR-128) may negatively regulate InsRec in visceral adipocytes; effects are context-dependent: HIF-1α-targeted strategies show limited proven clinical benefit; HIF-1α deletion can worsen complications/β-cell function, whereas hyperactivity can also disrupt β-cell function; IR may impair HIF-1α signaling and hypoxia adaptation.
**HSF-1** [33,415,416,417,418,419,420,421]	Master regulator of HSP induction (UPRmt/UPRER); activated via MAPKs (JNK/p38/ERK), CaMKII and proteotoxic cues; HSP90/HSP70/HSP40 provide negative feedback; supports proteostasis, longevity and stress resilience; insulin limits HSF-1 via PI3K/AKT negative feedback; constitutive β-cell HSF-1 can increase glucose-stimulated insulin secretion.	Exercise increases skeletal-muscle HSF-1; muscle HSF-1 overexpression shifts fibers toward oxidative metabolism, enhances FA oxidation and improves insulin sensitivity; HSF-1 dysfunction contributes to hepatic steatosis, morbid obesity and systemic IR; reduced insulin signaling (AMPK; PI3K/AKT changes) can activate HSF-1 but feedback is disrupted in T2DM, promoting dysfunction; HSF-1 protects β-cells under glucolipotoxicity and may preserve β-cell mass; polymorphisms associate with T2DM risk (supporting proteostasis/UPR involvement).
**NRF2** [33,422,423,424,425,426]	Redox-responsive TF inducing antioxidant programs via Keap1/NRF2; counter-regulates inflammatory severity partly by limiting NF-κB; regulated by complex transcriptional/post-translational network including NF-κB and AP-1 (both stimulatory and inhibitory cross-talk).	Effects on obesity/IR are reported as mixed across models; nevertheless, NRF2 is implicated in morbid obesity and IR and remains a therapeutic target class; activators are of interest though not established for T2DM clinical use; experimental examples support benefit via rebalancing NF-κB/MAPK tone and restoring antioxidant capacity (model-dependent).
**ATF4** [427,428,429,430,431,432,433,434,435,436,437,438]	Core UPRER TF activated by ER stress, amino-acid limitation, proteotoxic injury, hypoxia and OS; promotes survival under stress; links ER stress to autophagy; regulates amino-acid metabolism/transport, antioxidant defense, protein/lipid homeostasis; can also promote apoptosis/ferroptosis/senescence depending on intensity and context.	ER stress with hypothalamic ATF4 can induce hepatic IR via autonomic mechanisms in mice; ATF4 is required for normal β-cell insulin production and glucose-stimulated secretion; hepatocyte ATF4 can activate NRF2 and FGF21; in skeletal muscle, ATF4 signaling interacts with AKT/mTOR and can inhibit PGC-1α, with potential downstream effects on inflammation and insulin sensitivity; pharmacologic ATF4 inhibition may support β-cell proliferation/insulin output in some settings; resistance training reduces ATF4-activated and senescence-associated transcripts in aged muscle; overall, ATF4 effects in T2DM/meta-inflammation are context-dependent and sometimes contradictory.
**STAT (JAK/STAT pathway)** [108,439,440,441,442,443,444,445]	Central cytokine/hormone signaling axis (JAK1–3/STAT1–6) for multiple interleukins, interferons and metabolic hormones (e.g., GH, leptin); governs differentiation and stable function across cell types; activity rises during inflammation; tissue-specific genetic studies implicate JAK/STAT in glucose tolerance, insulin sensitivity, energy expenditure and obesity.	Restricting JAK/STAT together with NF-κB can mitigate IR and hyperglycemia in T2DM models; limiting STAT1/STAT3 signaling in metabolic organs is often reported as beneficial in experimental IR contexts; exercise-associated improvements in insulin sensitivity can coincide with reduced JAK/STAT activation and shifts toward anti-inflammatory macrophage profiles (mechanism varies by model); in obesity, chronic CNS leptin → STAT3 drives leptin resistance, while peripheral IL-6 → STAT3 can impair insulin action; STAT4 deficiency improves adipose inflammation and insulin signaling in mice; overall, JAK/STAT is a pivotal regulator of metabolism-related inflammation with context-dependent therapeutic tractability.

Note. AP-1, Activator Protein-1; ATF4, Activating Transcription Factor 4; CS, Cell Stress; DDR, DNA Damage Response; ER, Endoplasmic Reticulum; ERK, Extracellular Signal-Regulated Kinase; FGF, Fibroblast Growth Factor; G-CSF, granulocyte colony-stimulating factor; GM-CSF, granulocyte-macrophage colony-stimulating factor; GPCR, G-Protein-Coupled Receptor; HIF-1, Hypoxia-Inducible Factor1; HSF, Heat Shock Factor; HSP, Heat-Shock Protein; IFN, Interferon; IGF, Insulin-Like Growth Factor; IKBKB, Inhibitor of Nuclear Factor kappa B Kinase Subunit beta; IL, Interleukin; InsRec, Insulin Receptor; IR, Insulin Resistance; IRS1, Insulin Receptor Substrate-1; JAK, Janus Kinase; JNK, c-Jun N-terminal Kinase; LCFA, Long-Chain Fatty Acids; M1, Classically Activated Macrophages; MAPK, Mitogen-Activated Protein Kinase; MARK, Microtubule Affinity-Regulating Kinase; MCP-1, Monocyte Chemoattractant Protein-1; miRNA, MicroRNA; NF-κB, Nuclear Factor kappaB; Nrf2, Nuclear Factor Erythroid-2-Related Factor 2; PCG-1α, PPAR-γ coactivator-1α; PI3K, Phosphoinositide 3-Kinases; PKC, Protein Kinase C; RAGE, Receptor For Advanced Glycation End Products; RTK, Receptor Tyrosine Kinases; STAT, Signal Transduction and Activator of Transcription; T2DM, Type 2 Diabetes Mellitus; TF, Tissue Factor; TLR, Toll-Like-Receptor; TNF, Tumor Necrosis Factor; TRAF2, Tumor Necrosis Factor Receptor Associated Factor 2; UPR, Unfolded Protein Response.

**Table 4:** Effects of Anti-inflammatory Cytokines and Specialized Pro-resolving Lipid Mediators (SPMs) on Insulin Resistance.

Factors	Principal Functions	Links with Insulin Resistance
**Lipoxins (LXA4, LXB4)** [446,447,448,449,450,451,452]	Arachidonic-acid–derived SPMs; LXA4 is best characterized; main pro-resolving receptor FPR2/ALX (GPCR) expressed on immune cells and also endothelial/epithelial cells.	Low LXA4 combined with high visceral adiposity (WC/VFA) improves metabolic-syndrome prediction; higher LXA4 associates with lower incident T2DM risk; in vitro, LXA4 dampens adipose inflammation (↓IL-6, ↓TNF-α, ↑IL-10) with concordant increases in GLUT4 and IRS expression in adipocytes; clinical/experimental literature suggests potential benefits for diabetic CVD and diabetic kidney disease (associative + mechanistic support).
**Omega-3 FA–derived SPMs (resolvins, protectins, maresins)** [453,454,455,456,457,458,459,460,461,462,463]	Signal via GPCRs (FPR2/ALX, GPR18, BLT1, GPR32, GPR37, chemerin1) and through PPARγ; derived from omega-3 PUFAs; generally attenuate low-grade inflammation and are linked to lower atherosclerosis/CVD risk.	Reported to counter mitochondrial and ER stress, suppress NLRP3 activity in insulin-sensitive tissues, improve lipid handling and adipokine balance, and reduce JNK/other serine kinases that impede InsRec signaling; overall direction is IR-protective in most experimental frameworks; omega-3–rich diets (fish oil; selected plant oils) are broadly considered protective for IR, although magnitude depends on context and formulation.
**IL-1 receptor antagonist (IL-1RA)** [464,465,466,467,468,469]	Competitive inhibitor of IL-1 receptor signaling; major negative-feedback regulator that often rises in parallel with pro-inflammatory cytokines.	Circulating IL-1RA increases in morbid obesity/T2DM; in islets, IL-1RA can protect β-cells; extremely high IL-1RA concentrations may promote IR via mechanisms not strictly dependent on IL-1R1 binding (model-specific); pharmacologic IL-1R blockade in T2DM (recombinant IL-1RA) improves glycemia in interventional settings.
**IL-10** [470,471,472,473,474,475,476]	Canonical anti-inflammatory cytokine; limits NF-κB/MAPK activity, IL-1R expression, cytokine production and M1 polarization; IL-10R signals via JAK1/TYK2 → STAT3; produced by macrophage and T-cell subsets.	Tissue-specific effects are bidirectional in models: muscle/liver IL-10 overexpression protects mice from IR, whereas Treg-specific IL-10 loss lowered adipocyte IR and obesity under HFD; in humans, adipose macrophages may upregulate IL-10 in obesity, yet IL-10 does not directly alter human adipocyte function; circulating IL-10 correlates positively with insulin sensitivity; hyperglycemia can reduce IL-10R expression and STAT3 signaling in human macrophages; overall therapeutic positioning remains context-dependent.
**IL-11** [477,478,479,480,481,482,483,484,485,486]	IL-6 family member; broadly expressed IL-11R; conditionally anti-inflammatory (can limit NF-κB/cytokines) but also promotes adipogenesis, angiogenesis/vascular remodeling and fibrosis during chronic inflammation (linking chronicity and oncogenic/fibrotic programs); signals via JAK/STAT3, PI3K/AKT and RAS/ERK.	Findings are contradictory: some studies suggest IL-11 fosters meta-inflammation/inflamm-aging and chronic CVD progression; IL-11 deletion in mice can increase obesity and glucose intolerance (while affecting bone adaptation); in periodontitis + T2DM, lower IL-11 correlates with higher glucose; in rat T2DM models glucotoxicity upregulates IL-6/IL-11 with potential islet fibrosis, yet exogenous IL-11 lowered glucose in diabetic mice; net effect on IR is context-dependent.
**TGF-β (1–3)** [487,488,489,490,491,492,493]	Produced by many cells (macrophages, Tregs, activated platelets prominent); restrains proliferation and promotes differentiation; conditionally anti-inflammatory (limits cytokines and immune activation) but pro-fibrotic; can stimulate IL-11; engages MAPKs (JNK, p38) alongside canonical signaling.	Insulin enhances surface delivery of TGF-β receptors via PI3K/AKT; TGF-β can restrain adipose macrophage activation in T2DM and may support β-cell mass/function in some reports; however, it promotes organ fibrosis in T2DM; high TGF-β1 associates with obesity in rodents and humans, and systemic TGF-β blockade protects mice from obesity/diabetes/hepatic steatosis; overall effects on IR/T2DM are conflicting and tissue-/stage-dependent.
**IL-27** [494,495,496,497]	Pleiotropic cytokine balancing protective immunity and prevention of excessive inflammation; promotes type-1 programs (Th1/CTL/M1 via IFN-γ) yet supports Treg function and IL-10, and inhibits Th2/Th17; limits CTL cytolysis without suppressing cytokine output; antitumor effects context-dependent.	Often reported as metabolically protective in mice: enhances brown-fat thermogenesis, protects against diet-induced obesity and counteracts IR; increases glucose uptake in white adipocytes and lowers FA release; can activate JAK2/STAT3 and reduce OS/foam cells, suppress atherosclerosis and limit myocardial injury; however, by promoting Th1/CTL maturation it may contribute to β-cell damage/diabetes in some contexts; clinical associations are mixed (e.g., retinopathy aqueous IL-27 correlating with glycemia/lipids; IL-27R knockout reducing vascular inflammatory infiltration in aneurysm models); net effect on IR is context-dependent.
**IL-35** [498,499,500,501]	IL-12 family cytokine secreted prominently by Tregs; immunosuppressive/anti-inflammatory; inhibits pro-inflammatory cells/cytokines, increases IL-10 and TGF-β, and expands Tregs and Bregs.	In type 1 diabetes models, improves glycemia and protects islets (↓M1 function, ↓T-cell proliferation, ↓Th17, ↑Tregs); may protect against CVD (atherosclerosis, myocarditis) in experimental frameworks; roles in morbid obesity and T2DM remain insufficiently characterized.
**IL-37** [502,503,504,505,506,507,508,509,510]	Broad anti-inflammatory IL-1 family cytokine; intracellularly processed by caspase-1 and signals via Smad3 to suppress inflammatory programs; extracellularly forms IL-18Rα/IL-1R8 complex to inhibit NF-κB, AP-1 and MAPKs; highly expressed in inflammatory disease and proposed as a disease marker.	Improves IR in mice and humans and modulates low-grade adipose inflammation; higher IL-37 associates with better insulin responsiveness to therapy and less severe dysbiosis; IL-37 overexpression suppresses dysbiosis and diabetes development in models; benefits are also reported for atherosclerosis and inflamm-aging; IL-37 can be elevated in T2DM, consistent with a compensatory (limiting) rather than curative role during ongoing inflammation.
**IL-38** [511,512,513,514,515,516,517,518,519,520]	IL-1 family member with pronounced anti-inflammatory properties; produced by epithelial cells, monocytes/macrophages, fibroblasts and lymphoid cells; inhibits IL-1R1, IL-36R and IL-1RAPL1, thereby restraining MAPK/JNK and NF-κB signaling; in health, IL-38 correlates negatively with CRP, IL-6 and IL-1RA.	Reported to improve IR in multiple experimental settings: mitigates IRS-1/Akt signaling defects, increases glucose uptake, and upregulates PPARδ/SIRT1/antioxidant programs in palmitate-treated myocytes; in palmitate-exposed hepatocytes, reduces lipid accumulation and ER-stress markers while enhancing AMPK/autophagy; circulating IL-38 may rise in CVD (including atherosclerosis); human IL-38 levels in T2DM vary across studies, but anti-inflammatory and IR-lowering actions are supported experimentally.

Note. ALX, lipoxin A4 receptor; AMPK, Amp-Activated Protein Kinase; AP-1—Activator Protein-1; Breg, Regulatory B cell; CRP, C-reactive Protein; CTL, Cytotoxic T Lymphocytes; ER, Endoplasmic Reticulum; ERK, Extracellular Signal–Regulated Protein Kinase; FA, Fatty Acids; FPR2, N-formyl peptide receptor 2; GLUT4, Glucose Transporter Type 4; GPCRs, G Protein-Coupled Receptors; GPCRs, G Protein-Coupled Receptors; HbA1c, Glycated hemoglobin; IFN, Interferon; IL, Interleukin; IL-1RA, Interleukin-1 Receptor Antagonist; IL-1RAPL1, Interleukin-1 Receptor Accessory Protein-Like 1; InsRec, Insulin Receptor; IR, Insulin Resistance; IRS, Insulin Receptor Substrate; JAK, Janus Kinase; JNK, c-Jun N-terminal Kinase; LX, Lipoxin; M1, Classically Activated Macrophages; MAPK, Mitogen-Activated Protein Kinase; NF-κB, Nuclear Factor kappaB; NK, Natural Killer; NLRP3, Nucleotide-Binding Oligomerization Domain-Like Receptor Family Protein 3; PI3K, Phosphoinositide 3-Kinases; PPAR, Peroxisome Proliferator-Activated Receptor; RAS, Rat Sarcoma Viral Oncogene homolog; SIRT1, Sirtuin 1; SPMs, Specialized Proresolving Mediators; STAT, Signal Transduction and Activator of Transcription; T2DM, type 2 diabetes mellitus; TGF-β, Transforming Growth Factor beta; Th, T-helper; TNF, Tumor Necrosis Factor; Treg, CD4+ Regulatory T Cell; TYK, Tyrosine kinase; VFA, Visceral Fat Area; WC, Waist Circumference.

**Table 5:** The Role of PPARs in the Development of Insulin Resistance and Inflammation.

Measures/ Aspects	PPARα	PPARβ/δ	PPARγ
**Principal references**	[527,528,529,530,531,532,533,534,535,536,537,538,539,540]	[535,541,542,543,544,545,546,547,548]	[539,549,550,551,552,553,554,555,556,557,558]
**Predominant localization of PPARs**	Liver, skeletal muscle, adipose tissue, β-cells, kidneys, intestinal mucosa, heart; lower levels in other tissues.	Present in all human tissues at moderate levels; abundant in liver, skeletal muscle, heart, adipocytes, and macrophages.	PPARγ1: liver, skeletal muscle, β-cells, intestinal mucosa, immune cells. PPARγ2: exclusively in adipose tissue.
**Principal ligands**	Metabolites of glucose and fatty acids (FAs).	FAs, including omega-3 FAs; FA derivatives; oxidized lipids.	FAs and oxidized/nitrated FAs.
**Regulation of metabolism by PPARs**	Regulates genes for FA transport and β-oxidation. In liver, controls apolipoprotein expression for lipid-transport particles. Increases hepatocyte and adipocyte sensitivity to insulin’s metabolic effects.	In skeletal muscle, promotes oxidative metabolism of FAs and glucose. Activation yields a greater number of smaller adipocytes with improved adipokine profile and insulin sensitivity. In hepatocytes, activation improves (reduces) atherogenic dyslipidemia.	Directly regulates GLUT4 expression. Promotes lipogenesis and formation of small insulin-sensitive adipocytes. PPARγ-knockout mice cannot develop adipose tissue. PPARγ1 supports hepatic lipid synthesis (not in human MASLD/NAFLD) and stimulates hepatic glucose utilization. Lowers circulating FFA.
**Links between PPARs and insulin**	High glucose exposure in β-cells rapidly reduces PPARα gene expression, lowering FA β-oxidation and thereby increasing insulin production in β-cells. Thus, systemic and islet PPARα effects can influence β-cell insulin output.	In skeletal muscle, enhances basal and insulin-stimulated glucose uptake; promotes adipocyte differentiation toward an insulin-sensitive phenotype. Intestinal PPARβ/δ activation boosts GLP-1 secretion, preserving β-cell morphology and function.	Modulates insulin signaling by altering expression and/or phosphorylation of specific signaling molecules. Indirectly protects islets from lipotoxicity; PPARγ activation may directly improve β-cell function.
**Effects on pro-inflammatory cellular and tissue stress**	Suppresses pro-inflammatory signaling and limits hepatocyte ferroptosis. Lowers TNF-α, IL-1β, IL-6 via inhibition of AP-1 and NF-κB; AMPK (activated during ATP deficiency) upregulates PPARα. Activates PPARα and PKC, inhibits JNK. Increases IL-1RA, inhibits NLRP3 inflammasome and NF-κB pathways; promotes macrophage polarization toward M2.	Functionally linked to PKCα; activates AMPK. In adipocytes, inhibits expression and secretion of cytokines that activate NF-κB; prevents NLRP3 activation. Regulates vascular function by increasing VEGFR expression, Akt phosphorylation, and eNOS activity, thereby enhancing endothelial NO and counteracting hypertension. Reduces ROS and oxidative stress; promotes macrophage M2 polarization.	Activates AMPK; induces apoptosis of large insulin-resistant adipocytes; reduces production of adiponectin, resistin, IL-6, TNF-α (context-dependent reporting in source). Prevents NF-κB/iNOS-driven inflammatory cascades and lowers TNF-α, IL-1β, IFN-γ, IL-2, IL-18, IL-6, ROS; limits NLRP3 activation. Directly modulates several antioxidant genes in response to oxidative stress; enhances anti-inflammatory potential of adipose-tissue M2 macrophages.
**Influence of inflammatory/stress factors on PPAR activity**	NF-κB–driven cytokines (TNF-α, IL-1β, IL-6) downregulate PPARα; the antioxidant TF NRF2 can activate PPARα.	LPS and oxidized lipids stimulate PPARβ/δ. Anti-inflammatory effects are associated with moderate—but not strong—PPARβ/δ activation.	MAPKs (ERK, p38, JNK) phosphorylate and inhibit PPARγ. Under oxidative stress, the antioxidant TF NRF2 activates PPARγ.
**Role in IR-related and metabolic diseases**	PPARα reduction is reported across diseases, including MASLD/NAFLD, T2DM, Alzheimer’s disease, and cardiovascular disease. Altered PPARα function affects plasma lipids in T2DM—but not in healthy individuals—implicating PPARα as a link between diabetes and dyslipidemia. Endothelial PPARα deficiency promotes endotheliosis and atherosclerosis. PPARα knockout fosters IR in mice. All PPAR types may support metabolism in cancer cells.	Reduced PPARβ/δ activity in muscle, liver, and adipose tissue is associated with obesity, dyslipidemia, T2DM, and MASLD/NAFLD. Intestinal PPARβ/δ protects against diet-induced obesity. PPARβ/δ prevents lysosomal degradation of the insulin-receptor β subunit, thereby countering IR and T2DM. Activation impedes endotheliosis and atherosclerotic lesion formation, but PPARβ/δ is also implicated in carcinogenesis.	PPARγ activation in T2DM markedly improves insulin and glucose parameters by enhancing whole-body insulin sensitivity. PPARγ participates in numerous diseases (cancer, CVD, neurologic, renal, musculoskeletal, and metabolic). In the brain, PPARγ is protective against inflammation and oxidative stress; PPARγ agonists are proposed to have antidepressant effects.
**Pharmacology of PPAR agonists**	Several PPARα-selective agonists have been used to treat metabolic syndrome and T2DM, aiming to lower systemic IR. Nonselective PPAR agonists may be useful in neurodegenerative diseases.	Most antidiabetic effects of PPARβ/δ agonists (e.g., ASP0367, ASP1128, MBX-8025, REN-001) involve PPARβ/δ/AMPK activation, including upregulated glucose uptake, muscle remodeling, enhanced FA oxidation and autophagy, and inhibition of cellular stress and inflammation.	Ligand activation of PPARγ in T2DM improves insulin sensitivity and lowers plasma insulin and glucose. PPARγ agonists are also used against oxidative-stress–related disorders, including neurodegenerative and vascular diseases.

Note. AMPK, AMP-Activated Protein Kinase; AP-1, Activator Protein-1; ATP, Adenosine-5′-Triphosphate; eNOS, Endothelial Nitric Oxide Synthase; ERK, Extracellular Signal–Regulated Protein Kinase; FA, Fatty Acids; FFAs, Free Fatty Acids; GLP-1, Glucagon-Like Peptide-1; GLUT4, Glucose Transporter Type 4; IFN, Interferon; IL, Interleukin; IL-1RA, Interleukin-1 Receptor Antagonist; IR, Insulin Resistance; JNK, c-Jun N-terminal Kinase; LPS, Lipopolysaccharide (Endotoxin) of Gram-Negative Bacteria; M2, Alternatively Activated Macrophages; MASLD, Metabolically Associated Steatotic Liver Disease; NAFLD, Non-Alcoholic Fatty Liver Disease; NF-κB, Nuclear Factor kappa B; NLRP3, Nucleotide-Binding Oligomerization Domain-Like Receptor Family Protein 3; PKC, Protein Kinase C; PPARs, Peroxisome Proliferator-Activated Receptors; ROS, Reactive Oxygen Species; T2DM, Type 2 Diabetes Mellitus; TNF, Tumor Necrosis Factor; VEGF, Vascular Endothelial Growth Factor.

**Table 6:** Characteristics of Human Sodium–Glucose Cotransporters (SGLT) [563,564,565,566,567].

Transporter	Localization	Function
**SGLT1**	Small-intestinal epithelium (apical membrane); renal proximal tubule S3 segment	Intestinal glucose absorption; renal reabsorption of remaining filtered glucose (late proximal tubule).
**SGLT2**	Renal proximal convoluted tubule (S1–S2)	Reabsorbs bulk of filtered glucose; contributes to hyperglycemia in diabetes via increased renal glucose reclamation.
**SGLT3**	Intestine; testes; uterus; lungs; brain; thyroid	Glucose sensor (non-canonical transporter role) contributing to glucose-level regulation (notably intestine/brain).
**SGLT4**	Intestine; kidney; liver; brain; lungs; uterus; pancreas	Uptake/reabsorption of mannose and fructose; may also transport glucose (context-dependent).
**SGLT5**	Renal cortex	Transport of glucose and galactose (renal handling).
**SGLT6**	Brain; kidney; intestine	Predominantly inositol transport.

Note. SGLT, Sodium–Glucose co-Transporter.

**Table 7:** Characteristics of Human GLUT-Type Glucose Transporters.

Transporters (Class)	Localization	Functions	Dysfunctions/Disease Associations
**GLUT1 (I)** [563,564,565,566,567,568,569,570,571]	Embryonic tissues; endothelium; erythrocytes; brain (neurons/glia); immune cells (T cells; macrophages); β-cells (human); hepatocytes/adipocytes (variable).	Basal glucose transport; key glucose sensor (Km ~1.5–3.5 mM); high expression in embryonic/proliferative cells; in adults, prominent in CNS and barrier endothelium.	GLUT1 deficiency syndrome (SLC2A1): epileptic encephalopathy/developmental delay/microcephaly; upregulated in multiple tumors (HIF-1–linked); altered hepatic expression reported in steatotic liver disease and HCC; vascular GLUT1 used as barrier/placental marker in diagnostics.
**GLUT2 (I)** [564,565,566,567,568,569,570,571,572,573,574]	Hepatocytes; intestinal and renal tubular epithelium; pancreatic β-cells; select immune/brain cells.	Low-affinity/high-capacity sensor under hyperglycemia (Km ~15–20 mM); epithelial glucose export to blood; dominant hepatocyte transporter with bidirectional flux; also transports galactose/mannose/fructose/glucosamine.	Fanconi–Bickel syndrome (SLC2A2): glycogen hepatomegaly; glucose/galactose intolerance; fasting hypoglycemia; renal tubulopathy; growth impairment; polymorphisms associated with fasting hyperglycemia/T2DM risk traits; hepatic fructose influx via GLUT2 linked to de novo lipogenesis and steatosis in some frameworks.
**GLUT3 (I)** [564,565,566,567,568,569,570,571,572,573,574,575]	Enriched in neurons; also leukocytes/macrophages, platelets, fibroblasts, placenta.	High-affinity neuronal uptake (Km ~1–2 mM); complements BBB delivery (GLUT1) by governing neuronal import; vesicular pool translocates upon activation to augment uptake.	Upregulated in cancers (similar to GLUT1); increased hepatic expression associated with MASLD/NAFLD in reports; functional impairment proposed in select immune dysfunction contexts (heterogeneous evidence).
**GLUT4 (I)** [164,165,166,167,168,169,170,171,172,173,174,175,176,177,178,179,180,181,182,183,184,185,186,187,188,189,190,191,192,193,194,195,196,197,198,199,200,201,202,203,204,205,206,207,208,209,210,211,212,213,214,215,216,217,218,219,220,221,222,223,224,225,226,227,228,229,230,231,232,233,234,235,236,237,238,239,240,241,242,243,244,245,246,247,248,249,250,251,252,253,254,255,256,257,258,259,260,261,262,263,264,265,266,267,268,269,270,271,272,273,274,275,276,277,278,279,280,281,282,283,284,285,286,287,288,289,290,291,292,293,294,295,296,297,298,299,300,301,302,303,304,305,306,307,308,309,310,311,312,313,314,315,316,317,318,319,320,321,322,323,324,325,326,327,328,329,330,331,332,333,334,335,336,337,338,339,340,341,342,343,344,345,346,347,348,349,350,351,352,353,354,355,356,357,358,359,360,361,362,363,364,365,366,367,368,369,370,371,372,373,374,375,376,377,378,379,380,381,382,383,384,385,386,387,388,389,390,391,392,393,394,395,396,397,398,399,400,401,402,403,404,405,406,407,408,409,410,411,412,413,414,415,416,417,418,419,420,421,422,423,424,425,426,427,428,429,430,431,432,433,434,435,436,437,438,439,440,441,442,443,444,445,446,447,448,449,450,451,452,453,454,455,456,457,458,459,460,461,462,463,464,465,466,467,468,469,470,471,472,473,474,475,476,477,478,479,480,481,482,483,484,485,486,487,488,489,490,491,492,493,494,495,496,497,498,499,500,501,502,503,504,505,506,507,508,509,510,511,512,513,514,515,516,517,518,519,520,521,522,523,524,525,526,527,528,529,530,531,532,533,534,535,536,537,538,539,540,541,542,543,544,545,546,547,548,549,550,551,552,553,554,555,556,557,558,559,560,561,562,563,564,565,566,567,568,569,570,571,572,573,574,575,576,577,578]	Skeletal muscle; adipocytes; heart; select brain regions (hippocampus/hypothalamus).	Main insulin-stimulated glucose uptake pathway in muscle/adipose (Km ~5 mM); insulin-driven vesicle translocation to membrane; contraction increases muscle GLUT4 independently of insulin; also transports selected hexoses/derivatives.	Reduced GLUT4 abundance/translocation is a core feature of IR and T2DM; adipose GLUT4 is often more reduced than muscle; diminished membrane GLUT4 in large adipocytes aligns with insulin-refractory phenotype; impaired hippocampal GLUT4 translocation reported in early AD contexts.
**GLUT14 (I)** [564,565,566,567,568,569,570,571,572,573,574,575,576,577,578,579,580]	Testis; intestine.	Putative transporter (glucose; dehydroascorbic acid).	Expression reported in gastric adenocarcinoma with possible prognostic relevance (often discussed with GLUT1).
**GLUT5 (II)** [564,565,566,567,568,569,570,571]	Small-intestinal epithelium (high); lower in testis, muscle, kidney, adipose, liver, brain.	Preferential fructose transporter; essential for intestinal fructose absorption.	Interest driven by associations between high fructose exposure and obesity/T2DM/MASLD; upregulated in tumors in some settings (association-level evidence).
**GLUT7 (II)** [564,565,566,567,568,569,570,571]	Small and large intestine; testis; prostate.	Very high affinity for glucose and fructose (Km < 0.5 mM).	Linked to gastrointestinal disease phenotypes in limited literature (heterogeneous).
**GLUT9 (II)** [564,565,566,567,568,569,570,571,572,573,574,575,576,577,578,579,580,581]	Proximal renal tubules (dominant); liver; placenta; intestine; immune cells; chondrocytes.	High affinity for glucose/fructose/urate (Km ~0.5 mM); high-capacity urate transporter shaping systemic urate via hepatic handling and renal reabsorption.	Loss-of-function mutations cause renal urate wasting and hypouricemia; liver-specific inactivation can induce hyperuricemia without other abnormalities (supports GLUT9 as gout-relevant target).
**GLUT11 (II)** [564,565,566,567,568,569,570,571,572,573,574,575,576,577,578,579,580,581,582]	Isoforms: A (heart/muscle/kidney); B (placenta/adipose/kidney); C (adipose/heart/muscle/pancreas).	Glucose/fructose transport, especially in muscle; localized to slow-twitch human skeletal fibers; absent in rodents.	GLUT11 (with GLUT8) reported as proliferation/viability factor in multiple myeloma models (therapeutic vulnerability hypothesis).
**GLUT6 (III)** [564,565,566,567,568,569,570,571]	Brain and spleen; peripheral leukocytes; inflammatory endothelial cells (high).	Low-affinity intracellular transporter (glucose/fructose); likely supports intracellular carbohydrate handling in immune/inflammatory contexts.	Proposed glycolysis modulator in inflammatory macrophages; knockout mice show minimal whole-body phenotype (suggesting context-specific roles).
**GLUT8 (III)** [564,565,566,567,568,569,570,571]	Brain; testis/sperm; hepatocytes; adrenal; endometrium; brown adipose.	High-affinity intracellular transporter (Km ~2 mM); translocates after insulin stimulation; transports glucose/galactose/fructose across intracellular membranes (mitochondria/ER/lysosomes).	Knockout viable with mild phenotypes; hepatic GLUT8 hyperfunction linked to IR in some models; GLUT8 deficiency may attenuate fructose-driven metabolic changes, including steatosis (context-dependent).
**GLUT10 (III)** [564,569,571,583]	Skeletal muscle; heart; lungs; brain; placenta; kidney; liver; adipose; pancreas.	Transports glucose/galactose; mitochondrial dehydroascorbic acid transport proposed → oxidative stress buffering; expression stimulated by unsaturated FFAs and inhibited by saturated FFAs (reported).	Mutations cause arterial tortuosity syndrome; mitochondrial dysfunction described in knockout settings (mechanisms not fully resolved).
**GLUT12 (III)** [564,569,571]	Adipose; small intestine; skeletal muscle; heart; placenta; prostate; kidney; chondrocytes.	In cardiomyocytes, surface expression may be insulin-independent, consistent with basal transport role; broader tissue roles under study.	Considered a therapeutic avenue in oncology/neurodegeneration discussions (association-level; evolving evidence).
**GLUT13 (III)** [564,569,571]	Brain (hippocampus/hypothalamus/cerebellum/brainstem); lower in adipose and kidney.	Transports inositol and inositol-3-phosphate; predominantly intracellular; translocates upon neuronal depolarization.	Dysregulation of inositol-3-phosphate transport linked to psychiatric disorders in some frameworks (heterogeneous evidence).

Note. CNS, Central Nervous System; FFA, Free Fatty Acid; GLUT, Glucose Transporter; HCV, Hepatitis C Virus; HIF-1, Hypoxia-Inducible Factor 1; MASLD, Metabolically Associated Steatotic Liver Disease; NAFLD, Non-Alcoholic Fatty Liver Disease; T2DM, Type 2 Diabetes Mellitus.

**Table 8:** General characteristics of fatty acid–binding proteins (FABPs).

FABP	Localization	Function	Association with Pathology
**FABP1 (L-FABP)** [597,598,599,600]	Liver (hepatocytes); also intestine, pancreas, kidney, lung, stomach	Uptake and intracellular trafficking of LCFAs and related hydrophobic ligands (oxidized FAs, acyl-CoA, bile acids, cholesterol, heme, endocannabinoids, steroid hormones, vitamin D, lysophospholipids; drug ligands incl. fibrates); supports FA metabolism and HDL → BA conversion; nuclear activation of PPARα.	Modulates hepatic endocannabinoid axis in MASLD/NAFLD; T94A variant and/or higher FABP1 levels associated with obesity, dyslipidemia, thrombosis and MASLD/NAFLD; pharmacologic inhibition may reduce hepatic lipid accumulation, yet depletion can aggravate steatosis toward steatohepatitis (bidirectional); circulating FABP1 increases in acute/chronic hepatocellular injury and cirrhosis; dysregulated expression (up or down) may predispose to pathology.
**FABP2 (I-FABP)** [597,598,601,602,603]	Intestinal epithelium; (reported also in liver)	Preferential LCFA binding; facilitates uptake/trafficking and intracellular metabolism of dietary LCFAs; supports TG-rich lipoprotein assembly; proposed lipid-sensing role; may ferry lipophilic drugs in intestine.	Soluble FABP2 (sFABP2) rises as biomarker of enterocyte injury/death; reported paradoxical decrease in COVID-19 (reflecting functional alteration of enterocytes rather than frank injury; proposed link to hypolipidemia); Ala54Thr polymorphism linked to overweight risk and higher T2DM susceptibility in some populations (notably Asian cohorts; weaker/absent in Europeans).
**FABP3 (H-FABP)** [597,598,604,605,606,607,608,609]	Heart and skeletal muscle; also brain, kidney, lung, stomach, testes, adrenals, mammary gland, placenta, ovaries, brown adipose; lymphocytes/macrophages; endothelium	LCFA/eicosanoid/retinoid binding within FABP3/4/5/7/8/9 subfamily; channels n-6 PUFAs into phospholipids; shuttles FAs from membrane to mitochondria for β-oxidation; nuclear PPAR activation; binds long-chain acylcarnitines and may buffer their lipotoxicity; reported immunomodulatory role (B-cell programs via Blimp-1).	Endogenous FABP3 can amplify LPS-induced endothelial dysfunction (pro-inflammatory signaling); high tumor FABP3 expression correlates with poorer survival (association); implicated in dopaminergic control and Parkinson’s disease; conversely may limit foam-cell formation via PPARγ-linked mechanisms (potentially anti-atherogenic); circulating sFABP3 increases with muscle injury and is used as early MI marker; explored as vascular/Alzheimer’s biomarker (heterogeneous evidence).
**FABP4 (A-FABP)** [597,598,610,611,612,613,614,615,616]	Adipocytes; macrophages/monocytes/DCs; skeletal muscle; cardiomyocytes; endothelium; placenta; cancer cells	Central adipocyte/macrophage lipid chaperone; regulates FA metabolism and lipolysis signaling; interacts with PPARγ programs (reported promotion of PPARγ degradation in adipocytes, while nuclear FABP4 can activate PPARγ—context-dependent); secreted sFABP4 acts as adipokine; described extracellular complex with ADK/NDPK shaping extracellular adenosine/ATP/ADP signaling (purinergic stress axis).	Strongly associated with IR/obesity/T2DM and vascular pathology (endothelial dysfunction/endotheliosis, atherosclerosis, HF); macrophage FABP4 linked to inflammation and cholesterol loading; high sFABP4 associates with metabolic derangement and HF; pharmacologic blockade proposed for cardiometabolic disease; disruption of sFABP4–ADK–NDPK axis reported to preserve β-cell mass/function and protect from diabetes in models; GWAS and observational data implicate FABP4 as CHD/T2DM risk signal; also linked to hepatic gluconeogenic enzyme activation and tumor progression/adipose–tumor crosstalk (evidence type varies by claim).
**FABP5 (E-FABP)** [597,598,617,618,619]	Skin and stratified epithelia; adipocytes; macrophages/DCs/lymphocytes; mammary gland; brain; GI tract; kidney/liver/lung/heart/skeletal muscle; testis; retina/lens; spleen; placenta; endothelium	LCFA chaperone with broad tissue distribution; directs lipids to intracellular compartments; nuclear co-factor for PPARγ activation; shapes FA uptake/oxidation and T-cell subset survival/function.	Implicated in obesity/IR/T2DM; dermatologic inflammation (e.g., psoriasis); neurodegeneration (e.g., Alzheimer’s); multiple cancers (especially colorectal/prostate/breast); dual FABP4/5 inhibition proposed to reduce atherosclerotic risk and improve glucose homeostasis while limiting tumor growth/metastasis (mostly preclinical/early translational framing).
**FABP6 (ileal FABP)** [597,598,620,621,622]	Ileum (dominant); also ovary, adrenals, stomach	Higher affinity for bile acids than for FAs; mediates BA reabsorption and intracellular BA transport; also transports LCFAs/acyl-CoA; nuclear activation of PPARγ.	Linked to colorectal cancer and T2DM; FABP6 inhibition reported to suppress bladder-cancer growth via autophagy activation and PI3K/AKT/mTOR and PI3K/AKT/p53 pathway modulation (model-based); considered potential antidiabetic target; Thr79Met polymorphism reported to confer protection from T2DM in individuals with obesity.
**FABP7 (B-FABP)** [597,598,623,624,625,626]	CNS neurons/glia; retina; mammary gland	High affinity for n-3 PUFAs; enriched in embryonic brain; regulates neurogenesis and glial proliferation; proposed mediator of leptin effects in hypothalamic arcuate nucleus.	Increased expression in low-grade neuroinflammation and neurodegeneration; FABP7 mRNA elevated in postmortem brains in ASD and schizophrenia cohorts and linked to Down syndrome/schizophrenia (associations); markedly overexpressed in several cancers with poor prognosis; suppression improves survival in models (preclinical).
**FABP8** [597,627]	Peripheral nervous system; Schwann cells	Binds LCFAs and sphingomyelin; promotes sphingomyelin trans-bilayer movement (outer → inner leaflet).	Gene mutations associated with myelin degeneration and demyelinating neuropathies.
**FABP9** [597,598,628,629]	Testis (high); also salivary and mammary glands	FA/hydrophobic ligand binding; testis-enriched expression.	Strong expression reported in prostate cancer cells (association).

Note. ADK, Adenosine Kinase; ADP, Adenosine-5′-Diphosphate; ATP, Adenosine-5′-Triphosphate; BAs, Bile Acids; FA, Fatty Acids; FABP, Fatty Acid Binding Proteins; IR, Insulin Resistance; LCFA, Long-Chain Fatty Acids; LPS, Lipopolysaccharide (Endotoxin) of Gram-Negative Bacteria; MASLD, Metabolically Associated Steatotic Liver Disease; NAFLD, Non-Alcoholic Fatty Liver Disease; NDPK, Nucleoside Diphosphate Kinases; PPARs, Peroxisome Proliferator-Activated Receptors; T2DM, Type 2 Diabetes Mellitus.

**Table 9:** Metabolic and Pro-inflammatory Functions of Transmembrane Fatty-acid Transporters.

Transporter	Principal Localization (Concise)	Key Functions and Roles in Pathology (Concise; Non-Universal Causality)
**FABPpm** [638,639,640,641]	Skeletal muscle; cardiomyocytes; hepatocytes; adipocytes; small-intestinal epithelium; placenta	Major contributor (with CD36) to LCFA/unsaturated FA uptake in muscle and likely other insulin-responsive tissues; overexpression increases LCFA transport and metabolism (model-based); transmembrane transport mechanism remains incompletely resolved.
**FATP1 (SLC27A1)** [640,642,643,644,645,646,647,648,649,650,651]	Adipocytes; skeletal muscle; endothelium; astrocytes/neurons; intestinal epithelium; cancer cells; lipid-associated M2 macrophages (LAM)	Insulin-stimulated LCFA uptake via translocation of FATP1 to plasma membrane (adipocytes/muscle); FATP1-null models: insulin-stimulated uptake markedly reduced while basal uptake largely preserved (tissue- and model-dependent); increased LCFA influx may favor intramyocellular lipid accumulation and contribute to IR if oxidation is insufficient; human SLC27A1 variants linked to metabolic disturbances (IR/T2DM risk traits); macrophage FATP1 can reduce glucose use and pro-inflammatory activity (context-dependent); supports LCFA trafficking at BBB and LCFA delivery to muscle mitochondria (with FATP4/CD36); competes with ketone-body use by muscle; proposed signaling roles via GPCR-related pathways; reported suppression of bone-marrow CD8 T-cell function in specific settings; elevated expression in breast cancer (association).
**FATP2 (SLC27A2)** [652,653,654,655,656,657,658]	Hepatocytes; renal and intestinal epithelium; macrophages (incl. osteoclast lineage); placenta	Substantial component of hepatic LCFA uptake (~40% in cited framework); implicated in MASLD/NAFLD and T2DM; hyperfunction linked to lipotoxicity, ROS and pro-inflammatory cellular stress; supports lipid metabolism and osteoclast differentiation; implicated in diabetic kidney disease fibrosis phenotypes; FATP2 deficiency in mice associated with reduced IR and glycemia plus β-cell hyperplasia/sustained insulin secretion (model-based).
**FATP3 (SLC27A3)** [659,660,661,662]	Heart; muscle; adipose; lung; kidney; pancreas; vascular endothelium; leukocytes; placenta; cancer cells	Works with FATP4 and CD36 in trans-endothelial LCFA transport and LCFA activation (acyl-CoA formation); endothelial expression reportedly VEGF-B–driven; diabetes-related CVD: blood DNA hypermethylation signals for FATP3/4 reported as potential diagnostic markers (association-level evidence).
**FATP4 (SLC27A4)** [644,663,664,665,666,667,668]	Cardiomyocytes; skeletal muscle; hepatocytes; adipocytes; keratinocytes; endothelium; leukocytes; astrocytes/neurons; enterocytes; placenta	Broad LCFA transporter involved in basal and insulin-modulated uptake; in liver, secondary to FATP2/5 for bulk uptake but FA activation via FATP4 may favor hepatocellular lipid accumulation in obesity; adipose FATP4 expression elevated in obesity and, when increased, associated with IR phenotypes; TNF-α may reduce FATP4 via InsRec inhibition (one axis), yet TNF-α–linked NF-κB/autophagy programs can increase FATP4 in other contexts (bidirectional regulation—state explicitly); principal intestinal FA transporter; ER-resident protein with FA transport/activation roles; in muscle sarcolemma, prominent in FA activation for β-oxidation; FATP4 mutations: neonatal anomalies with survival into adulthood; adult phenotype includes hyperkeratosis plus immune/allergic features (eosinophilia); partial functional overlap with FATP1 (compensatory expression suggested).
**FATP5 (SLC27A5)** [669,670,671,672,673,674]	Hepatocytes; selected cancer types	In obesity/excess FA intake, hepatic CD36/FATP2/FATP5 upregulated (not in normal state); FATP5 activity linked to IR and MASLD/NAFLD; knockout reduces hepatic lipid uptake (~50% in cited framework); may suppress glucose uptake and glycolytic ATP in hepatocytes → potential AMPK activation and mTOR inhibition (mechanistic framing); tumor context differs: proposed growth-limiting axis in some hepatic/colorectal settings vs. growth-promoting in prostate cancer (direction depends on metabolic wiring/AMPK coupling); reported requirement for intrahepatic cholangiocarcinoma growth (context-specific).
**FATP6 (SLC27A6)** [675,676,677]	Heart (dominant); placenta	Human-specific cardiomyocyte-enriched transporter; FA β-oxidation supplies major fraction of cardiac energy; FATP6 hyperfunction proposed to shift glucose–FA utilization balance and increase CVD risk (mechanistic hypothesis); nonetheless, CD36 is the dominant contributor to myocardial LCFA transport in most frameworks.
**CD36 (FAT)** [644,678,679,680,681,682,683,684]	Hepatocytes; adipocytes; endothelium; vascular smooth muscle; cardiomyocytes; skeletal muscle; platelets/erythrocytes; lymphocyte subsets; macrophages; astrocytes/neurons; epithelia	Best-characterized LCFA transporter in skeletal muscle; insulin increases CD36 translocation to membrane in myocytes/adipocytes → higher FFA uptake; CD36 deficiency: elevated plasma FFA/TAG and lower glucose; phenotype includes dissociation between improved muscle insulin sensitivity and reduced hepatic insulin sensitivity (model-dependent); in humans with reduced CD36 expression: endothelial dysfunction linked to reduced Akt activity and NO bioavailability in microvascular endothelium; therapeutic concept: direct CD36 targeting or modulation of subcellular recycling; additional role as scavenger receptor.

Note. AMPK, AMP-Activated Protein Kinase; ATP, Adenosine-5′-Triphosphate; ER, Endoplasmic Reticulum; FA, Fatty Acids; FABP, Fatty Acid Binding Proteins; FAT, Fatty Acid Translocase; FATP, Fatty Acid Transporter Proteins; FFAs, Free Fatty Acids; InsRec, Insulin Receptor; IR, Insulin Resistance; LAM, Lipid Associated Macrophages; LCFA, Long-Chain Fatty Acids; MASLD, Metabolically Associated Steatotic Liver Disease; mTOR, Mammalian Target of Rapamycin; NAFLD, Non-Alcoholic Fatty Liver Disease; NF-κB, Nuclear Factor kappa B; T2DM, Type 2 Diabetes Mellitus; TNF, Tumor Necrosis Factor; VEGF, Vascular Endothelial Growth Factor.

**Table 10:** Sites of Production, Blood Changes, Metabolic Effects, and Inflammatory Links of Major Adipokines.

Adipokine	Primary Production Sites	Blood Change in Obesity & T2DM	Metabolic Effects	Links with Inflammation (Concise; Directionality Noted)
**Leptin** [691,692,693,694,695,696,697]	Mainly white-adipose adipocytes	↑	Generally improves insulin sensitivity in liver/muscle and modulates β-cell function; central leptin resistance develops in obesity, blunting anorexigenic/energy-expenditure effects.	Predominantly pro-inflammatory: promotes cytokine production and immune activation; LepR signals via JAK2/PI3K/Akt/MAPK and JAK2/STAT3; circulating leptin correlates with hepatic fibrosis severity (association).
**Resistin** [698,699,700,701]	Adipocytes; monocytes; astrocytes; intestinal epithelium; skeletal muscle; leukocytes	↑	Generally associated with increased IR; in humans, proposed to act via CB1R signaling in immune cells within adipose tissue (mechanistic model).	Pro-inflammatory: TLR4 ligand; can activate complement; promotes endothelial activation and transendothelial leukocyte migration.
**PAI-1** [702,703,704]	Adipocytes; endothelial cells; fibroblasts; macrophages	↑	Associated with increased IR; central inhibitor of plasminogen activator → prothrombotic milieu in T2DM; elevated levels track with obesity.	Pro-inflammatory amplifier: induced by inflammation and can sustain it; signals via LRP1 (CD91); reported shift toward JAK1/STAT1 and ERK pathways with restraint of PI3K/Akt (pathway-level description).
**Visfatin (NAMPT)** [705,706,707,708,709]	Predominantly visceral adipose; also multiple tissues	↑/↓ (variable)	Reported insulin-sensitizing/insulin-mimetic actions (β-cell support, glucose uptake, gluconeogenesis suppression), but net metabolic direction is heterogeneous across studies and contexts.	Often pro-inflammatory in experimental settings: stimulates cytokines, promotes atherogenic programs, can induce NLRP3; activates PI3K/AKT, MAPK/NF-κB and other pathways; may indirectly worsen IR via inflammation.
**RBP4** [710,711,712,713]	Hepatocytes (major circulating source); adipocytes (local effects)	↑	Generally increases IR: suppresses β-cell insulin secretion and reduces insulin-stimulated GLUT4 translocation in adipocytes/muscle; experimental elevation can induce systemic IR (model-based).	Pro-inflammatory: activates macrophage NLRP3 via TLR4/MD2; via TLR2 increases cytokines; promotes adipose immune infiltration and inflammatory impairment of adipocyte insulin signaling.
**Adiponectin** [714,715,716]	Adipocytes (higher in subcutaneous vs. visceral depots)	↓	Insulin-sensitizing: activates AMPK/PPARα via AdipoR1 (muscle) and AdipoR2 (liver); increases FA oxidation, glucose uptake and muscle GLUT4 translocation; reduces gluconeogenesis.	Anti-inflammatory/pro-resolving: lowers TNF-α/CRP/oxidative stress; inhibits TLR4/NF-κB and TNF-α/NF-κB; supports β-cell survival; deficiency linked to endothelial activation and worse systemic inflammatory outcomes (context).
**SFRP5** [717,718,719]	Adipocytes; also hepatocytes, skeletal muscle, β-cells	↓/↑ (variable)	Generally linked to improved insulin signaling via restraint of WNT5A/JNK1; proposed support of β-cell proliferation (model-based).	Anti-inflammatory: suppresses WNT5A/JNK1 signaling in macrophages and other adipose immune cells.
**WNT5A** [719]	Adipocytes; monocytes/macrophages	↑	Promotes IR: WNT5A/JNK1 inhibits IRS-1 and reduces insulin signaling (cell/tissue models).	Pro-inflammatory: mediator of innate immune activation; promotes oxidative/pro-inflammatory stress programs.
**ANGPTL2** [720,721,722]	Adipocytes; vascular/perivascular cells	↑	Associated with increased IR and reduced glucose tolerance; can act directly on adipocytes via CD146, supporting obesity-related metabolic impairment (experimental).	Pro-inflammatory: promotes recruitment/activation of macrophages and endothelium; signals via integrin α5β1/MAPK and NF-κB, supporting tissue remodeling and inflammation.
**Apelin** [723,724,725]	Adipocytes; endothelial cells; pancreatic α/β-cells; skeletal muscle; others	↑	Generally insulin-sensitizing: increases glucose uptake in muscle/adipocytes; may reduce β-cell insulin secretion; elevated apelin in IR often interpreted as compensatory; administration improves metabolic phenotypes in models.	Anti-inflammatory: APJ GPCR signaling restrains NF-κB; reported inhibition of NF-κB/JNK and activation of AMPK/GSK-3β/NRF2 axes (model-dependent).
**Vaspin (SERPINA12)** [726,727,728,729]	Adipocytes; also skin, liver, placenta, hypothalamus, pancreas	↑	Typically associated with improved insulin sensitivity/glucose tolerance; often interpreted as compensatory rise with obesity/IR; may reduce food intake in vivo (context).	Anti-inflammatory: inhibits NF-κB–dependent adhesion-molecule expression; can block leptin-induced inflammation; signals via GRP78 (HSPA5); anti-inflammatory adipokines (incl. vaspin/chemerin/omentin) reported reduced in severe COVID-19 (association).
**Chemerin** [730,731]	Adipocytes	↑	Mixed: in adipocytes can enhance insulin-stimulated glucose uptake via ChemR23/GPR1-related signaling; in primary human skeletal muscle cells can induce IR at IRS-1 level → tissue-specific divergence.	Predominantly pro-inflammatory: chemotactic for ChemR23-expressing leukocytes, supporting adipose immune infiltration and inflammation.
**Omentin-1** [732,733,734]	Visceral adipose adipocytes; endothelial cells	↓	Insulin-sensitizing: enhances PI3K/Akt signaling and improves insulin-mediated glucose transport in facultatively glycolytic tissues.	Anti-inflammatory: increases IL-10/adiponectin and reduces pro-inflammatory cytokine expression in adipose tissue and liver (context).

Note. AMPK, AMP-Activated Protein Kinase; ANGPTL2, Angiopoietin-Related Protein 2; APJ, G Protein-Coupled Receptor; CB1R, Cannabinoid Receptor 1; CNS, Central Nervous System; CRP, C-reactive Protein; ERK, Extracellular Signal–Regulated Protein Kinase; GLUT, Glucose Transporter; GRP, Glucose-Regulated Protein; GSK-3β, Glycogen Synthase Kinase-3beta; IL, Interleukin; IR, Insulin Resistance; IRS, Insulin Receptor Substrates; JAK, Janus Kinase; JNK, c-Jun N-terminal Kinase; LRP1, LDL Receptor-related Protein-1 (CD91); M1, Classically Activated Macrophages; MAPK, Mitogen-Activated Protein Kinase; MD2, Myeloid Differentiation Protein 2; NF-κB, Nuclear Factor kappa B; NLRP3, Nucleotide-Binding Oligomerization Domain-Like Receptor Family Protein 3; NRF2, Nuclear Factor Erythroid-2-Related Factor 2; PAI-1, Plasminogen Activation Inhibitor of the First Type; PI3K, Phosphoinositide 3-Kinases; PPAR, Peroxisome Proliferator-Activated Receptor; RBP4, Retinol Binding Protein 4; SFRP5, Secreted Frizzled-Related Protein 5; STAT, Signal Transduction and Activator of Transcription; T2DM, Type 2 Diabetes Mellitus; TLR, Toll-Like-Receptor; TNF, Tumor Necrosis Factor; WNT5A, a member of the Wnt protein family, which acts on classical or multiple non-classical Wnt signaling pathways by binding to different receptors.

**Table 11:** Sites of Production, Blood Changes, Metabolic/Homeostatic Effects, and Inflammatory Links of Major Hepatokines.

Hepatokine	Primary Production Site(s)	Blood Change in Obesity, T2DM, MASLD	Metabolic/Homeostatic Effects	Links with Inflammation and Pathology
**Fetuin-A (FetA)** [735,736,737,738,739]	Hepatocytes; adipose tissue	↑	Inhibits insulin-receptor tyrosine kinase activity and downstream signaling; linked to adipose/muscle IR and reduced β-cell maturation/insulin output; induced by FFA and glucose (lipid-driven IR axis).	Acts as endogenous TLR4 ligand/co-factor and can potentiate FFA–TLR4 signaling and M1 polarization; however, anti-inflammatory effects reported in sepsis/autoimmune contexts → bidirectional, context-dependent.
**FGF21** [735,736,737,738,739,740,741]	Mainly liver; also adipose, heart, skeletal muscle, pancreas	↑ (often compensatory)	Generally insulin-sensitizing: promotes hepatic FA β-oxidation, suppresses lipogenesis/gluconeogenesis, increases adipocyte glucose uptake; hypothalamic effects on feeding/weight; supports β-cell survival; “FGF21 resistance” can emerge with IR.	Predominantly anti-inflammatory/metabolic-stress hormone: induced by mitochondrial stress/ATF4; reported inhibition of NF-κB translocation in adipocytes; associated with protection against hyperglycemia, dyslipidemia, steatohepatitis; agonists under development for MASLD/NAFLD/T2DM.
**ANGPTL2** [720,735,736,742]	Liver; adipose tissue	Variable/insufficiently consistent	In adipose tissue, associated with worsened insulin sensitivity/IR (evidence mainly experimental/associative).	Pro-inflammatory vascular/adipose signaling: binds integrin α5β1, activates NF-κB, cytokines, adhesion molecules; associates with adipose macrophage accumulation, adipocyte ER stress and IR.
**ANGPTL3** [735,736,737,739,743,744]	Liver	↑	Inhibits lipoprotein lipase → ↑ TAG; can aggravate IR in adipose/muscle; hepatic gluconeogenesis reported; production suppressed by leptin/statins/thyroxine/insulin.	Can activate endothelium/angiogenesis via integrin αVβ3; promotes macrophage cytokine production; inhibitors are expected to be anti-atherogenic and anti-inflammatory (direction inferred from lipid-lowering and pathway data).
**ANGPTL4** [742,745,746]	Liver; adipose tissue	↑/↓ (variable)	Fasting-induced via PPARα; inhibits lipoprotein lipase → ↑ TAG; metabolic direction can diverge by tissue/state.	In mice, hepatocyte ANGPTL4 inhibition lowers TAG/cholesterol and protects against diet-induced obesity, glucose intolerance, steatosis and atherogenesis; can participate in canonical inflammation; net effect on IR appears context-dependent.
**ANGPTL6** [735,736,737,739]	Predominantly liver	↑	Enhances AMPK and insulin signaling in muscle/liver; suppresses gluconeogenesis; increases PPARα expression; angiogenic roles noted.	Inflammation links mixed; overexpression reported beneficial in obesity/MASLD and improves IR in models → net effects appear protective but not uniform across datasets.
**ANGPTL8** [735,736,737,739]	Liver; adipose tissue	Variable/insufficiently consistent	Proposed to improve glucose metabolism (downregulates gluconeogenic genes) and increase insulin sensitivity (model-based).	May restrain NF-κB-driven pro-inflammatory activity; overall, ANGPTL8 inflammatory roles remain insufficiently defined.
**Follistatin (FST)** [735,736,739]	Liver	↑	Higher circulating FST associated with higher T2DM/MASLD susceptibility; promotes adipose/muscle IR and hepatic gluconeogenesis; reduction improves insulin sensitivity in mice.	May modulate inflammation via neutralizing TGF-β family ligands; can induce chemokines and NF-κB-linked cytokines (context- and dose-dependent).
**Adropin** [735,747,748]	Liver; brain	↓ in obesity/T2DM; ↑ in NAFLD reported	Improves glucose tolerance/insulin sensitivity in mice; in muscle, potentiates insulin signaling and shifts substrate preference toward glucose while suppressing FA oxidation.	Mild anti-inflammatory activity reported (e.g., constraint of TNF-α signaling); paradoxical rise in NAFLD suggests stage- or phenotype-dependence.
**Hepassocin** [735,736,737,739]	Liver; brown adipose	↑	Hepatocyte growth/regeneration factor; can promote IR (liver/muscle) and increase hepatic TAG accumulation.	During liver injury may protect against inflammation/steatosis/fibrosis/cell death, yet can also modestly increase cytokine production → dual, context-dependent.
**SMOC-1** [735,736,739]	Predominantly liver	↓	Increases insulin sensitivity by inhibiting hepatocyte cAMP formation (gluconeogenic restraint).	Proposed anti-inflammatory restraint via support of TGF-β pathway activity (mechanistic inference; evidence base should be stated if limited).
**IGF-1** [735,736,737,749]	Liver (major circulating source)	↓	Growth factor with insulin-sensitizing effects (mainly muscle; also adipose/liver); low-affinity InsRec binding; deficiency associates with metabolic deterioration.	Generally anti-inflammatory (limits cytokine production), but pro-inflammatory effects reported in certain contexts; pro-inflammatory cytokines can suppress IGF-1 secretion (bidirectional axis).
**GDF15** [735,736,739]	Liver	↑ (stress-responsive)	Increases energy expenditure and limits weight gain via thermogenesis/lipolysis/FA β-oxidation (often interpreted as compensatory stress hormone).	TGF-β superfamily member; induced by inflammatory cytokines (IL-1β, TNF-α, etc.); often a marker of cellular stress burden rather than a direct driver.
**LCN13 (lipocalin-13)** [735,750]	Liver; muscle (lower elsewhere)	↓	Anti-diabetic profile in models: inhibits lipogenesis/gluconeogenesis and promotes FA β-oxidation in hepatocytes; increases adipocyte insulin sensitivity.	Reduces hepatic steatosis in obese mice; overall anti-diabetic/anti-steatotic direction predominates in experimental settings.
**LPS-binding protein (LBP)** [735,751]	Predominantly liver	↑	Can initiate lipid-metabolic shifts and contribute to IR in metabolic endotoxemia settings (often associative).	Acute-phase protein: binds LPS and engages TLR4/CD14, promoting inflammation; mechanistic link fits gut-derived endotoxin models of meta-inflammation.
**SHBG** [736,752]	Liver	↓	Modulates sex hormone bioavailability; associated with reduced hepatic lipogenesis and protection against MASLD/NAFLD development (direction consistent but confounded by endocrine/metabolic covariates).	Can activate Kupffer/reticuloendothelial cells modestly; IL-1β and TNF-α suppress SHBG production (inflammation-to-SHBG axis).
**Selenoprotein P (SelP)** [736,739,753,754]	Liver	↑	Promotes hepatic/muscle IR; reduces β-cell insulin production; inhibits InsRec signaling; induced by glucose/FFA and suppressed by insulin.	
**Leukocyte-Cell Chemotaxin 2 (LECT2)** [739,755,756]	Predominantly hepatocytes; lower levels in adipose tissue and leukocytes	↑	Increases skeletal-muscle IR; circulating levels correlate positively with obesity severity and IR in humans.	Neutrophil chemoattractant; drives macrophage polarization toward M1. Upregulates NF-κB and IL-6 expression.

Note. AMPK, AMP-Activated Protein Kinase; ANGPTL, Angiopoietin-Related Protein; ATF4, Activating Transcription Factor 4; ER, Endoplasmic Reticulum; FetA, Fetuin-A; FFAs, Free Fatty Acids; FGF, Fibroblast Growth Factor; FST, Follistatin; GDF15, Growth Differentiation Factor 15; IGF-1, Insulin-Like Growth Factor 1; IL, Interleukin; InsRec, Insulin Receptor; IR, Insulin Resistance; LBP, Lipopolysaccharide Binding Protein; LCN13, Lipocalin 13; LECT2, Leukocyte-Cell Chemotaxin 2; LPS, Lipopolysaccharide (Endotoxin) of Gram-Negative Bacteria; M1, Classically Activated Macrophages; MASLD, Metabolically Associated Steatotic Liver Disease; NAFLD, Non-Alcoholic Fatty Liver Disease; NF-κB, Nuclear Factor kappa B; PPAR, Peroxisome Proliferator-Activated Receptor; Sel P, Selenoprotein P; SHBG, Sex Hormone-Binding Globulin; SMOC-1, Secreted Modular Calcium-Binding Protein 1; T2DM, Type 2 Diabetes Mellitus; TAG, Triacylglyceride; TGF-β, Transforming Growth Factor beta; TLR, Toll-Like-Receptor; TNF, Tumor Necrosis Factor; UPR, Unfolded Protein Response.

**Table 12:** Sites of Production, Blood Changes, Metabolic/Homeostatic Effects, and Links with Inflammation for Major Myokines (Skeletal-Muscle Histohormones) [757,758,759,760,761,762,763,764,765,766,767,768,769,770,771].

Myokine	Primary Production Site(s)	Blood Change in Obesity & T2DM	Metabolic/Homeostatic Effects	Links with Cellular-Stress Factors/Role in T2DM (Context-Dependent)
**Irisin**	Contracting skeletal muscle	**↓** (often reported)	Promotes GLUT4 translocation and oxidative metabolism; supports thermogenesis/browning; associated with improved skeletal-muscle insulin sensitivity; declines with age.	Activates AMPK-related programs; linked to lower ROS and improved metabolic phenotype in obesity/T2DM/MASLD; strength of human evidence varies across cohorts.
**SPARC (osteonectin)**	Contracting skeletal muscle	**Variable/insufficiently consistent**	Exercise-induced factor associated with muscle remodeling and lipolysis; reported to improve insulin sensitivity in muscle and in peripheral tissues in experimental settings.	Frequently linked to AMPK activation and metabolic protection; however, pro-inflammatory actions in adipose tissue have been reported → bidirectional, context-dependent.
**BAIBA (β-aminoisobutyric acid)**	Contracting skeletal muscle	**Variable/insufficiently consistent**	In models, increases FA oxidation and reduces lipogenesis; activates PPARδ in muscle and PPARα-linked hepatic FA β-oxidation; improves IR.	Reported to constrain NF-κB activation and pro-inflammatory stress signaling; evidence base is predominantly mechanistic/animal with limited clinical standardization.
**BDNF**	Skeletal muscle after exercise; brain	**↓** (often reported in metabolic disease)	Supports oxidative phenotype in muscle; promotes lipid oxidation and insulin sensitivity; participates in muscle glucose–lipid homeostasis.	Signals via AMPKα, PI3K/Akt, PLCγ/PKC; may modulate intensity of inflammatory stress responses; directionality in T2DM is not fully uniform across tissues.
**IL-6**	Contracting skeletal muscle (also many cells)	**Acute** **↑** **with exercise; chronic** **↑** **in low-grade inflammation; in T2DM: variable**	Acute IL-6 can enhance glucose uptake and lipid oxidation; chronic elevation is associated with impaired insulin signaling and IR; also modulates β-cell secretion.	Exercise-related IL-6 acts as a myokine with transient, potentially beneficial metabolic signaling; persistent IL-6 is a marker/mediator of meta-inflammation and may exacerbate IR → timing-dependent effects.
**IL-13**	Contracting skeletal muscle (reported); immune sources	**↓ or N** (variable)	Limits hepatic gluconeogenesis; may improve hepatic insulin sensitivity.	Th2-associated cytokine with moderate anti-inflammatory bias; in metabolic disease, effects depend on immune milieu and tissue context.
**IL-15**	Contracting skeletal muscle (reported)	**↓ or N** (variable)	In models, reduces adiposity and supports muscle insulin sensitivity; may influence energy expenditure.	Proposed protective factor against obesity/T2DM sequelae; human evidence is heterogeneous and may reflect training status and body composition.
**Myonectin (CTRP15)**	Skeletal muscle	**↓/↑** **(variable across studies)**	Improves muscle insulin sensitivity; promotes FFA uptake in adipocytes/hepatocytes; implicated in sarcopenia prevention.	Often interpreted as metabolically protective; the direction of circulating change and clinical associations are inconsistent, likely phenotype-dependent.
**Myostatin**	Skeletal muscle; myocardium; adipocytes	**↑** (often reported)	Inhibits muscle growth/differentiation; reduces lean mass; associated with worsened insulin sensitivity in muscle/adipose tissue.	Linked to oxidative stress, sarcopenia and impaired glucose utilization; net effect generally unfavorable in obesity/T2DM, though magnitude varies with age and muscle status.
**GDF11 (BMP11)**	Skeletal muscle (reported)	**↓ or N** (variable)	TGF-β family factor; reported to improve insulin sensitivity in muscle, liver, adipose tissue in models.	Protective effects mainly supported by animal studies; translational relevance and direction in humans remain insufficiently defined.

Note. AMPK, AMP-Activated Protein Kinase; BAIBA, β-Aminoisobutyric Acid; BDNF, Brain-Derived Neurotrophic Factor; FA, Fatty Acids; FFAs, Free Fatty Acids; GDF15, Growth Differentiation Factor 15; GLUT, Glucose Transporter; IL, Interleukin; IR, Insulin Resistance; MASLD, Metabolically Associated Steatotic Liver Disease; N, Norm; NAFLD, Non-Alcoholic Fatty Liver Disease; NF-κB, Nuclear Factor kappa B; PI3K, Phosphoinositide 3-Kinases; PKC, Protein Kinase C; PLC, Phospholipase C; PPAR, Peroxisome Proliferator-Activated Receptor; ROS, Reactive Oxygen Species; SM, Skeletal Muscles; SPARC, Secreted Protein Acidic and Rich in Cysteine; T2DM, Type 2 Diabetes Mellitus; TGF-β, Transforming Growth Factor beta. FGF21, IGF-1, and adiponectin, which have been previously discussed, are also myokines that are produced in greater quantities during muscle activity.

**Table 13:** The Role of Scavenger Receptors in the Development of Insulin Resistance.

Receptor	Main Expressing Cells (Concise)	Principal Ligands (Examples)	Links with Insulin Resistance (Concise; Non-Universal Causality)	Evidence Base (Dominant; Illustrative Refs)
**SR-A1 (MSR1, CD204)**	Macrophages (incl. adipose tissue), monocytes/DC; endothelium/VSMC	Modified LDL (oxLDL, acLDL), AGEs, HSPs, nucleic acids, PAMPs	In humans, higher SR-A1 on adipose macrophages is associated with IR. In diet-induced obesity models, Msr1 deletion can worsen adipose insulin-stimulated glucose uptake, yet ligand exposure can aggravate IR in wild-type but not knockout animals → direction is context-dependent. In liver macrophages, can cooperate with TLR2/4 to activate JNK/TNF-α signaling, contributing to MASLD/NAFLD.	**M/A + H; conflicting** [1061,1062,1063,1064,1065,1066,1067]
**SR-B1 (SCARB1, CD36L1)**	Hepatocytes, macrophages, adrenal cells	HDL/modified HDL, oxLDL, PAMPs; HCV entry co-factor	Genetic associations with IR reported. In obese mice, SR-B1 deficiency impairs lipid handling and insulin signaling with steatosis/obesity, consistent with an overall protective role against IR (tissue- and model-dependent).	**M/A + H (genetic/assoc.)** [1068,1069,1070,1071]
**SR-B2 (CD36)**	Macrophages, endothelium, hepatocytes, adipocytes, skeletal muscle	FFAs (LCFAs), oxLDL/oxidized lipids, AGEs, thrombospondin	In obesity/T2DM, CD36 upregulation in macrophages and endothelium promotes lipid uptake and inflammatory signaling. Excess FFA/oxLDL uptake drives intracellular DAG/ceramide accumulation with PKC/serine-kinase activation, plausibly impairing insulin signaling → mechanistically pro-IR in many settings.	**M/A + H; strong mechanistic plausibility** [1072,1073,1074,1075,1076,1077]
**SR-D1 (CD68)**	Macrophages/monocytes/DC	oxLDL, apoptotic cells, modified LDL	CD68 is primarily a macrophage burden marker. Higher adipose CD68 correlates with TNF-α/IL-6 production and IR severity, consistent with macrophage-driven meta-inflammation rather than a receptor-specific causal pathway.	**H assoc. + M/A supportive** [1078,1079,1080]
**SR-E1 (LOX-1)**	Endothelium, VSMC, macrophages, platelets; adipocytes	oxLDL, AGEs, CRP, apoptotic products, HSPs	In obesity/T2DM, oxLDL can upregulate LOX-1 in adipocytes/endothelium, linking lipid stress to inflammatory activation and adipocyte IR. Adiponectin-associated downregulation suggests counter-regulatory control. Also implicated in endotheliosis/atherosclerosis, indirectly reinforcing cardiometabolic risk.	**M/A + H; predominantly pro-IR associations** [1061,1081,1082]
**SR-E3 (CD206, MRC1)**	M2 macrophages; DC	Microbial glycans, altered glycoproteins, collagen, HSP70	CD206 marks heterogeneous “M2-like” populations. In obesity, CD206^+^CD11c^+^ monocyte-derived cells show higher inflammatory output and associate with IR, whereas CD206^+^CD11c^−^ macrophages support tissue repair and homeostasis → interpretation requires phenotypic stratification; CD206 per se is not uniformly protective.	**H + M/A; phenotype-dependent** [1083,1084,1085,1086]
**SR-I1 (CD163)**	M2/M(Hb) macrophages; microglia; monocytes	Hb–haptoglobin complexes; hemoglobin; fibronectin; PAMPs	Soluble CD163 is reported as a risk marker for IR/incident T2DM in cohorts. Membrane CD163 clears Hb–Hp and limits oxidative damage; CD163 deficiency can aggravate IR in models → biomarker elevation does not imply pathogenic direction (may reflect compensatory activation).	**H (biomarker) + M/A (protective function)** [1087,1088,1089,1090,1091,1092]
**SR-J1 (RAGE)**	Macrophages/DC; endothelium; adipocytes; hepatocytes; neurons	AGEs; HMGB1/S100; DAMPs; amyloids; modified proteins	AGE/HMGB1–RAGE signaling activates NF-κB and supports adipose inflammation, providing a plausible mechanistic bridge to IR in obesity. Hypoxia can upregulate RAGE in adipocytes; macrophage–adipocyte cross-talk via RAGE is proposed. Genetic signals exist, but may reflect LD with nearby loci → causal inference should be cautious.	**M/A + H; mechanistic + associative** [379,1093,1094,1095,1096,1097,1098,1099,1100]
**SR-K1 (CD44)**	Macrophages; lymphocytes; adipocytes; skeletal muscle; liver cells	Hyaluronan, osteopontin, ECM proteins	Soluble CD44 is elevated in IR and correlates with adipose inflammatory gene signatures (CD68, IL-6, OPN). OPN/CD44 signaling promotes M1 polarization; CD44-linked cytoskeletal signaling can impair IRS-1 phosphorylation and promote adipogenesis/obesity and skeletal-muscle IR; also associated with liver inflammation/fibrosis.	**H assoc. + M/A mechanistic** [1101,1102,1103,1104]
**SR-L1 (LRP1)**	Hepatocytes, adipocytes, muscle; Kupffer cells; endothelium; CNS cells	ApoE/oxLDL; protease complexes; C1q; integrins; growth factors	Tissue-specific LRP1 loss impairs insulin signaling and lowers GLUT3/GLUT4; knockout phenotypes include IR with dyslipidemia/steatosis and reduced InsRec/GLUT2 in liver → generally supportive of insulin sensitivity via trafficking/signaling roles. However, high Kupffer-cell LRP1 can promote hepatic inflammation and glucose intolerance via Wnt5a → dual, cell-type–dependent effects.	**M/A predominant; context-dependent** [1105,1106,1107,1108,1109,1110,1111]
**CD14 (TLR co-receptor)**	Macrophages/monocytes; neutrophils	LPS–LBP complex; peptidoglycans; LTA	As a TLR4/TLR2 co-receptor, mediates robust innate activation. CD14–TLR signaling in liver/adipose promotes IR; CD14 deletion attenuates obesity-related metabolic and cardiovascular complications in mice → consistent pro-IR role in endotoxin-driven meta-inflammation.	**M/A strong; H supportive** [1112,1113]

Note. acLDL, Acetylated Low Density Lipoproteins; AGE, Advanced Glycation End Products; CRP, C-reactive Protein; CVD, cardiovascular disease; DAG, Diacylglycerol; DC, Dendritic Cells; dsRNA, Double-Stranded Viral RNA; FFAs, Free Fatty Acids; GLUT, Glucose Transporter; Hb, hemoglobin; HDL, High-Density Lipoprotein; HMGB1, High-Mobility Group Box 1; Hp, haptoglobin; HSP, Heat-Shock Protein (family: HSP70, HSP90, HSP110); IL, Interleukin; InsRec, Insulin Receptor; IR, Insulin Resistance; IRS, Insulin Receptor Substrates; JNK, c-Jun N-terminal Kinase; LBP, Lipopolysaccharide Binding Protein; LDL, Low Density Lipoproteins; LOX-1, Lectin-like Oxidized Low-Density Lipoprotein Receptor-1; LPS, Lipopolysaccharide (Endotoxin) of Gram-Negative Bacteria; LRP1, LDL Receptor-related Protein-1 (CD91); M1, Classically Activated Macrophages; M2, Alternatively Activated Macrophages; MASLD, Metabolically Associated Steatotic Liver Disease; MSR1, Macrophage Scavenger Receptor 1; NAFLD, Non-Alcoholic Fatty Liver Disease; NF-κB, Nuclear Factor kappa B; OPN, Osteopontin; oxLDL, Oxidized Low-Density Lipoprotein; PAMPs, Pathogen-Associated Molecular Patterns; PDGF, Platelet-Derived Growth Factor; PKC, Protein Kinase C; RAGE, Receptor for Advanced Glycation End Products; ROS, Reactive Oxygen Species; SCARB1, Scavenger Receptor B Class I; SR, Scavenger Receptor; T2DM, Type 2 Diabetes Mellitus; TGF-β, Transforming Growth Factor beta; TLR, Toll-Like-Receptor; TNF, Tumor Necrosis Factor.

## Data Availability

No new data were created or analyzed in this study. Data sharing is not applicable to this article.

## Abbreviations

AC	Adenylate Cyclase
acLDL	Acetylated Low-Density Lipoproteins
ADK	Adenosine Kinase
ADP	Adenosine-5′-Diphosphate
AGEs	Advanced Glycation End Products
ALR	Absent in Melanoma 2 (AIM2)-Like Receptor
ALX	Lipoxin A4 Receptor
AMP	Adenosine-5′-Monophosphate
AMPK	AMP-Activated Protein Kinase
AngII	Angiotensin II
ANGPTL	Angiopoietin-Related Protein
AP-1	Activator Protein-1
APJ	G Protein-Coupled Receptor
AS160	Akt Isoforms
ATF4	Activating Transcription Factor 4
ATM	Ataxia-Telangiectasia Mutated Kinase
ATMs	Adipose Tissue Macrophages
ATP	Adenosine-5′-Triphosphate
ATR	ATM and Rad3-Related Kinase
BAIBA	β-Aminoisobutyric Acid
BAs	Bile Acids
BDNF	Brain-Derived Neurotrophic Factor
Breg	Regulatory B cells
CB1R	Cannabinoid Receptor 1
CVD	Cardiovascular Disease
CNS	Central Nervous System
CRP	C-reactive Protein
CS	Cellular Stress
CTL	Cytotoxic T Lymphocytes
DAG	Diacylglycerol
DAMPs	Danger-Associated Molecular Patterns
DC	Dendritic Cells
DDR	DNA Damage Response
dsRNA	Double-Stranded Viral RNA
eNOS	Endothelial Nitric Oxide Synthase
ER	Endoplasmic Reticulum
ERAD	Endoplasmic Reticulum-Associated Degradation
ERK	Extracellular Signal–Regulated Protein Kinase
ESRD	End Stage Renal Disease
ET-1	Endothelin-1
FA	Fatty Acid
FABP	Fatty Acid Binding Proteins
FABPpm	Plasma Membrane Fatty Acid-Binding Protein
FAT	Fatty Acid Translocase
FATP	Fatty Acid Transporter Proteins
FetA	Fetuin-A
FFA	Free Fatty Acid
FFAR	Free Fatty Acid Receptor
FGF	Fibroblast Growth Factor
FOXO	Forkhead Box O Proteins
FR	Free Radical
FST	Follistatin
G-CSF	Granulocyte Colony-Stimulating Factor
GM-CSF	Granulocyte-Macrophage Colony-Stimulating Factor
GDF15	Growth Differentiation Factor 15
GLP-1	Glucagon-Like Peptide-1
GLUT	Glucose Transporter
GPCRs	G-Protein-Coupled Receptors
GRP	Glucose-Regulated Protein
GSK-3	Glycogen Synthase Kinase-3
GTP	Guanosine 5′-Triphosphate
GWAS	Genome-Wide Association Studies
Hb	Hemoglobin
HbA1c	Glycated Hemoglobin
HCV	Hepatitis C Virus
HDL	High-Density Lipoprotein
HEV	High Endothelial Venules
HHV	Human Herpesvirus
HIF-1	Hypoxia-Inducible Factor1
HIV	Human Immunodeficiency Virus
HMGB1	High-Mobility Group Box 1
HMOX1	Heme-Oxygenase 1
HNF1α	Human Hepatocyte Nuclear Factor-1 alpha
HOMA2-IR	Homeostasis Model Assessment of Insulin Resistance, version 2nd
HOMA-B	Homeostasis Model Assessment for B-Cell Function
HOMA-IR	Homeostasis Model Assessment of Insulin Resistance
Hp	Haptoglobin
HSF	Heat Shock Factor
HSPs	Heat-Shock Proteins
I3P	Inositol Trisphosphate
ICAM	Intercellular Adhesion Molecule
IFN	Interferon
IGF	Insulin-Like Growth Factor
IL	Interleukin
IL-1RA	Interleukin-1 Receptor Antagonist
IL-1RAPL1	Interleukin-1 Receptor Accessory Protein-Like 1
IKBKB	Inhibitor of Nuclear Factor kappa B Kinase Subunit beta
iNOS	Inducible Nitric Oxide Synthase
InsRec	Insulin Receptor
IP3	Inositol Trisphosphate
IR	Insulin Resistance
IRS	Insulin Receptor Substrates
JAK	Janus Kinase
JNK	c-Jun N-terminal Kinase
LAM	Lipid Associated Macrophages
LBP	Lipopolysaccharide Binding Protein
LCFA	Long-Chain Fatty Acids
LCN13	Lipocalin 13
LDL	Low-Density Lipoproteins
LECT2	Leukocyte-Cell Chemotaxin 2
LOX-1	Lectin-like Oxidized Low-Density Lipoprotein Receptor-1
LPS	Lipopolysaccharide (Endotoxin) of Gram-Negative Bacteria
LRP1	LDL Receptor-related Protein-1 (CD91)
LX	Lipoxin
M1	Classically Activated Macrophages
M2	Alternatively Activated Macrophages
MAPK	Mitogen-Activated Protein Kinase
MARK	Microtubule Affinity-Regulating Kinase
MASLD	Metabolically Associated Steatotic Liver Disease
MCAi	Mcauley Index
MCP-1	Monocyte Chemoattractant Protein-1
MCS	Metabolic Cellular Stress
MCDs	Microcirculatory Disorders
MD2	Myeloid Differentiation Protein 2
MIF	Macrophage Migration Inhibitory Factor
miRNA	MicroRNA
MMe	Metabolically Activated Macrophages
MMP	Matrix Metalloproteinase
MetS	Metabolic Syndrome
MS	Mitochondrial Stress
MSR1	Macrophage Scavenger Receptor 1
mTOR	Mammalian Target of Rapamycin
NAFLD	Non-Alcoholic Fatty Liver Disease
NDPK	Nucleoside Diphosphate Kinases
NETs	Neutrophil Extracellular Traps
NFE2	Nuclear Factor Erythroid 2
NF-κB	Nuclear Factor kappa B
NK	Natural Killer
NLR	Nucleotide-Binding Domain and Leucine-Rich Repeat Receptor
NLRP3	Nucleotide-Binding Oligomerization Domain-Like Receptor Family Protein 3
NRF2	Nuclear Factor Erythroid-2-Related Factor 2
OPN	Osteopontin
OS	Oxidative Stress
oxLDL	Oxidized Low-Density Lipoprotein
PAI-1	Plasminogen Activation Inhibitor of the First Type
PAMPs	Pathogen-Associated Molecular Patterns
PDGF	Platelet-Derived Growth Factor
PI3K	Phosphoinositide 3-Kinases
PKA	Protein Kinase A
PKB/Akt	Protein Kinase B
PKC	Protein Kinase C
PLC	Phospholipase C
PP	Protein Phosphatase
PPARs	Peroxisome Proliferator-Activated Receptors
PRR	Pattern Recognition Receptor
PTP	Protein Tyrosine Phosphatase
PYY	Peptide YY
QUICKI	Quantitative Insulin Sensitivity Check Index
RAGE	Receptor for Advanced Glycation End Products
RAS	Rat Sarcoma Viral Oncogene Homolog
RBP4	Retinol Binding Protein 4
ROS	Reactive Oxygen Species
RTK	Receptor Tyrosine Kinases
SCFA	Short-Chain Fatty Acid
Sel P	Selenoprotein P
SFRP5	Secreted Frizzled-Related Protein 5
SGLT	Sodium–Glucose co-Transporter
SHBG	Sex Hormone-Binding Globulin
SIRT1	Sirtuin 1
SLE	Systemic Lupus Erythematosus
SM	Skeletal Muscles
SMOC-1	Secreted Modular Calcium-Binding Protein 1
SPARC	Secreted Protein Acidic and Rich in Cysteine
SPMs	Specialized Proresolving Mediators
SR	Scavenger Receptor
STAT	Signal Transduction and Activator of Transcription
T2DM	Type 2 Diabetes Mellitus
TAG	Triacylglycerol
TF	Transcription Factor
TGF-β	Transforming Growth Factor beta
Th	T-helper
TKD	Tyrosine Kinase Domain
TLR	Toll-Like-Receptor
TNF	Tumor Necrosis Factor
TRAF2	Tumor Necrosis Factor Receptor Associated Factor 2
Treg	CD4^+^ Regulatory T Cells
TyG	Triglyceride-Glucose Index
TYK	Tyrosine Kinase
UPR	Unfolded Protein Response
UPR_ER_	Unfolded Protein Response of the Endoplasmic Reticulum
UPRmt	Mitochondrial Unfolded Protein Response
VCAM	Vascular Cell Adhesion Molecule
VEGF	Vascular Endothelial Growth Factor
VFA	Visceral Fat Area
WC	Waist Circumference
